# Supplementary material for: Discovery of New VEGFR-2 Inhibitors: Design, Synthesis, Anti-Proliferative Evaluation, Docking, and MD Simulation Studies
Source: Molecules. 2022 Sep 21;27(19):6203. doi: 10.3390/molecules27196203 (PMC9571953; doi:10.3390/molecules27196203)
Supplement: Supplementary file 1 [file molecules-27-06203-s001.zip › molecules-1897608-supplementary.pdf]

Discovery of new VEGFR-2 inhibitors: Design, synthesis, anti-proliferative evaluation, docking, and MD simulation studies

Eslam B. Elkaeed[a], Reda G.Yousef[b], Mohamed M. Khalifa[b], Albaraa Ibrahim[b], Ahmed B. M. Mehany[c], Ibraheem M. M. Gobaara[c], Bshra A. Alsfouk[d], Wagdy M. Eldehna[e,f], Ahmed M. Metwaly[g,h], Ibrahim. H. Eissa[a]*, Mohamed A. El-Zahabi [a]*

[a] Department of Pharmaceutical Sciences, College of Pharmacy, AlMaarefa University, Riyadh 13713, Saudi Arabia.

[b] Pharmaceutical Medicinal Chemistry & Drug Design Department, Faculty of Pharmacy (Boys), Al-Azhar University, Cairo11884, Egypt.

[c] Zoology Department, Faculty of Science (Boys), Al-Azhar University, Cairo11884, Egypt.

[d] Department of Pharmaceutical Sciences, College of Pharmacy, Princess Nourah bint Abdulrahman University, P.O. Box 84428, Riyadh 11671, Saudi Arabia.

[e] School of Biotechnology, Badr University in Cairo, Badr City, Cairo 11829, Egypt

[f] Department of Pharmaceutical Chemistry, Faculty of Pharmacy, Kafrelsheikh University, P.O. Box 33516, Kafrelsheikh, Egypt.

[g] Pharmacognosy and Medicinal Plants Department, Faculty of Pharmacy (Boys), Al-Azhar University, Cairo 11884, Egypt.

[h]Biopharmaceutical Products Research Department, Genetic Engineering and Biotechnology Research Institute, City of Scientific Research and Technological Applications (SRTA-City), Alexandria, Egypt

***Corresponding authors:**

Ibrahim H. Eissa

Pharmaceutical Medicinal Chemistry & Drug Design Department, Faculty of Pharmacy (Boys), Al-Azhar University, Cairo11884, Egypt**. Email:** Ibrahimeissa@azhar.edu.eg

Mohamed A. El-Zahabi

Pharmaceutical Medicinal Chemistry & Drug Design Department, Faculty of Pharmacy (Boys), Al-Azhar University, Cairo11884, Egypt. **Email:** malzahaby@yahoo.com

Content

1.	**Chemistry and materials**
2.	**Biological testing**<br>✓ *In vitro* anti-proliferative activity<br>✓ *In vitro* VEGFR-2 kinase assay<br>✓ Flow cytometry analysis for cell cycle<br>✓ Flow cytometry analysis for apoptosis<br>✓ Quantitative Real Time Reverse-Transcriptase PCR technique
3.	***In silico* studies**<br>✓ Docking studies<br>✓ ADMET studies<br>✓ Toxicity studies<br>✓ MD simulation<br>✓ MMPBSA
4.	***Raw data of VEGFR-2 assay***
5.	**Spectral data**
6.	**Report of in silico toxicity studies**

1- Chemistry and material

All melting points were carried out by open capillary method on a Gallen kamp Melting point apparatus. The infrared spectra were recorded on pye Unicam SP 1000 IR spectrophotometer using potassium bromide disc technique. Proton magnetic resonance 1HNMR spectra were recorded on a Bruker 400 Megahertz-nuclear magnetic resonance (400 MHZ-NMR) spectrophotometer. Carbon-13 (C13) nuclear magnetic resonance (13CNMR) spectra were recorded on a Bruker 100 Megahertz-nuclear magnetic resonance (100 MHZ-NMR) spectrophotometer. Tetramethylsilane (TMS) was used as internal standard and chemical shifts were measured in δ scale one part per million (ppm). All compounds were within ±0.4 of the theoretical values. The reactions were monitored by thin-layer chromatography (TLC) using TLC sheets precoated with UV fluorescent silica gel Merck 60 F254 plates and were visualized using ultraviolet (UV) lamp and different solvents as mobile phases.

2. Biological testing

✓ *In vitro* anti-proliferative activity against HepG-2 and HCT-116

The cells were cultured in RPMI-1640 medium supplemented with 10% fetal bovine serum (FBS), penicillin (100 unit /mL) and streptomycin sulphate (100µg/mL) at 37 ºC in a 5% CO_2 incubator. Then, cells were dropped in 96-well plates at a density of 3-8 × 103 cells/well and incubated for two days in a 5% CO_2 incubator at 37 °C. Then, the cells were treated with the synthesized compounds and the cell cultures were continued incubated for 24 h. However, Different concentrations of the compound under test (0.0, 5.0, 12.5, 25.0 and 50 mg/ml) were added to the cell monolayer. Then, (3-[4,5-dimethylthiazol-2-yl]- 2,5-diphenyltetrazolium bromide) MTT solution (20 µl, 5mg/mL) was added to each well and incubated for additional 4 h. The formed purple crystals of MTT-formazan were dissolved in 100 µl DMSO each well; the absorbance of each well was measured at 570 nm using a plate reader (EXL 800, USA). All compounds were tested three times. The relative cell viability in percentage was calculated. The results for IC_{50} values of the active compounds are summarized in **Table 1**. The data represented the mean of three independent experiments in triplicate

and were expressed as means ± SD. The IC_{50} value was defined as the concentration at which 50% of the cells could survive.

✓ Assessment of VEGFR-2 inhibition

The in vitro inhibitory activities of the tested compounds against Human VEGFR-2 were evaluated using ELISA kit (Enzyme-Linked Immunosorbent Assay) with quantitation performed through the Alpha Screen® System (PerkinElmer, USA) according to manufacturer's instructions. The assay employs an anti-phosphotyrosine antibody (specific for VEGFR-2) seeded on a 96-well plate. 100 µL of the standard solution or the tested compound was added into the wells, and incubated over night at 4°C with gentle shaking. After the wells washed, 100µL of the prepared biotin antibody was added, and incubated at room temperature for additional 1h. After washing to move away unbound biotinylated antibody, 100µL of streptavidin solution was added to the wells then incubated for 45min. at room 48 temperature, followed by washing again. 100µL of TMB Substrate reagent was added and the color produced is proportional to the amount of the of VEGFR-2 bound. The stop solution was added, then the intensity of color is read at 450 nm by ELISA Reader (PerkinElmer) immediately. Percent inhibition was calculated by the comparison of compounds treated to control incubations, the concentration of the test compound causing 50% inhibition (IC_{50}) was calculated from the concentration inhibition response curve (concentrations on the X-axis and the absorbance on the Yaxis) and the data were compared with sorafenib as a standard VEGFR-2 inhibitor

✓ Cell cycle analysis

HCT-116 cells were grown in six-well plates (each one contains 2 x 105 cells per well) containing 10% foetal bovine serum and incubated for 24 h at 37ºC and 5% CO2. The medium was replaced with (DMSO 1% v/v) containing the 9.3 µM of compound **6**, then incubated for 48 h, collected and washed with cold phosphate buffered saline (PBS). After fixation of the collected cells with ice-cold absolute ethanol (70%), the cells were rinsed with PBS then stained with the DNA fluorochrome PI, kept for 15 min at 37ºC. Then samples were analyzed with a FACS Caliber flow cytometer.

✓ Detection of apoptosis

Apoptotic activity of compound **6** was evaluated using Annexin V-FITC/PI apoptosis detection kit. HCT-116 cells (2 x 105) were seeded and incubated with compound **6** for 48 h, trypsinized, collected and washed with phosphate-buffered saline (PBS) several times; HepG-2 cells were stained with

Annexin V fluorescein isothiocyanate (FITC) and counterstained with propidium iodide (PI) for 15 min at 37ºC in the dark using the apoptosis detection kit (BD Biosciences, San Jose, CA) according to the manufacturer's protocol. Then, Annexin V-FITC and PI binding were analyzed by a FACS Caliber flow cytometer

3- in silico studies

✓ Docking studies

Crystal structure of VEGFR-2 [PDB ID: 2OH4, resolution: 2.07 Å] was obtained from Protein Data Bank. The docking investigation was accomplished using MOE 2014 software. At first, the crystal structure of VEGFR-2 was prepared by removing water molecules. Only one chain was retained beside the co-crystallized ligand. Then, the selected chain was protonated and subjected to minimization of energy process. Next, the active site of the target protein was defined.

Structures of the synthesized compounds and sorafenib (as reference standard) were drawn using ChemBioDraw Ultra 14.0 and saved as MDL-SD format. Such file was opened using MOE to display the 3D structures which were protonated and subjected to energy minimization. Formerly, validation of the docking process was performed by docking the co-crystallized ligand against the isolated pocket of active site. The produced RMSD value indicated the validity of process. Finally, docking of the tested compounds was done through the dock option inserted in compute window. For each docked molecule, 30 docked poses were produced using ASE for scoring function and force field for refinement. The results of the docking process were then visualized using Discovery Studio 4.0 software.

✓ ADMET studies

ADMET descriptors (absorption, distribution, metabolism, excretion and toxicity) of the compounds were determined using Discovery studio 4.0. At first, the CHARMM force field was applied then the tested compounds were prepared and minimized according to the preparation of small molecule protocol. Then ADMET descriptors protocol was applied to carry out these studies.

✓ Toxicity studies

The toxicity parameters of the synthesized compounds were calculated using Discovery studio 4.0. Sorafenib was used as a reference drug. At first, the CHARMM force field was applied then the

compounds were prepared and minimized according to the preparation of small molecule protocol. Then different parameters were calculated from toxicity prediction (extensible) protocol.

✓ Molecular dynamics simulation

Molecular dynamics simulation of the protein-ligand complexes was performed using GROMACS 2021 and Linux 5.4 package. The GROMOS96 54a7 forcefield was selected as the force field for proteins and the ligand topologies were generated from the PRODRG server. All the complexes were solvated using simple point charge (SPC) water molecules in a rectangular box. To make the simulation system electrically neutral, required number of Na^{+} and Cl^{-} ions were added while 0.15 mol/L salt concentrations were set in all the systems. Using the steepest descent method, all the solvated systems were subjected to energy minimization for 5000 steps. Afterwards, NVT (constant number of particles, volume, and temperature) series, NPT (constant number of particles, pressure, and temperature) series, and the production run were conducted in the MD simulation. The NVT and the NPT series were conducted at a 300 K temperature and 1 atm pressure for the duration of 300 ps. V-rescale thermostat and Parrinello-Rahman barostat were selected of the performed simulation. Finally, the production run was performed at 300 K for a duration of 100 ns (nanoseconds). Thereafter, a comparative analysis was performed measuring root mean square deviation (RMSD), root mean square fluctuation (RMSF), radius of gyration (Rg), solvent accessible surface area (SASA) and hydrogen bonds to analyze their stability. The Xmgrace program was used to represent the analyses in the form of plots.

✓ MM/PBSA

The g_mmpbsa package of GROMACS was utilized to calculate the MM/PBSA (Molecular Mechanics/Poisson Boltzmann Surface Area) binding free energies followed by final MD production run to get a detailed overview of the molecular interactions between the protein and ligand. The free solvation energy (polar and nonpolar solvation energies) and potential energy (electrostatic and Van der Waals interactions) of each protein-ligand complex were analyzed to determine the total ΔG_{bind} of the complex. The binding energies were calculated using the following equation in this method:

$$\Delta G_{binding} = G_{complex} - (G_{protein} + G_{ligand})$$

Here, the $\Delta G_{binding}$ = the total binding energy of the protein-ligand complex, $G_{protein}$ = the binding energy of free protein, and G_{ligand} = the binding energy of unbounded ligand.

8

Best-fit values	
LogIC50	2.336
HillSlope	-0.5103
IC50	216.9

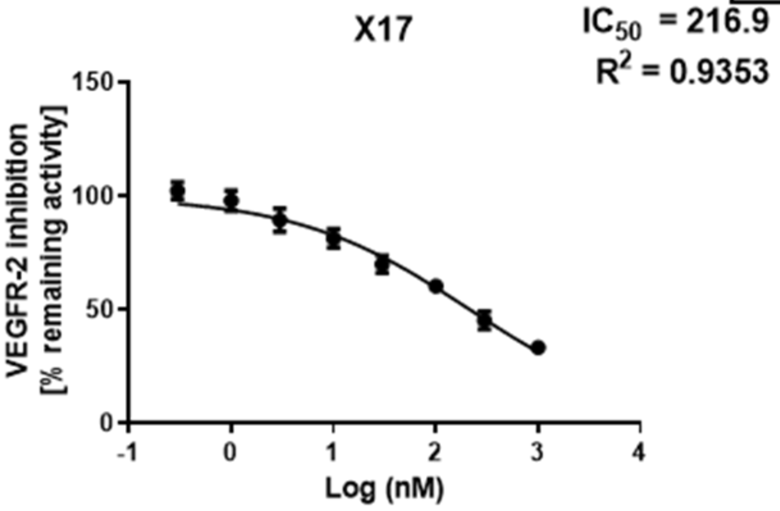

10

Best-fit values	
LogIC50	1.784
HillSlope	-0.4945
IC50	60.83

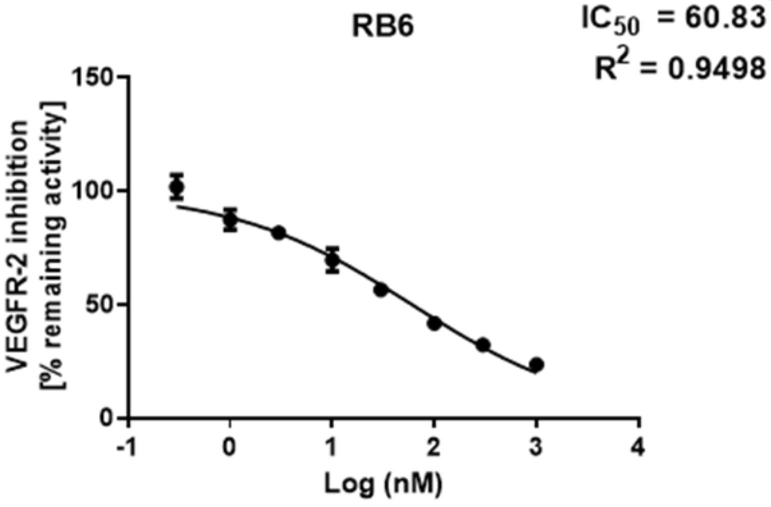

Best-fit values	
LogIC50	1.730
HillSlope	-0.4752
IC50	53.65

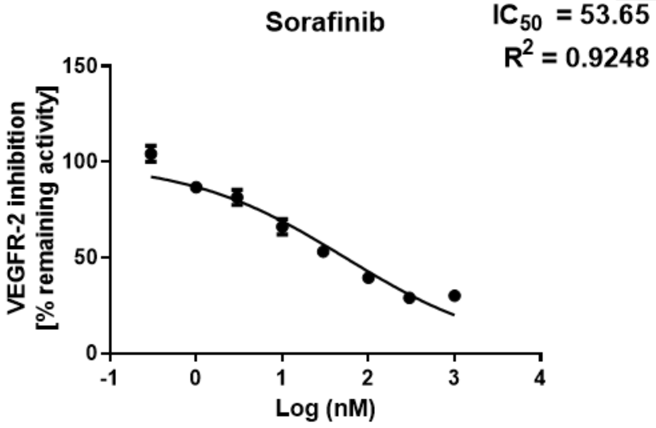

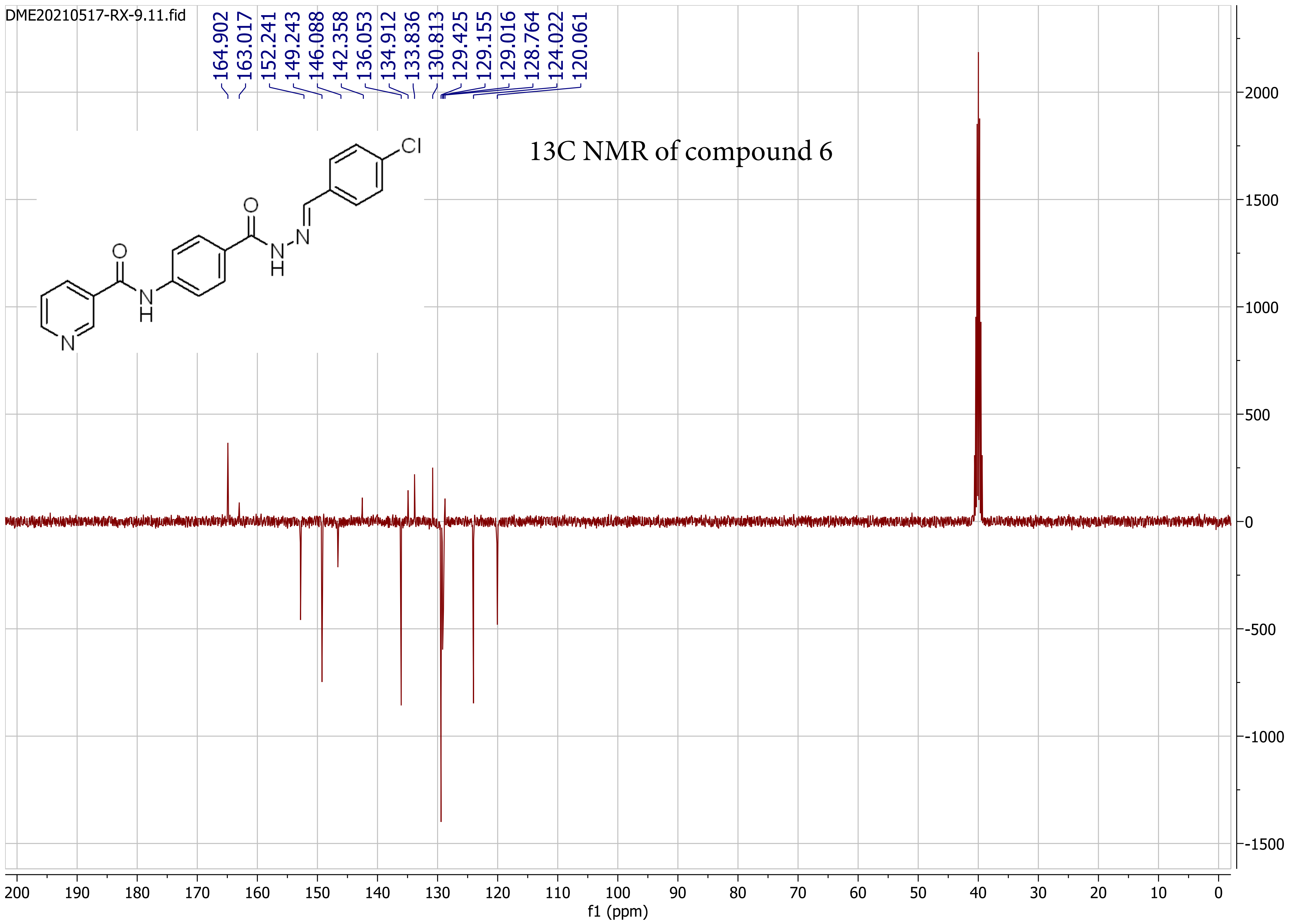
DME20210517-RX-9.11.fid
164.902
163.017
152.241
149.243
146.088
142.358
136.053
134.912
133.836
130.813
129.425
129.155
129.016
128.764
124.022
120.061
13C NMR of compound 6
Cl
O
N
N
H
O
N
H
N
2000
1500
1000
500
0
-500
-1000
-1500
200
190
180
170
160
150
140
130
120
110
100
90
80
70
60
50
40
30
20
10
0
f1 (ppm)

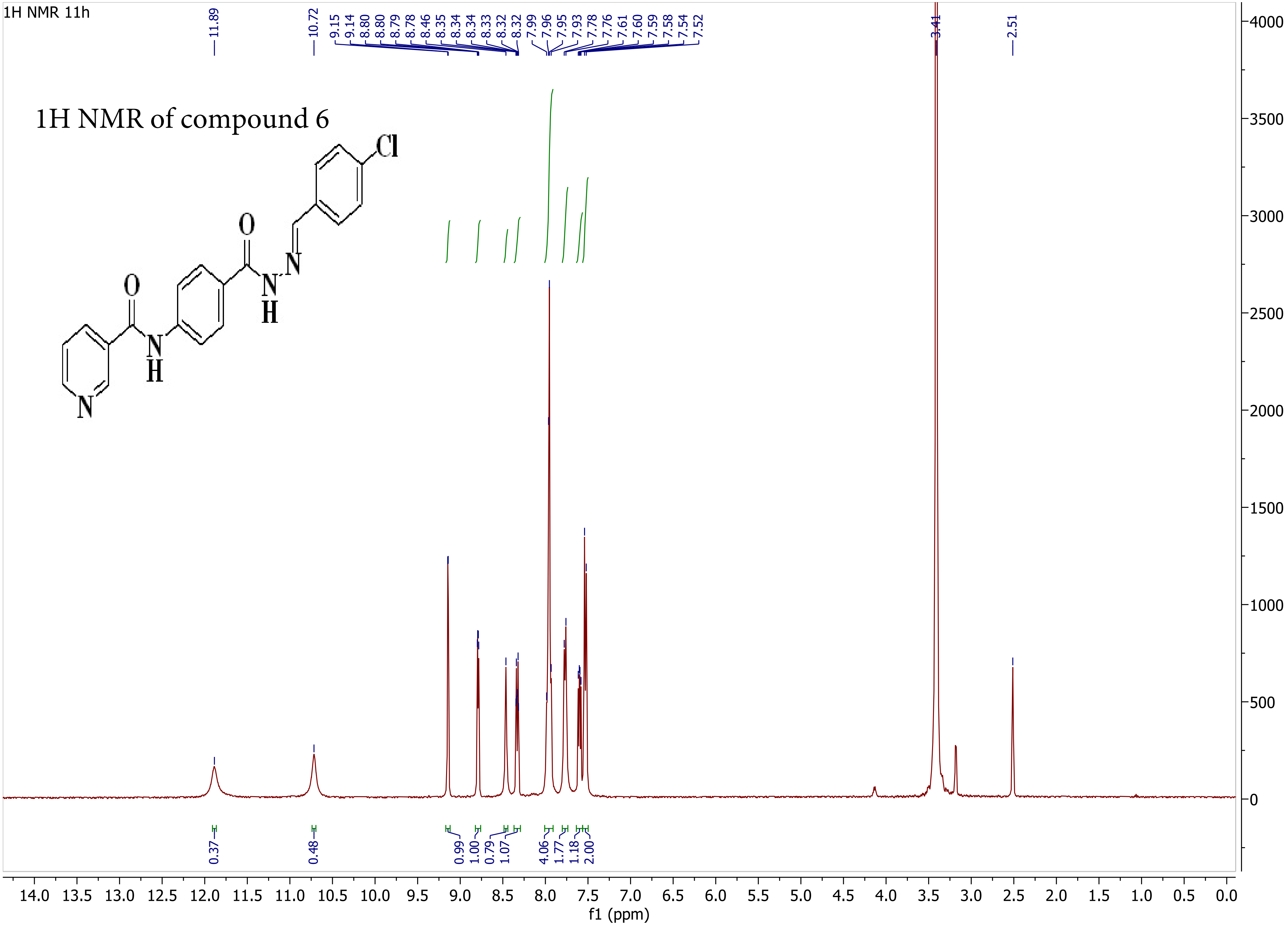

1H NMR 11h
1H NMR of compound 6
11.89
10.72
9.15
9.14
8.80
8.80
8.79
8.78
8.46
8.35
8.34
8.34
8.33
8.32
8.32
7.99
7.96
7.95
7.93
7.78
7.76
7.61
7.60
7.59
7.58
7.54
7.52
3.41
2.51
0.37
0.48
0.99
1.00
0.79
1.07
4.06
1.77
1.18
2.00
f1 (ppm)

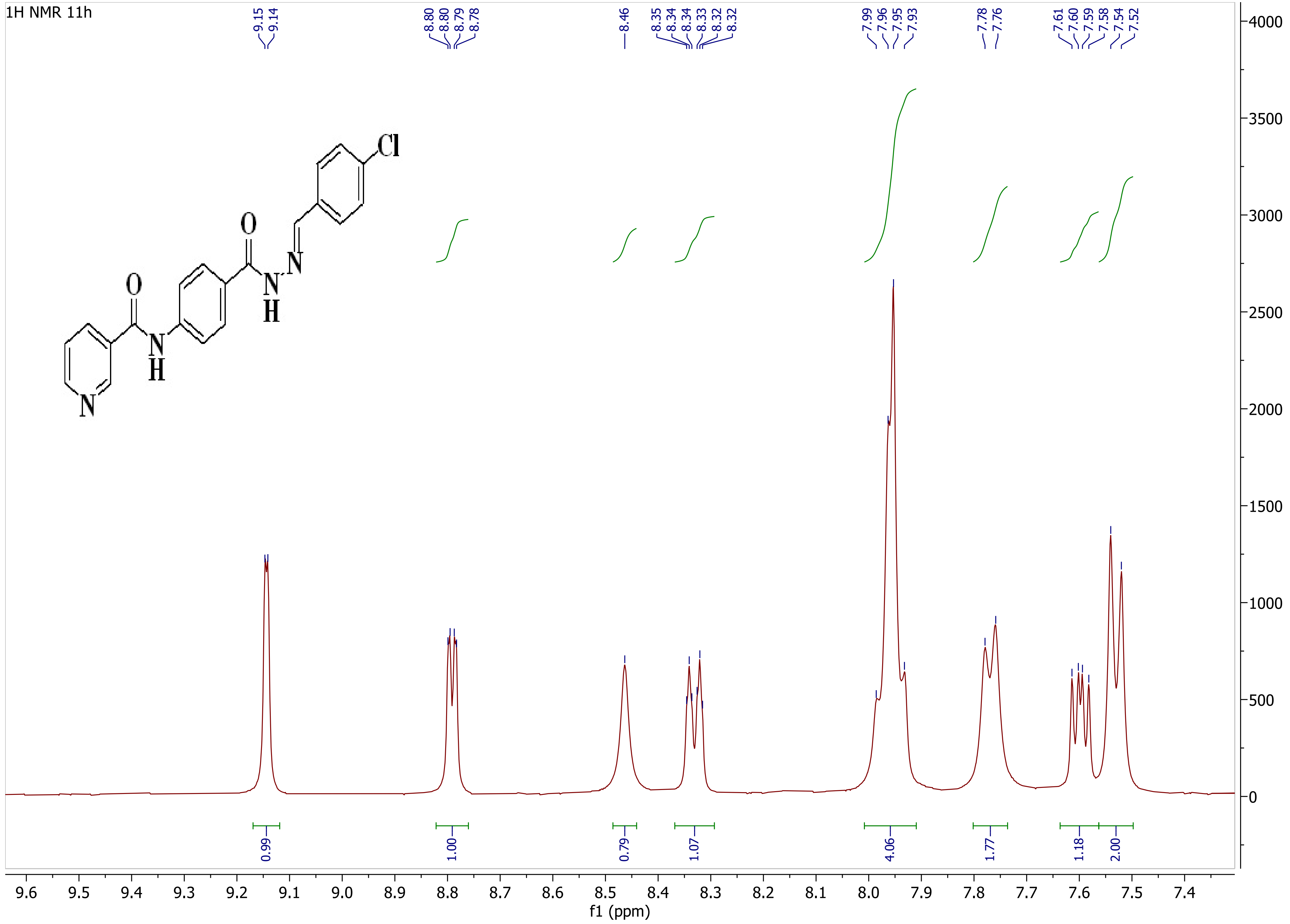

1H NMR 11h
9.15
9.14
8.80
8.80
8.79
8.78
8.46
8.35
8.34
8.34
8.33
8.32
8.32
7.99
7.96
7.95
7.93
7.78
7.76
7.61
7.60
7.59
7.58
7.54
7.52
0.99
1.00
0.79
1.07
4.06
1.77
1.18
2.00
f1 (ppm)
Cl
O
O
N
H
N
H
N
N

1H NMR of compound 7

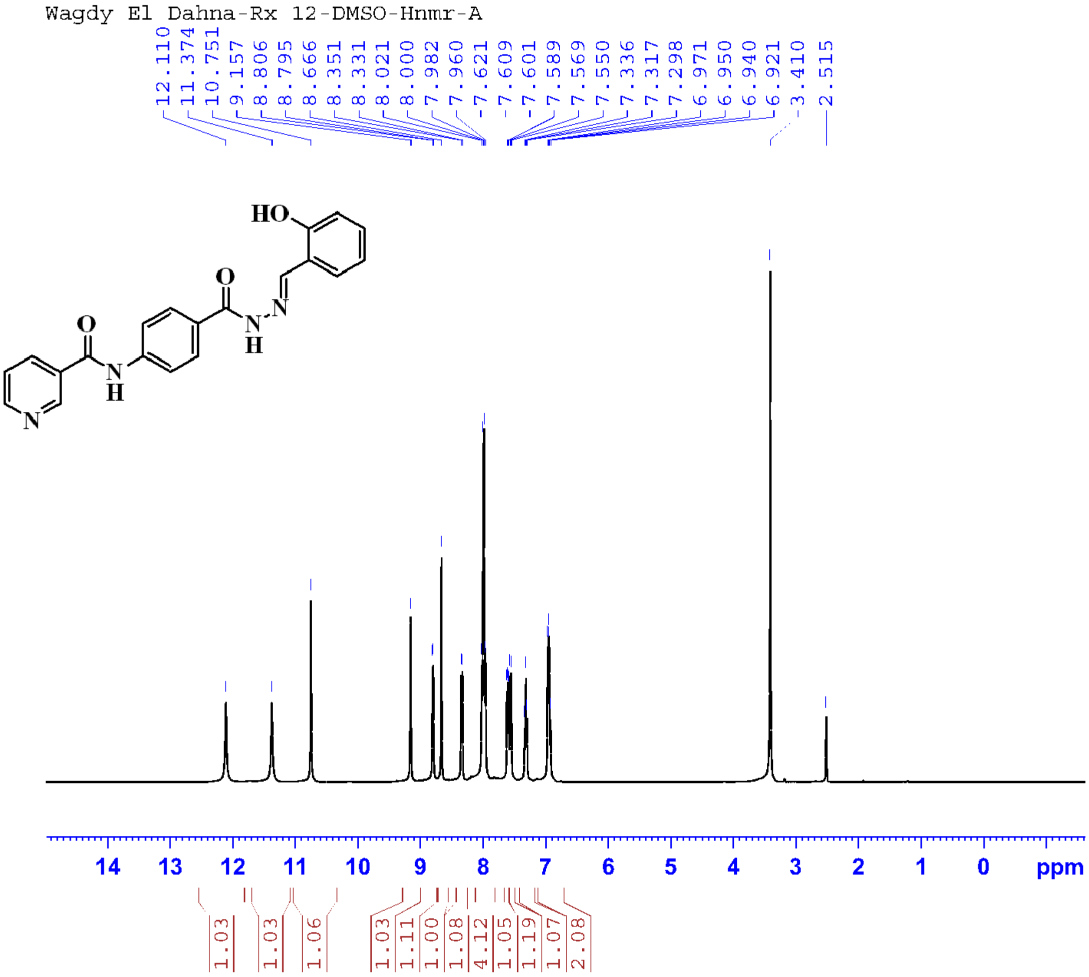

Faculty of Pharmacy

```
Current Data Parameters
NAME      Wagdy El Dahna-Rx 12-DMSO-
EXPNO                    10
PROCNO                    1

F2 - Acquisition Parameters
Date_              20210610
Time                  11.33 h
INSTRUM               spect
PROBHD     Z108618_0945 (
PULPROG                zg30
TD                    65536
SOLVENT                DMSO
NS                       16
DS                        2
SWH               8012.820 Hz
FIDRES            0.244532 Hz
AQ               4.0894465 sec
RG                     99.3
DW                   62.400 usec
DE                     6.50 usec
TE                    294.5 K
D1               1.00000000 sec
TD0                       1
SFO1            400.2024712 MHz
NUC1                     1H
P1                    13.50 usec
PLW1            13.00000000 W

F2 - Processing parameters
SI                    65536
SF              400.2000000 MHz
WDW                      EM
SSB                       0
LB                     0.30 Hz
GB                        0
PC                     1.00
```

1H NMR of compound 7

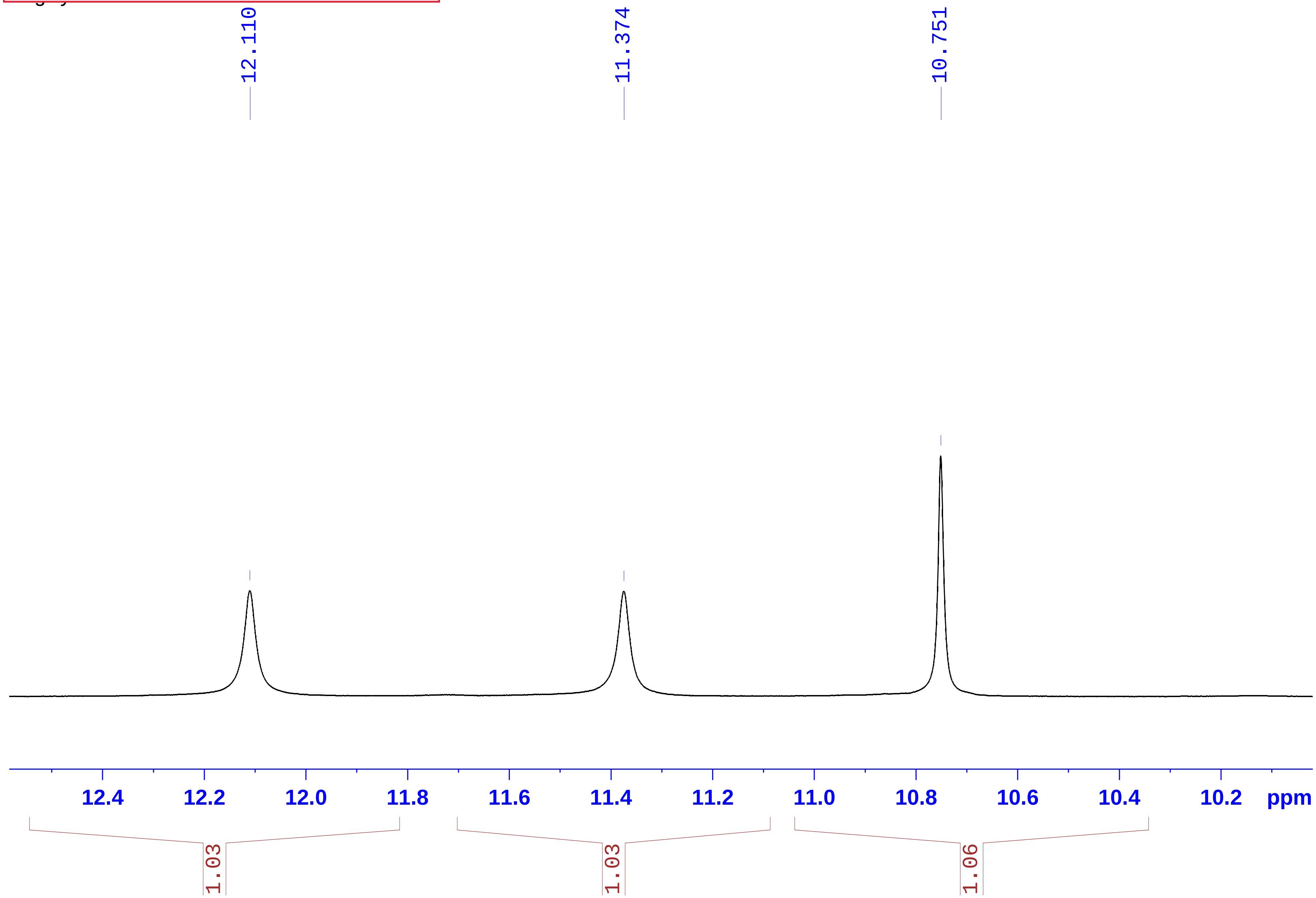

1H NMR of compound 7

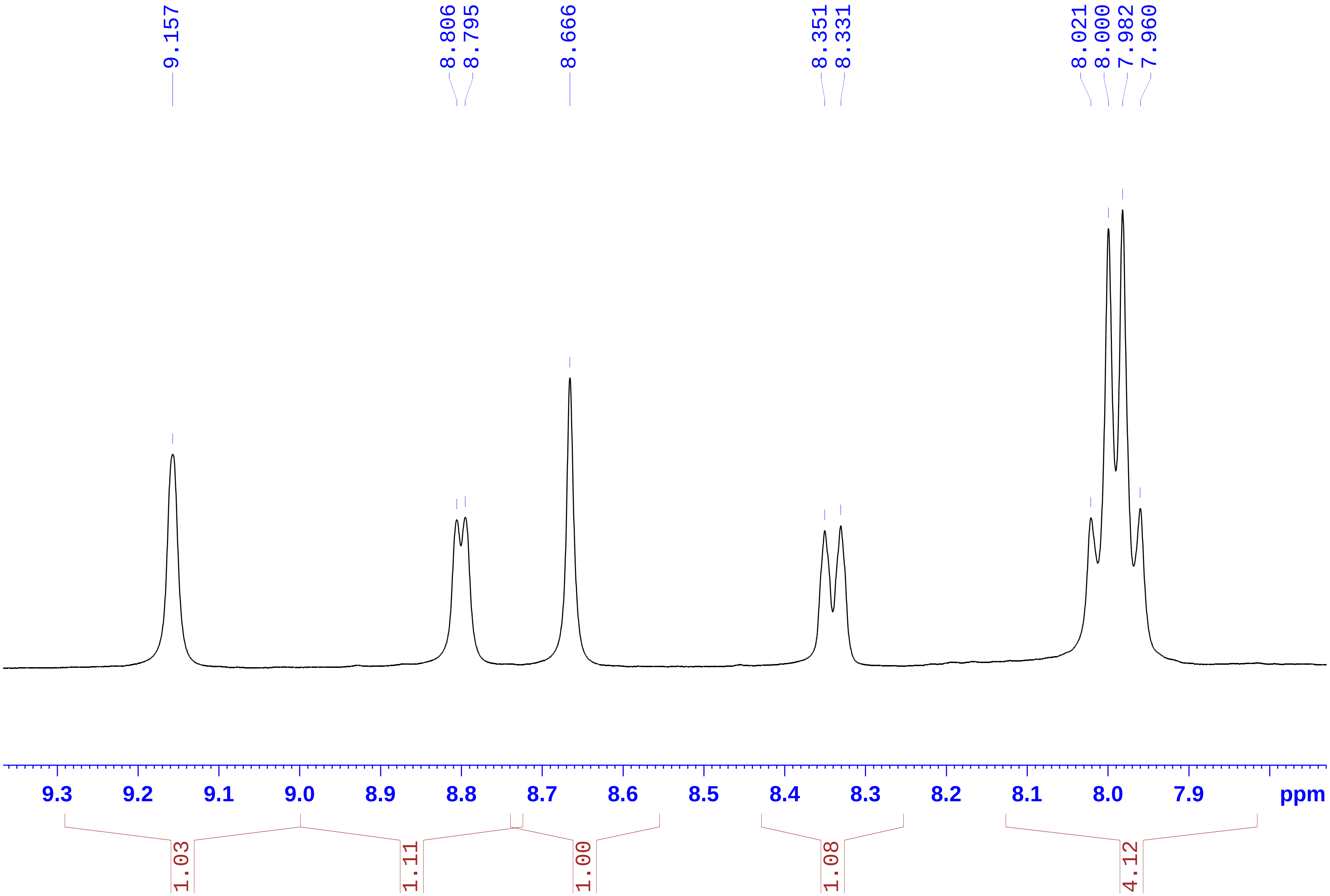

Wagdy El Dahna-Rx 12-DMSO-Hnmr-A

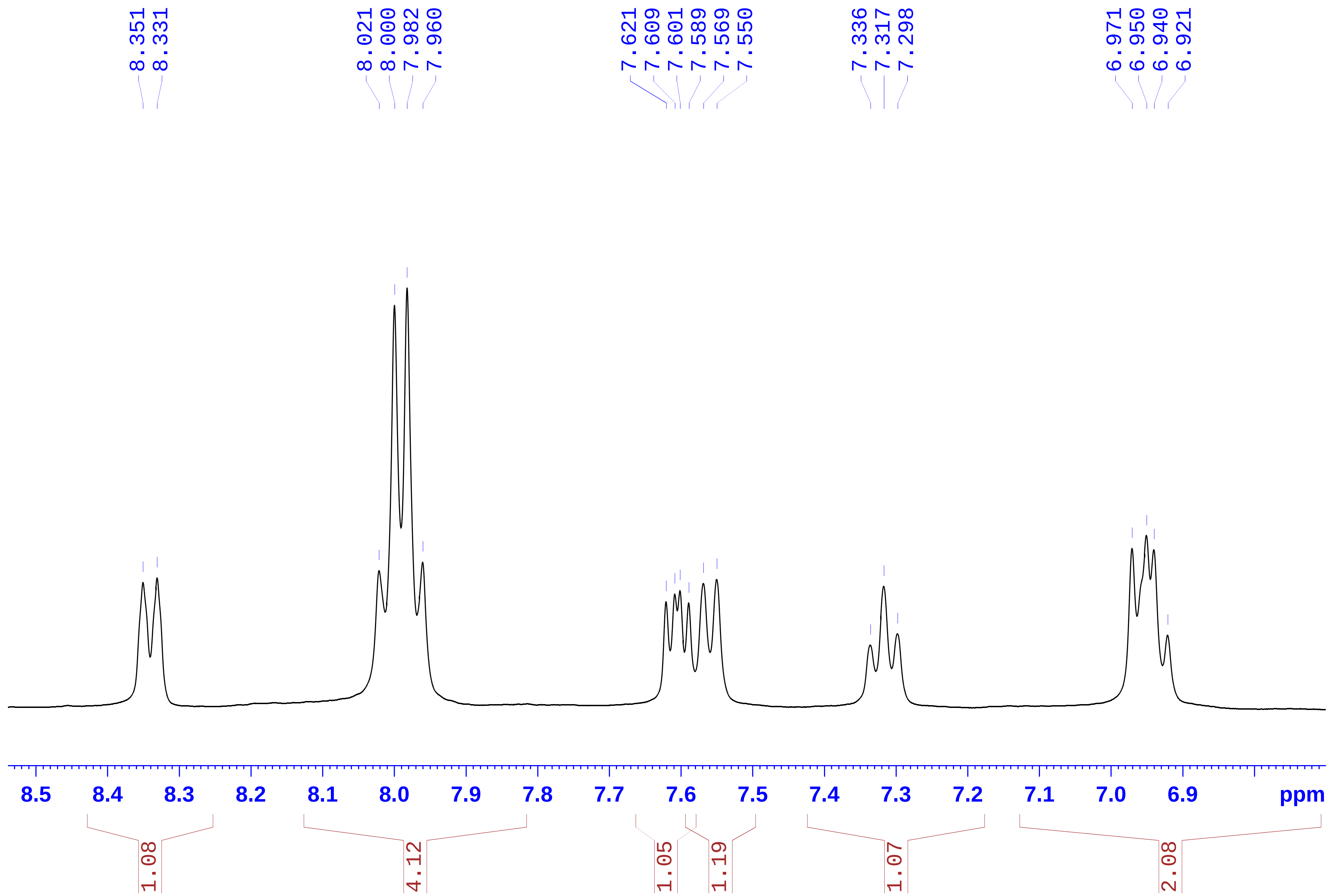

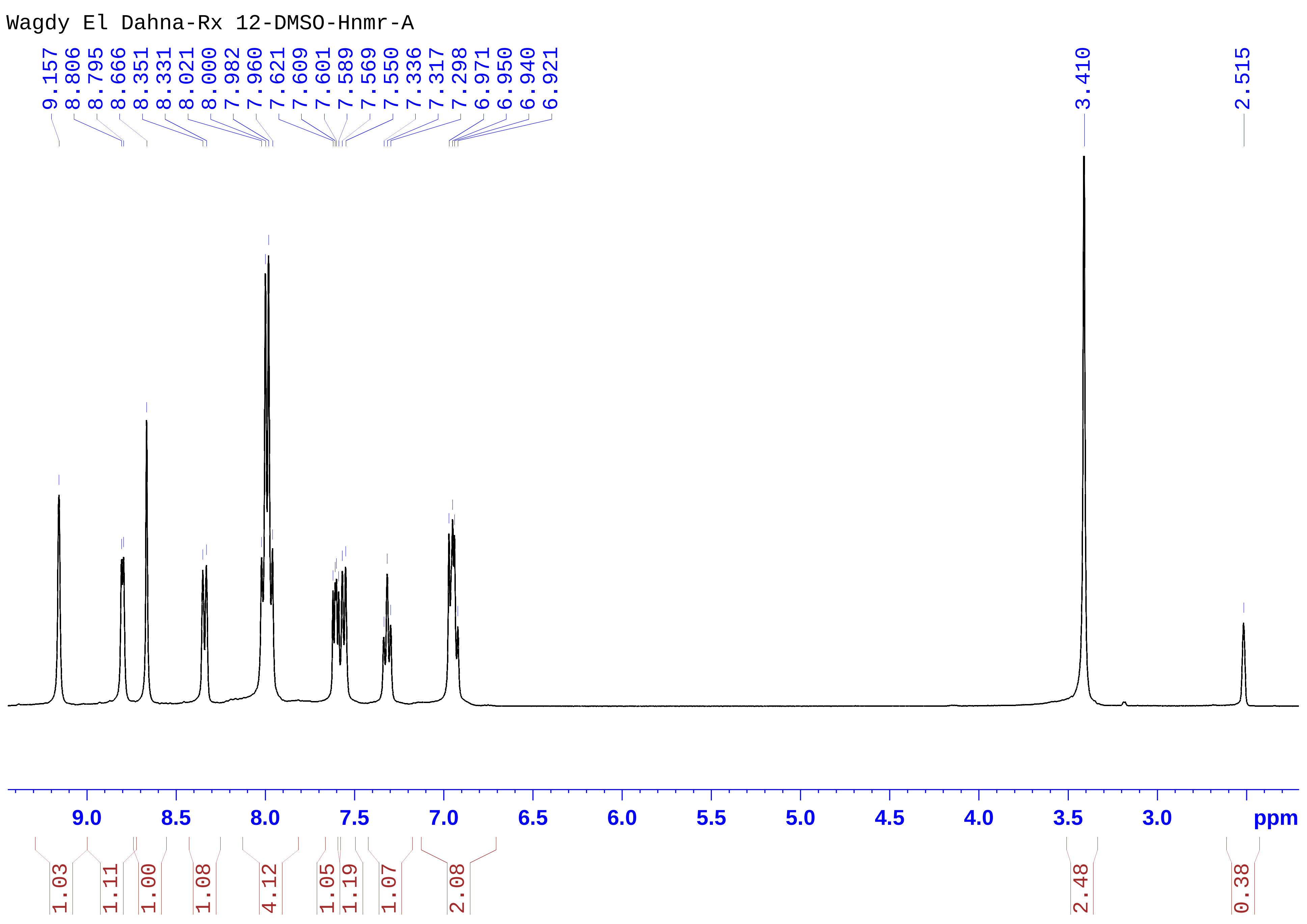
Wagdy El Dahna-Rx 12-DMSO-Hnmr-A
9.157
8.806
8.795
8.666
8.351
8.331
8.021
8.000
7.982
7.960
7.621
7.609
7.601
7.589
7.569
7.550
7.336
7.317
7.298
6.971
6.950
6.940
6.921
3.410
2.515
9.0
8.5
8.0
7.5
7.0
6.5
6.0
5.5
5.0
4.5
4.0
3.5
3.0
ppm
1.03
1.11
1.00
1.08
4.12
1.05
1.19
1.07
2.08
2.48
0.38

13C NMR of compound 7

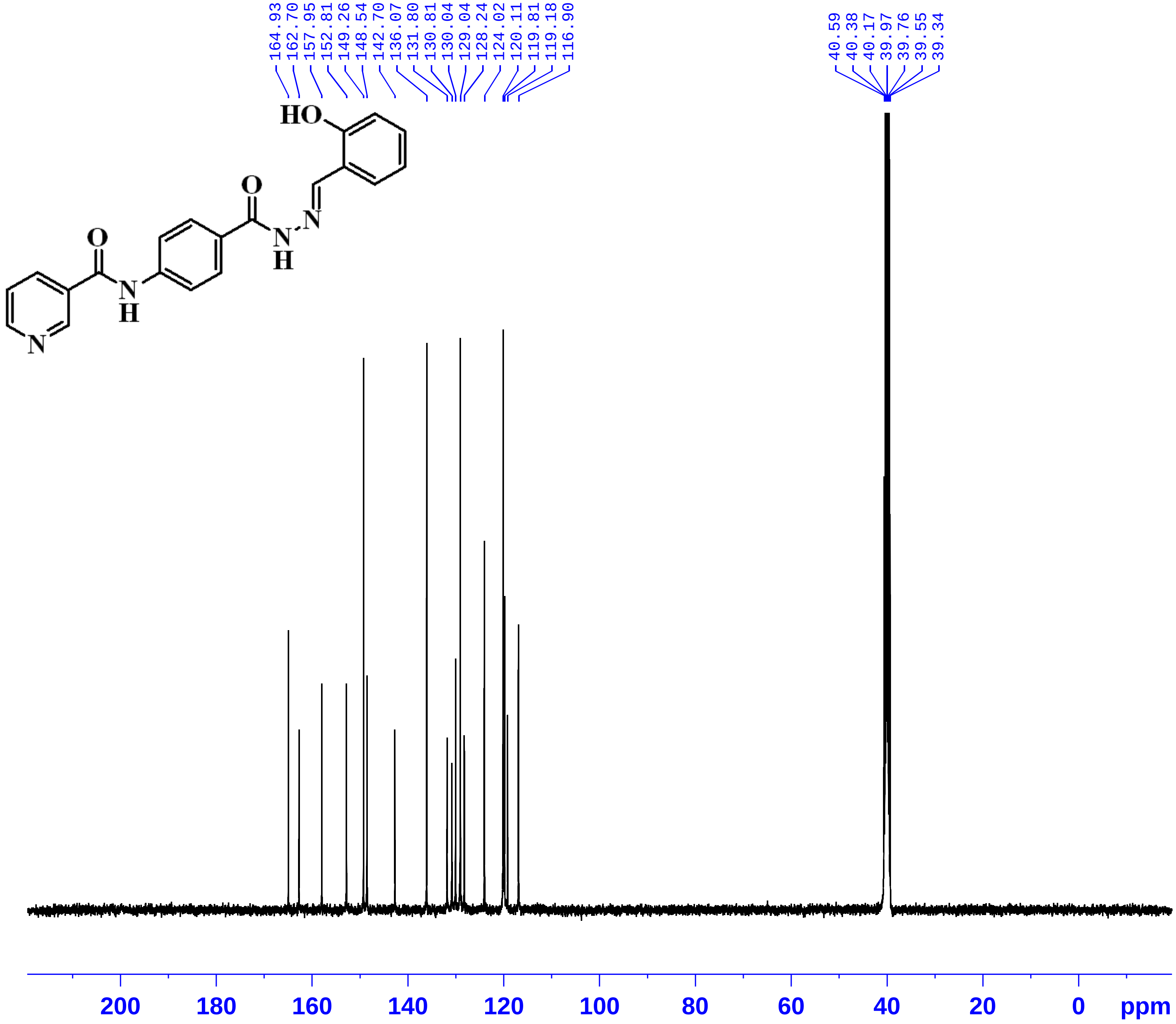

BRUKER

Faculty of Pharmacy

Current Data Parameters

NAME	Wagdy eldehna -RX12-carbon-Es
EXPNO	10
PROCNO	1

F2 - Acquisition Parameters

Date_	20210628
Time	2.26 h
INSTRUM	spect
PROBHD	Z108618_0945 (
PULPROG	zgpg30
TD	65536
SOLVENT	DMSO
NS	2200
DS	4
SWH	24038.461 Hz
FIDRES	0.733596 Hz
AQ	1.3631488 sec
RG	197.77
DW	20.800 usec
DE	6.50 usec
TE	297.3 K
D1	2.00000000 sec
D11	0.03000000 sec
TD0	1
SFO1	100.6404331 MHz
NUC1	13C
P1	10.00 usec
PLW1	47.00000000 W
SFO2	400.2016008 MHz
NUC2	1H
CPDPRG[2	waltz16
PCPD2	90.00 usec
PLW2	13.00000000 W
PLW12	0.29249999 W
PLW13	0.14713000 W

F2 - Processing parameters

SI	32768
SF	100.6303700 MHz
WDW	EM
SSB	0
LB	1.00 Hz
GB	0
PC	1.40

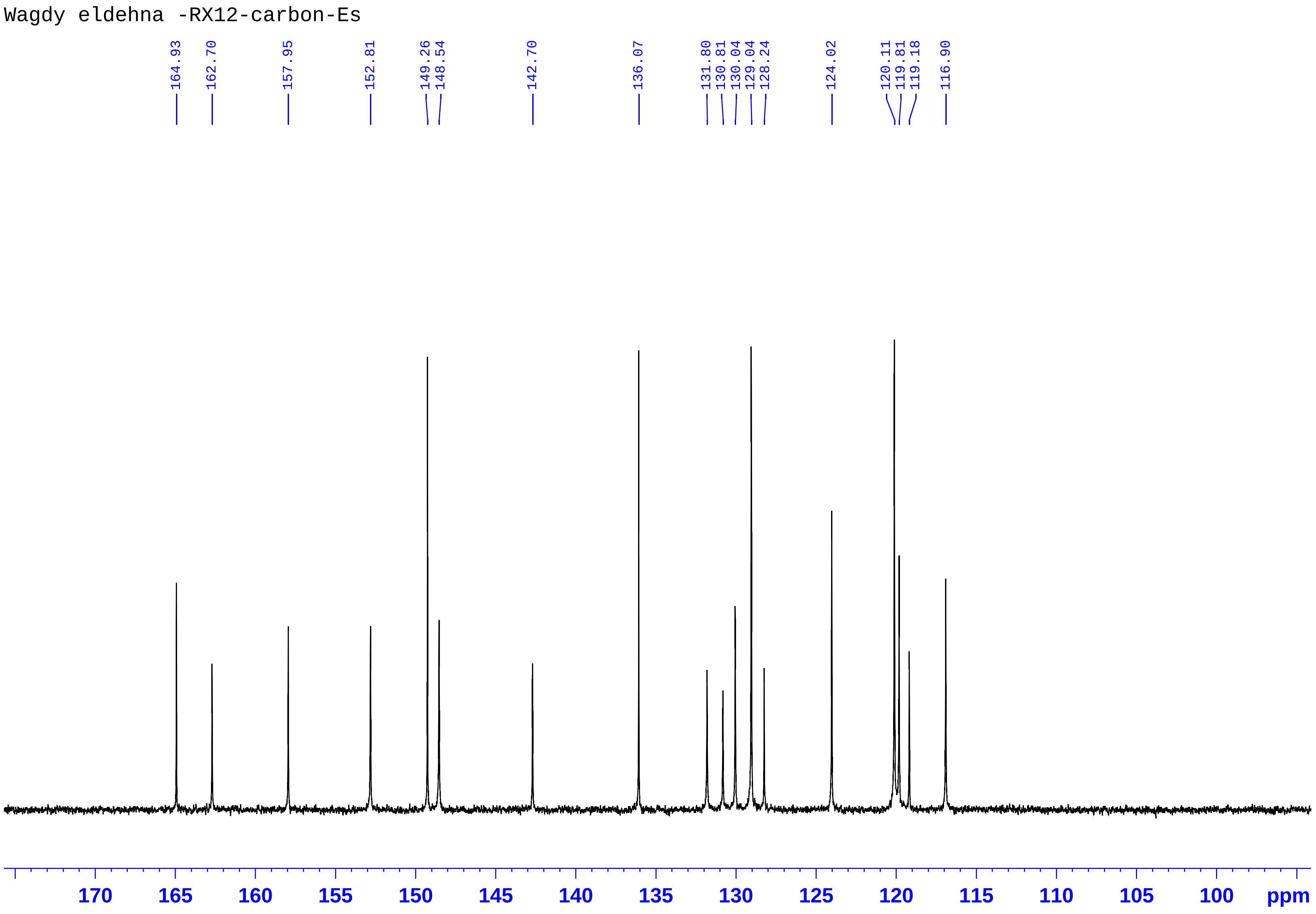

Wagdy eldehna -RX12-carbon-Es
164.93
162.70
157.95
152.81
149.26
148.54
142.70
136.07
131.80
130.81
130.04
129.04
128.24
124.02
120.11
119.81
119.18
116.90
170
165
160
155
150
145
140
135
130
125
120
115
110
105
100
ppm

13C NMR of compound 8

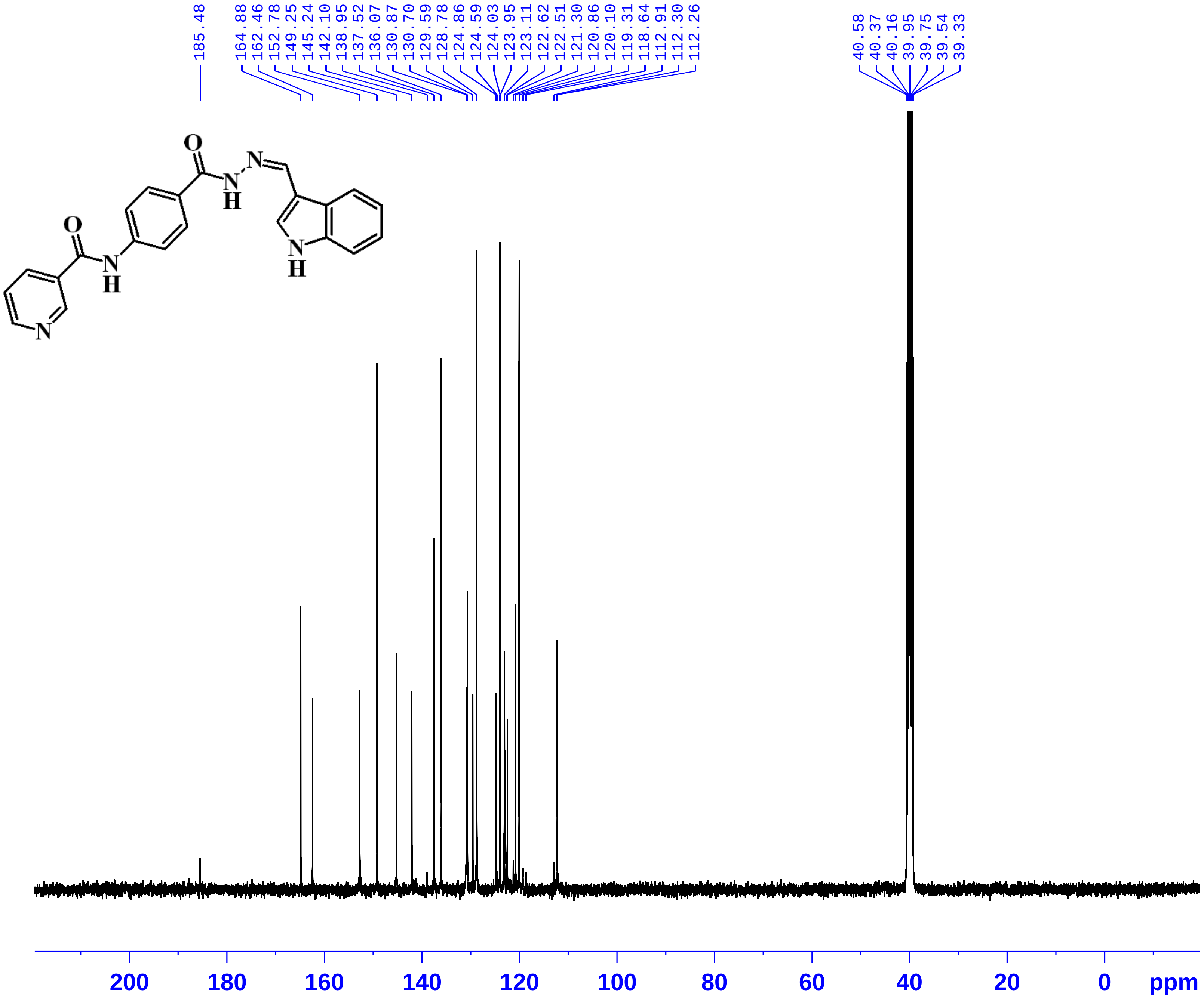

Current Data Parameters

NAME	Wagdy eldehna -Rx-17-carbon-Es
EXPNO	40
PROCNO	1

F2 - Acquisition Parameters

Date_	20210627
Time	17.49 h
INSTRUM	spect
PROBHD	Z108618_0945 (
PULPROG	zgpg30
TD	65536
SOLVENT	DMSO
NS	2200
DS	4
SWH	24038.461 Hz
FIDRES	0.733596 Hz
AQ	1.3631488 sec
RG	197.77
DW	20.800 usec
DE	6.50 usec
TE	297.8 K
D1	2.00000000 sec
D11	0.03000000 sec
TD0	1
SFO1	100.6404331 MHz
NUC1	13C
P1	10.00 usec
PLW1	47.00000000 W
SFO2	400.2016008 MHz
NUC2	1H
CPDPRG[2	waltz16
PCPD2	90.00 usec
PLW2	13.00000000 W
PLW12	0.29249999 W
PLW13	0.14713000 W

F2 - Processing parameters

SI	32768
SF	100.6303700 MHz
WDW	EM
SSB	0
LB	1.00 Hz
GB	0
PC	1.40

Wagdy eldehna -Rx-17-carbon-Es

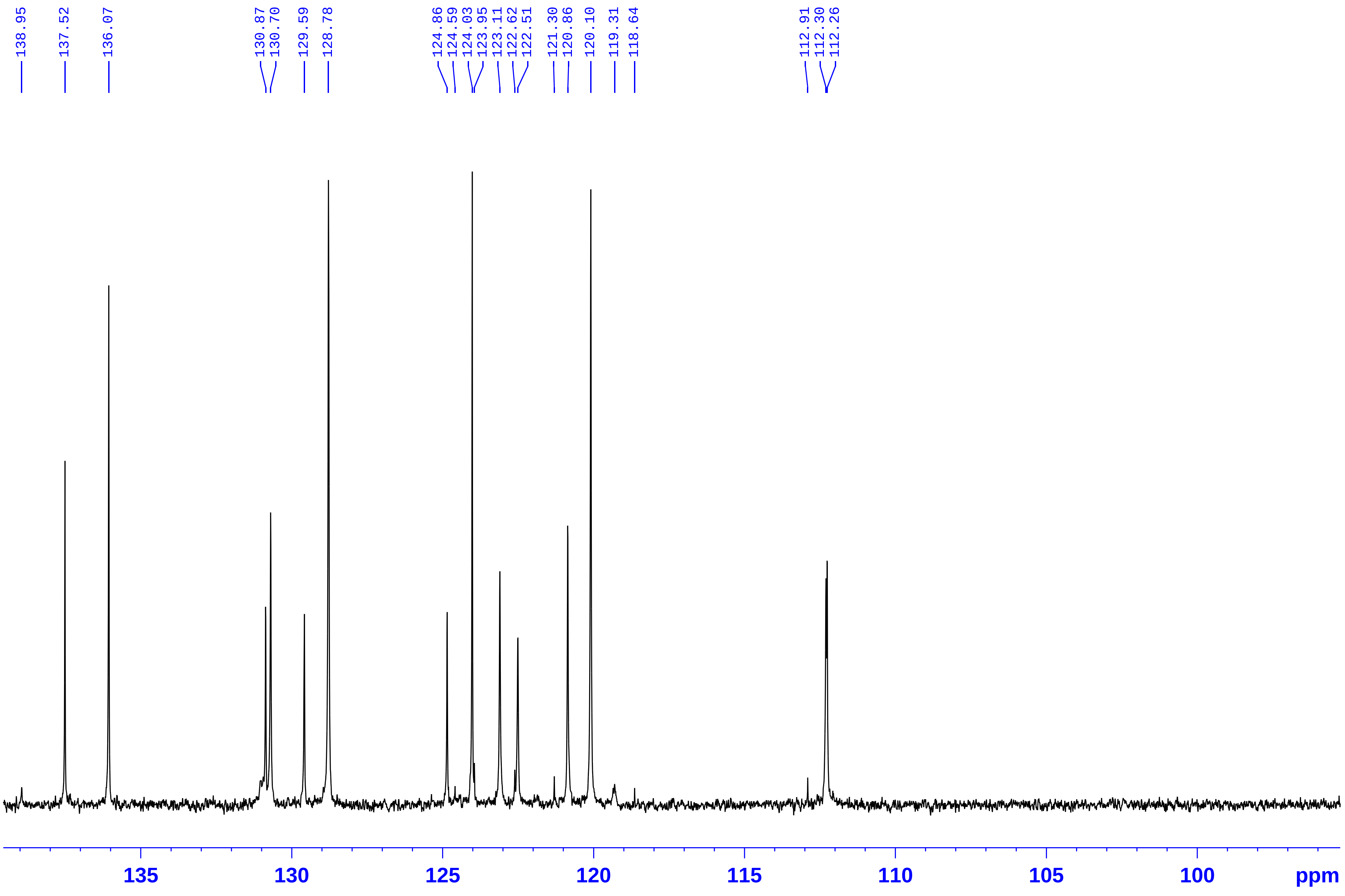

13C NMR of compound 8

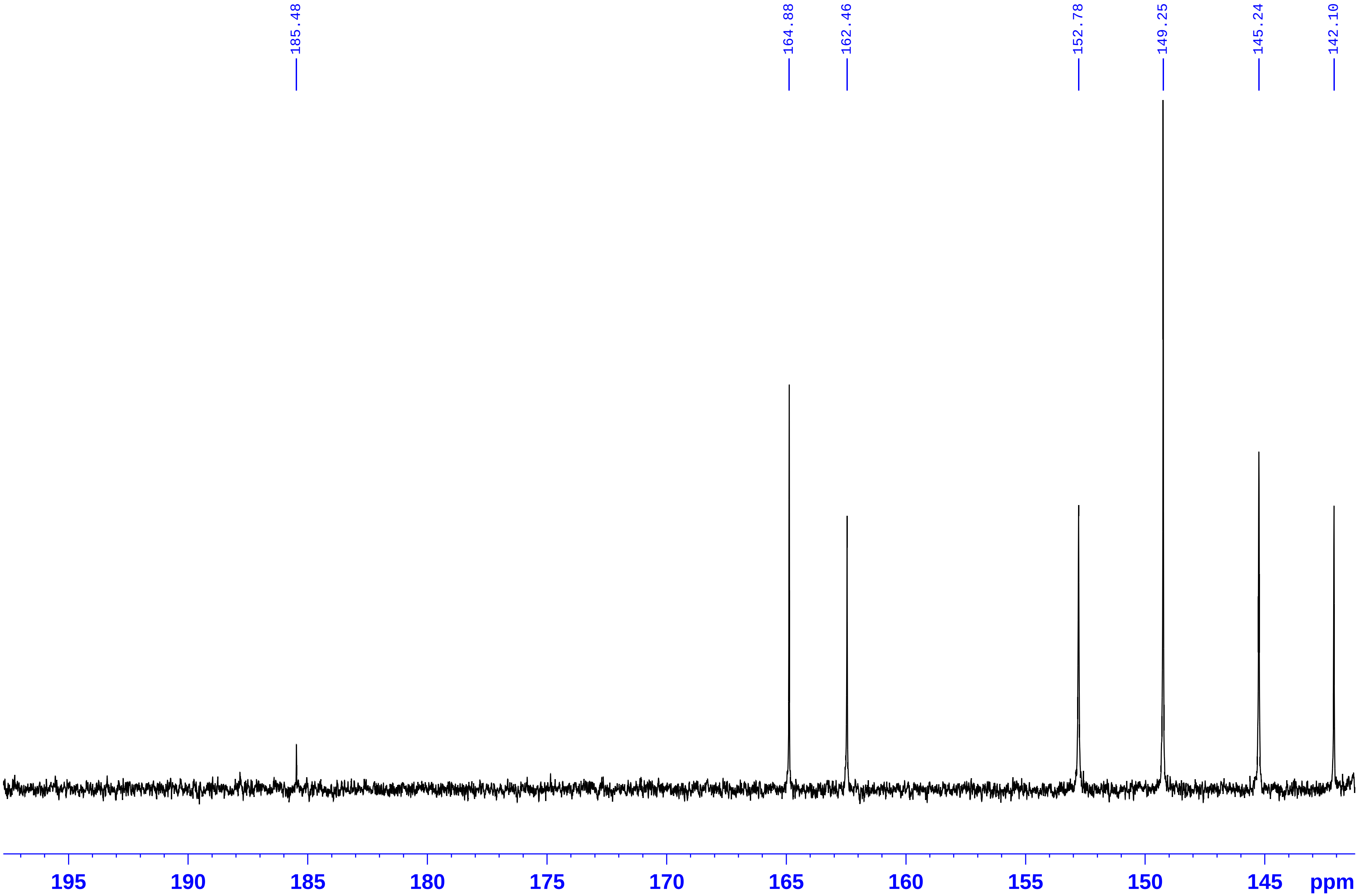

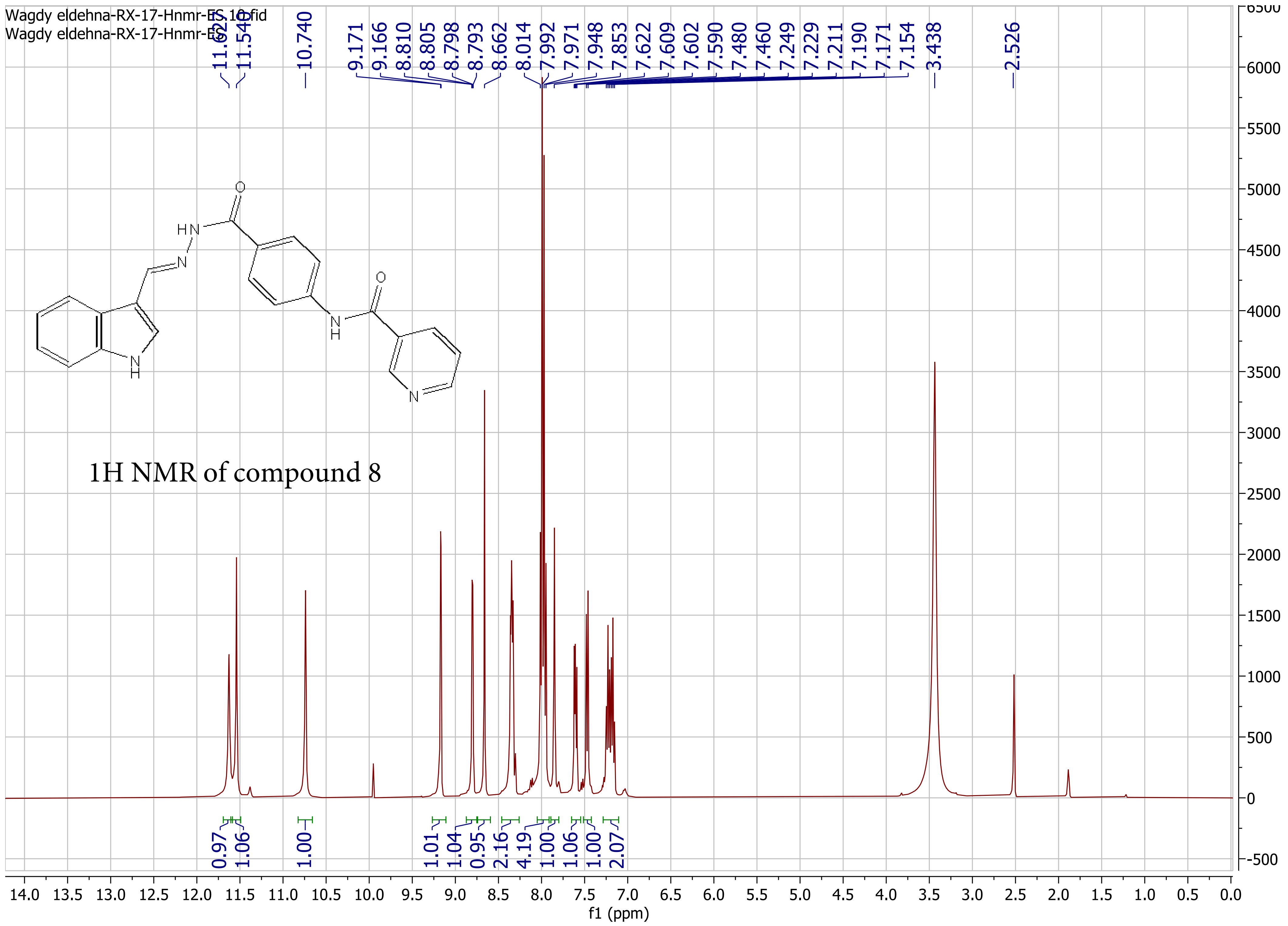
Wagdy eldehna-RX-17-Hnmr-ES.10.fid
Wagdy eldehna-RX-17-Hnmr-ES
11.540
10.740
9.171
9.166
8.810
8.805
8.798
8.793
8.662
8.014
7.992
7.971
7.948
7.853
7.622
7.609
7.602
7.590
7.480
7.460
7.249
7.229
7.211
7.190
7.171
7.154
3.438
2.526
HN
N
O
O
N
H
N
H
N
1H NMR of compound 8
0.97
1.06
1.00
1.01
1.04
0.95
2.16
4.19
1.00
1.06
1.00
2.07
f1 (ppm)

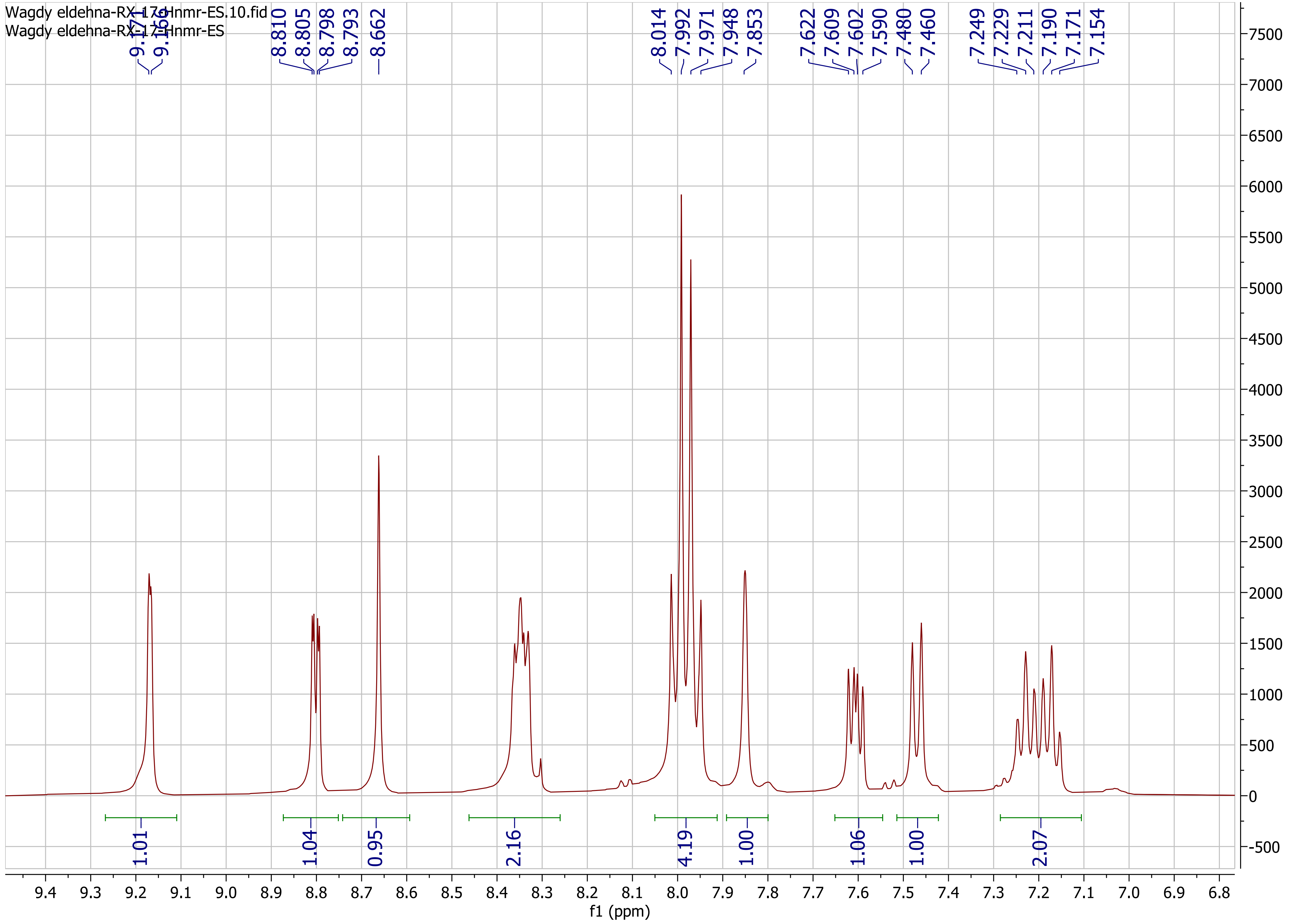

Wagdy eldehna-RX-17-Hnmr-ES.10.fid
Wagdy eldehna-RX-17-Hnmr-ES
9.171
8.810
8.805
8.798
8.793
8.662
8.014
7.992
7.971
7.948
7.853
7.622
7.609
7.602
7.590
7.480
7.460
7.249
7.229
7.211
7.190
7.171
7.154
1.01
1.04
0.95
2.16
4.19
1.00
1.06
1.00
2.07
9.4
9.3
9.2
9.1
9.0
8.9
8.8
8.7
8.6
8.5
8.4
8.3
8.2
8.1
8.0
7.9
7.8
7.7
7.6
7.5
7.4
7.3
7.2
7.1
7.0
6.9
6.8
f1 (ppm)
7500
7000
6500
6000
5500
5000
4500
4000
3500
3000
2500
2000
1500
1000
500
0
-500

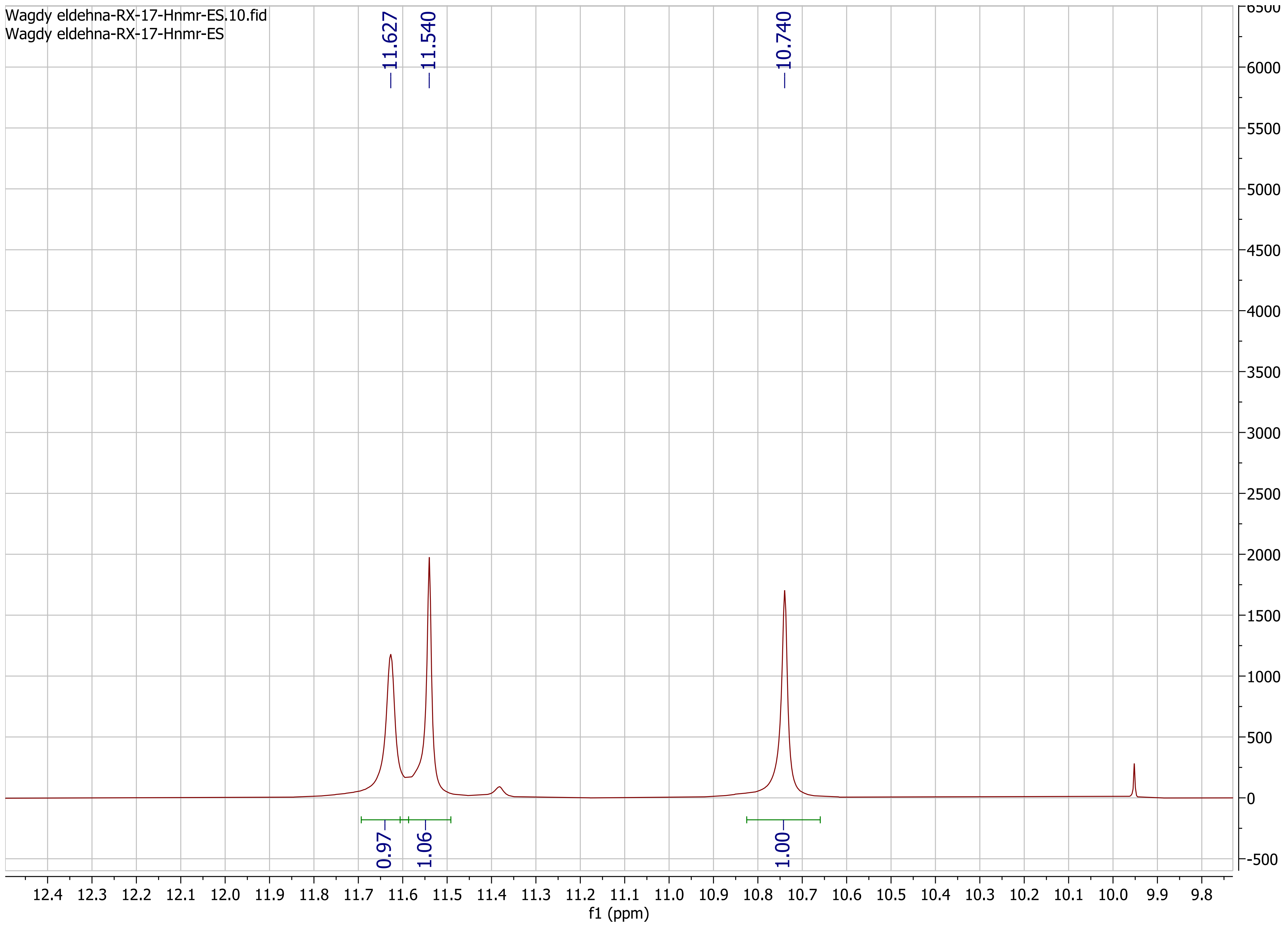
Wagdy eldehna-RX-17-Hnmr-ES.10.fid
Wagdy eldehna-RX-17-Hnmr-ES
11.627
11.540
10.740
0.97
1.06
1.00
f1 (ppm)

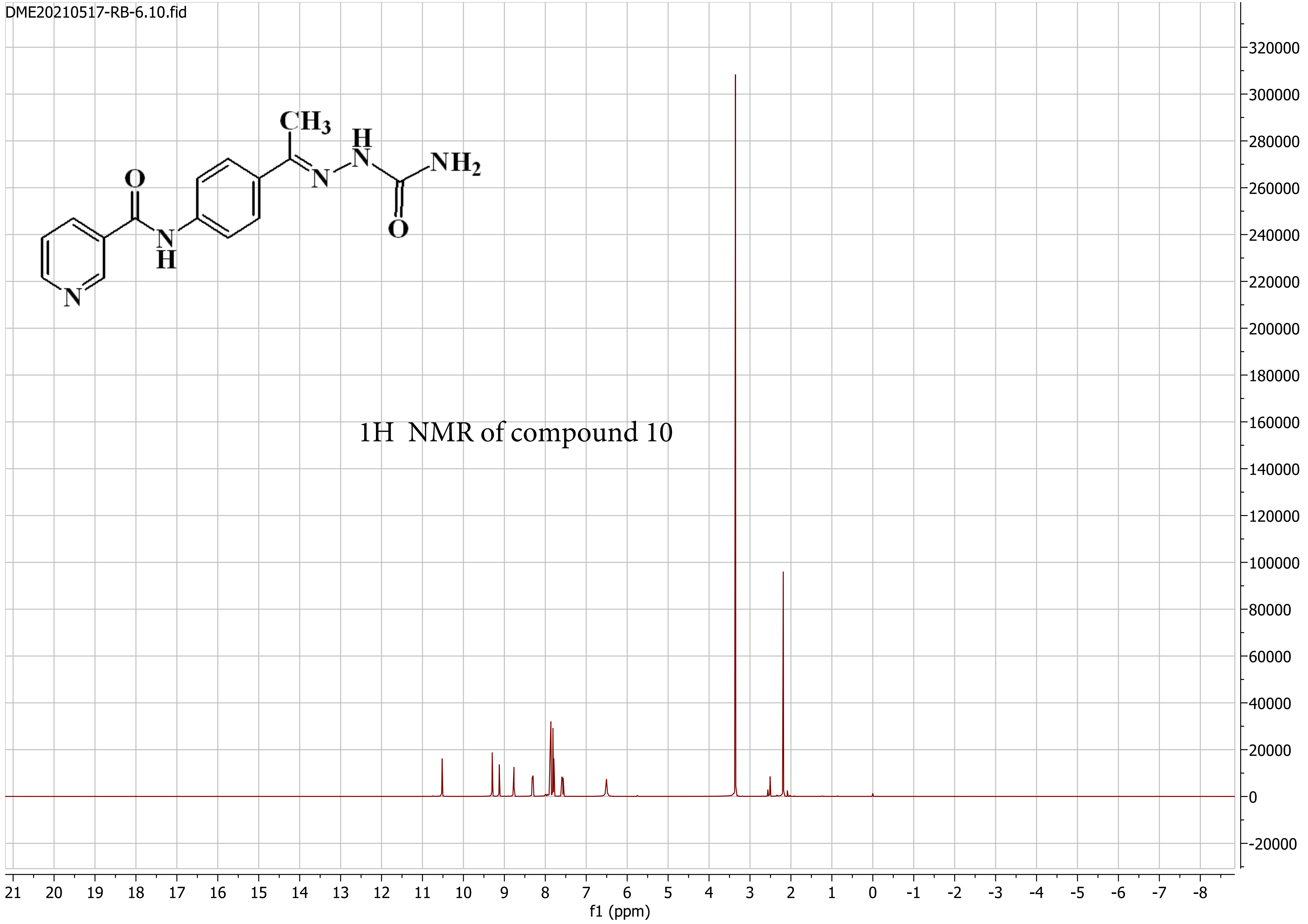

1H NMR of compound 10

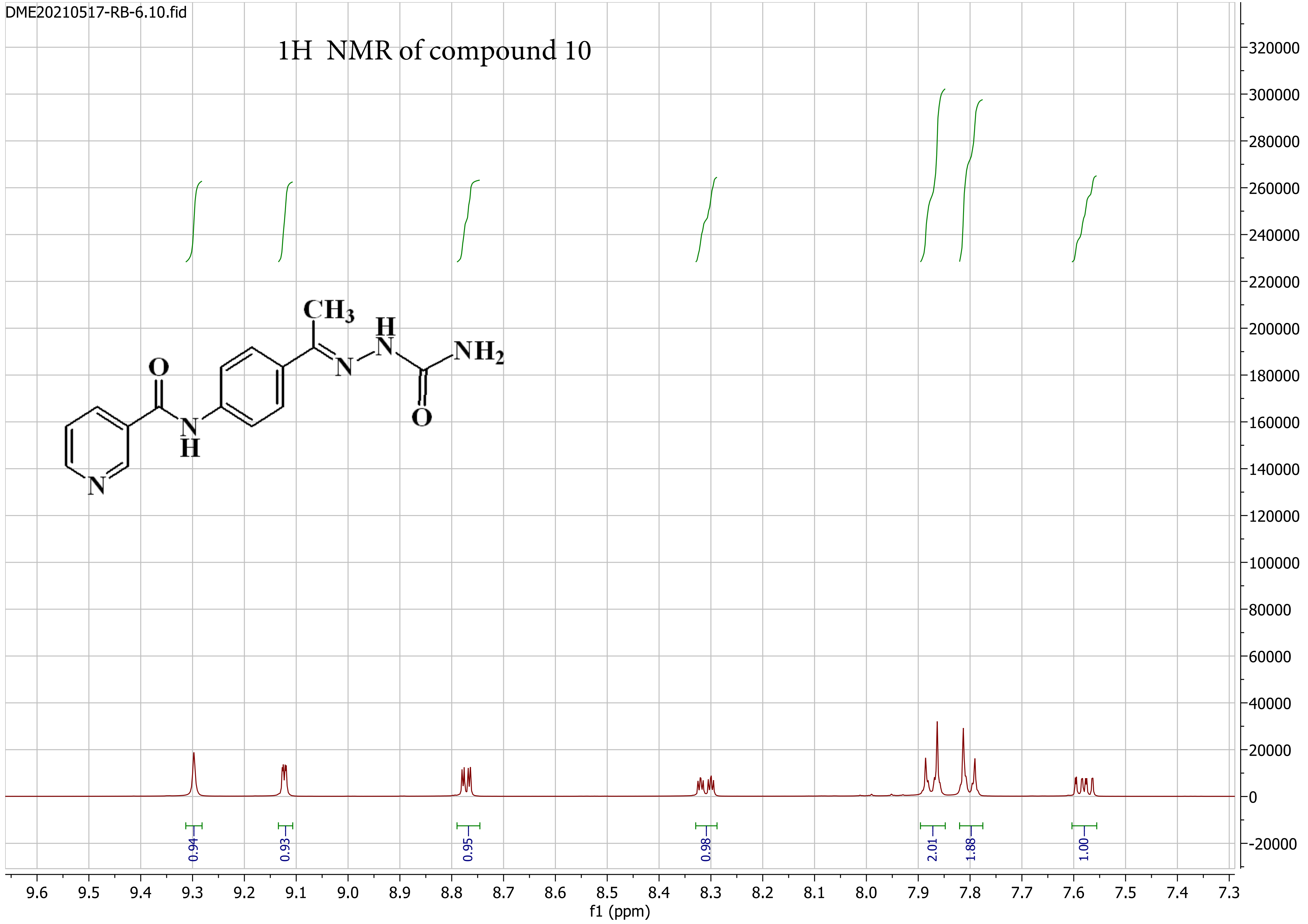
DME20210517-RB-6.10.fid
1H NMR of compound 10
CH3
H
N
NH2
O
N
H
N
O
N
0.94
0.93
0.95
0.98
2.01
1.88
1.00
9.6 9.5 9.4 9.3 9.2 9.1 9.0 8.9 8.8 8.7 8.6 8.5 8.4 8.3 8.2 8.1 8.0 7.9 7.8 7.7 7.6 7.5 7.4 7.3
f1 (ppm)
320000 300000 280000 260000 240000 220000 200000 180000 160000 140000 120000 100000 80000 60000 40000 20000 0 -20000

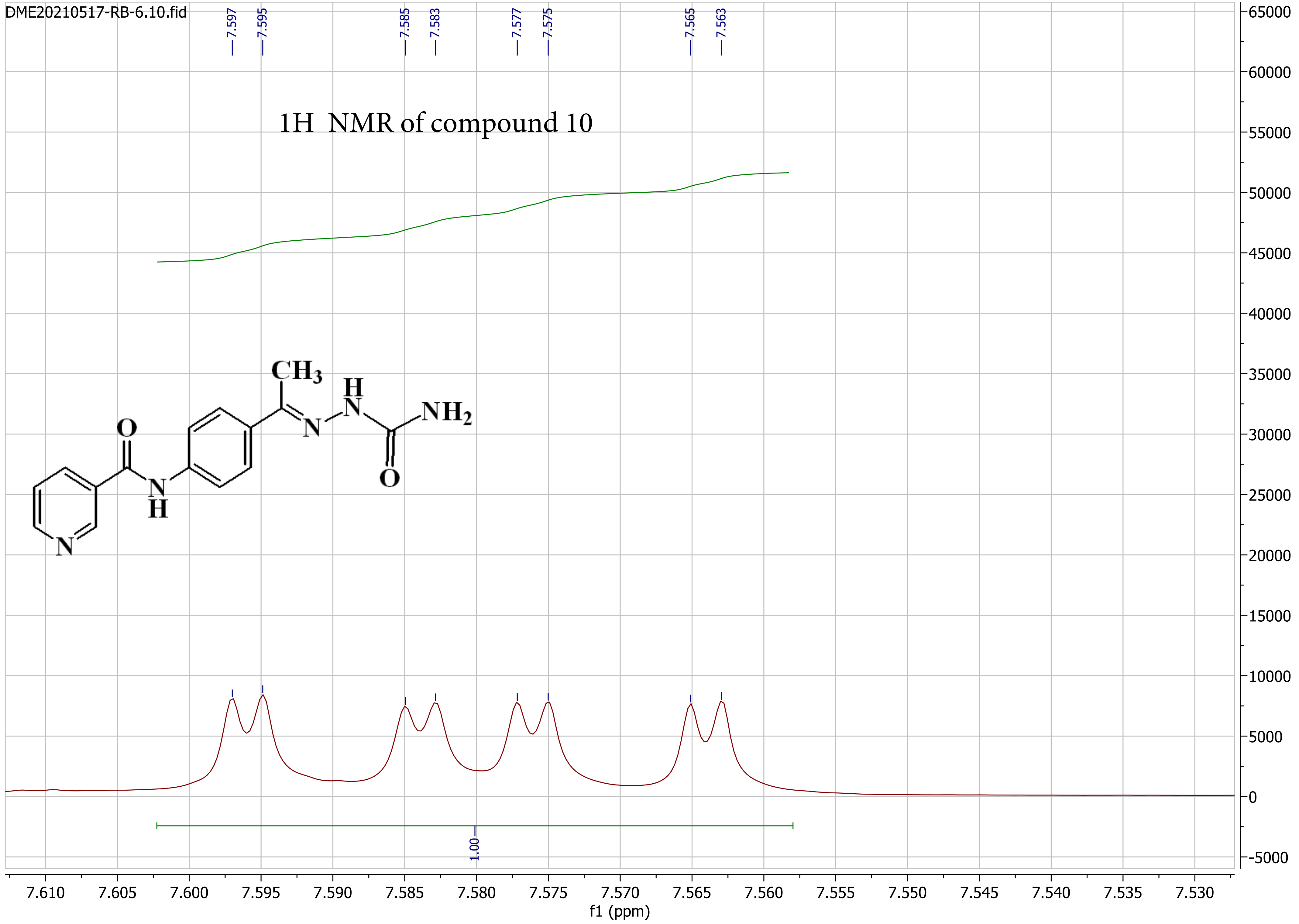
DME20210517-RB-6.10.fid
7.597
7.595
7.585
7.583
7.577
7.575
7.565
7.563
1H NMR of compound 10
CH3
H
N
NH2
O
N
O
N
H
N
1.00
f1 (ppm)

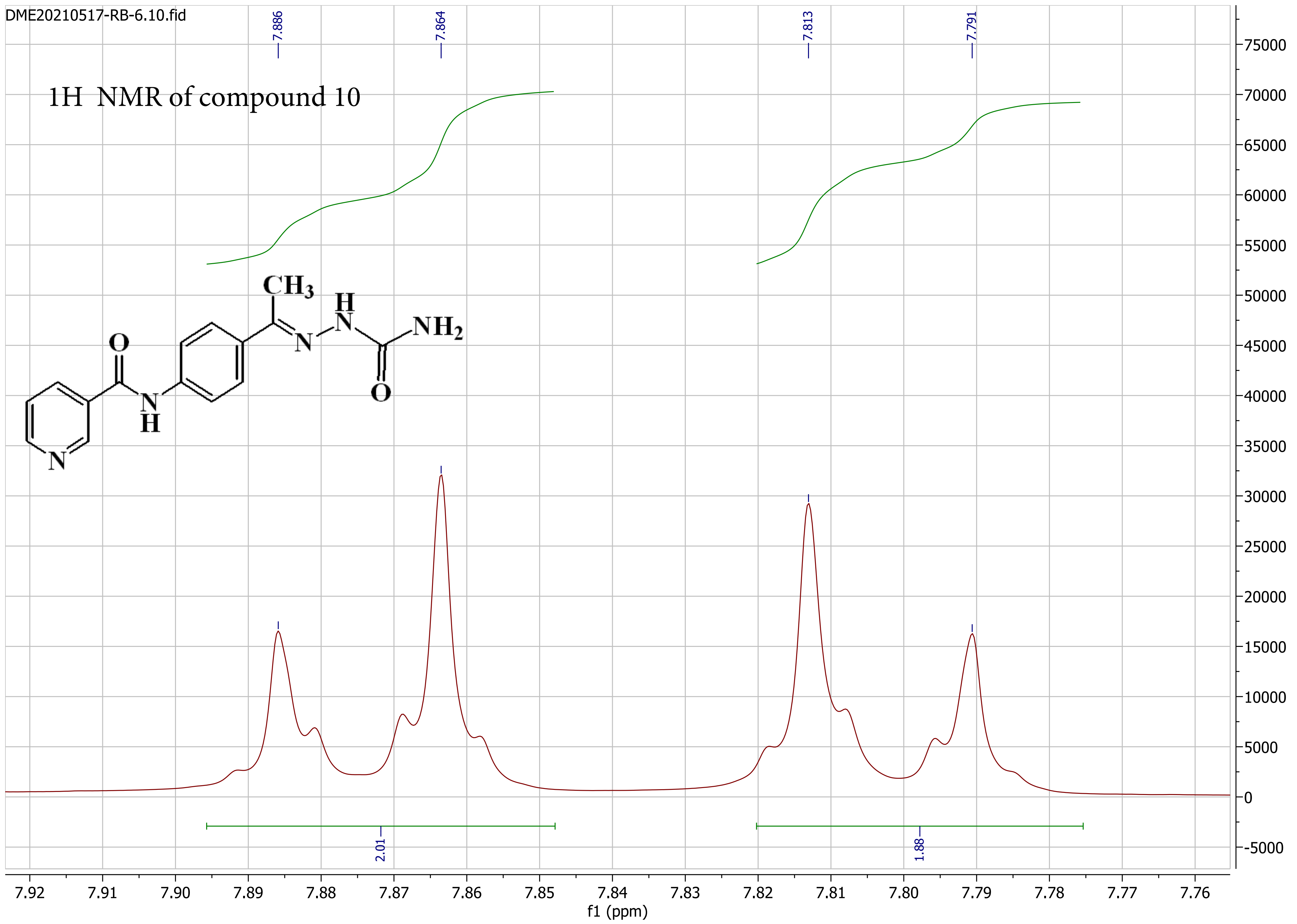
DME20210517-RB-6.10.fid
1H NMR of compound 10
7.886
7.864
7.813
7.791
2.01
1.88
f1 (ppm)

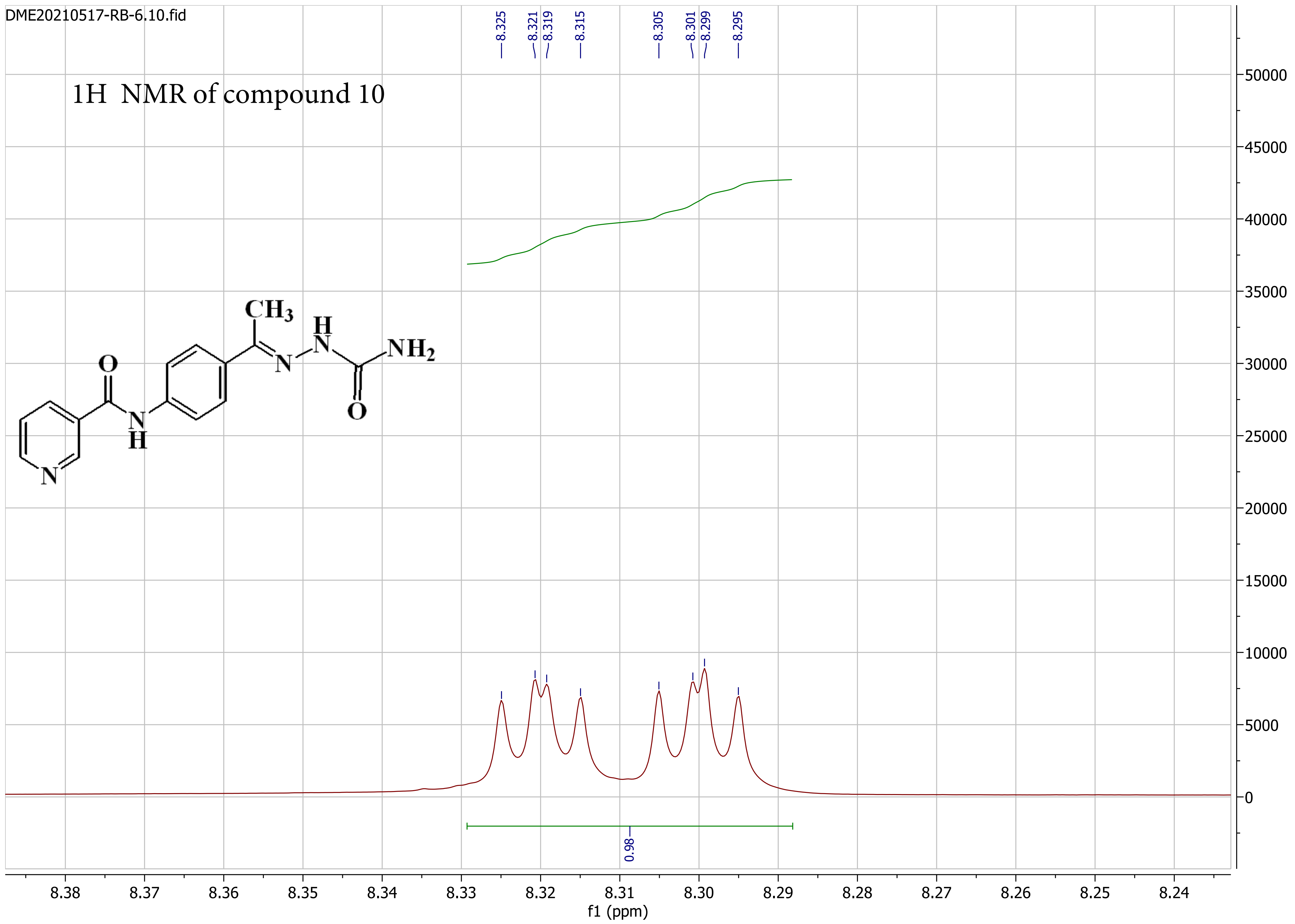
DME20210517-RB-6.10.fid
1H NMR of compound 10
8.325
8.321
8.319
8.315
8.305
8.301
8.299
8.295
0.98
8.38
8.37
8.36
8.35
8.34
8.33
8.32
8.31
8.30
8.29
8.28
8.27
8.26
8.25
8.24
f1 (ppm)
50000
45000
40000
35000
30000
25000
20000
15000
10000
5000
0
CH3
H
N
NH2
O
N
O
N
H
N

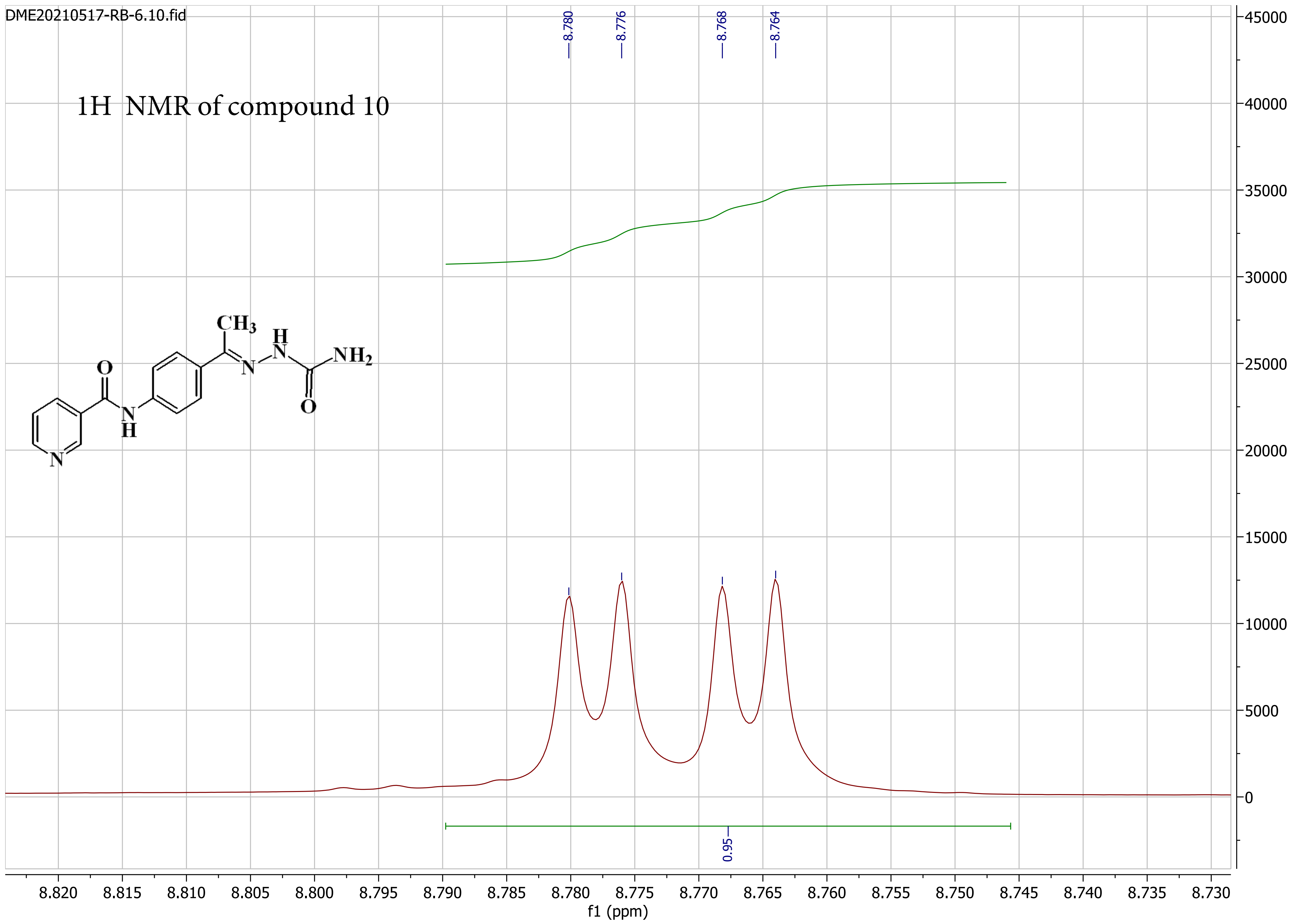
DME20210517-RB-6.10.fid
1H NMR of compound 10
8.780
8.776
8.768
8.764
0.95
f1 (ppm)

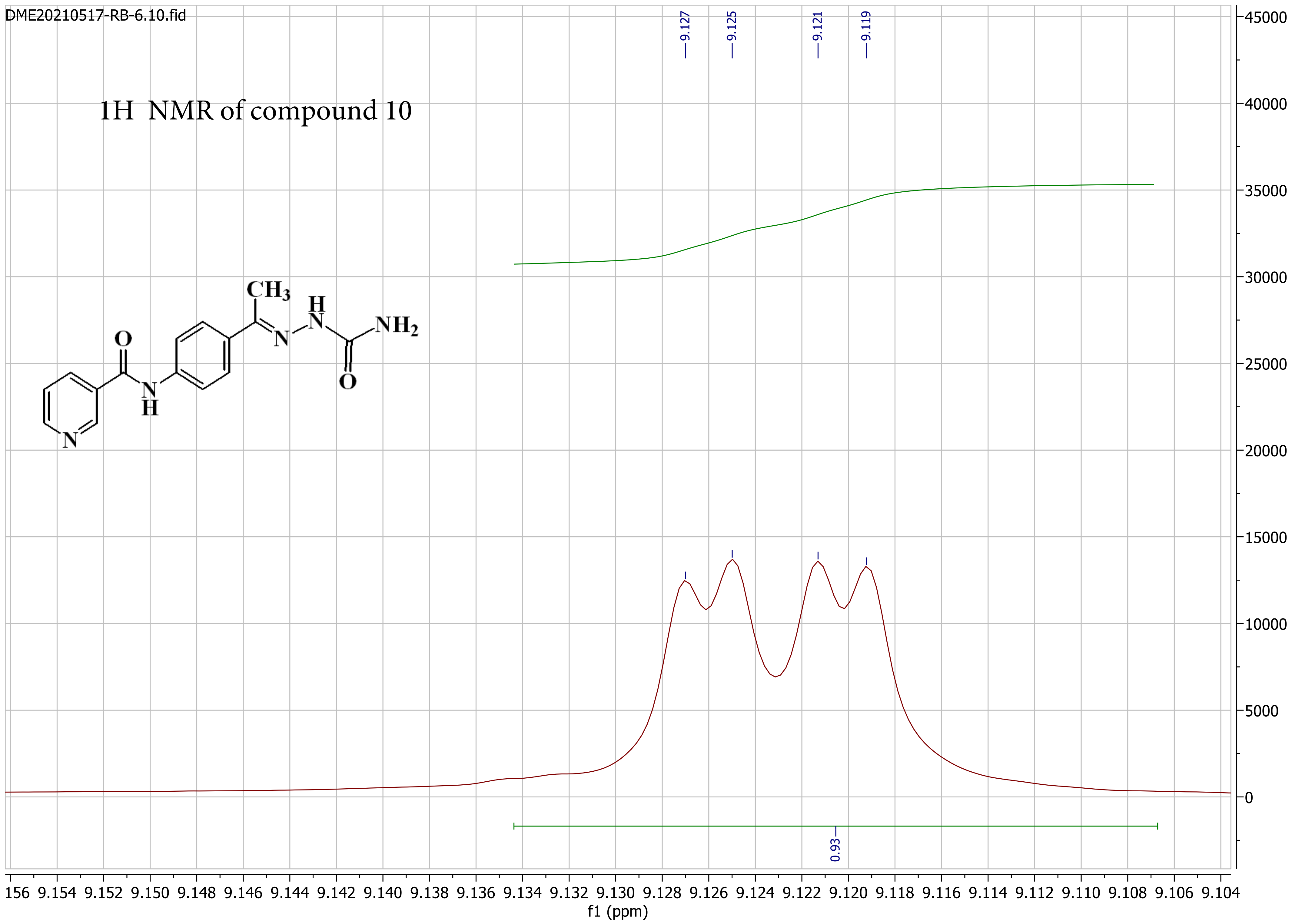
DME20210517-RB-6.10.fid
1H NMR of compound 10
9.127
9.125
9.121
9.119
0.93
f1 (ppm)

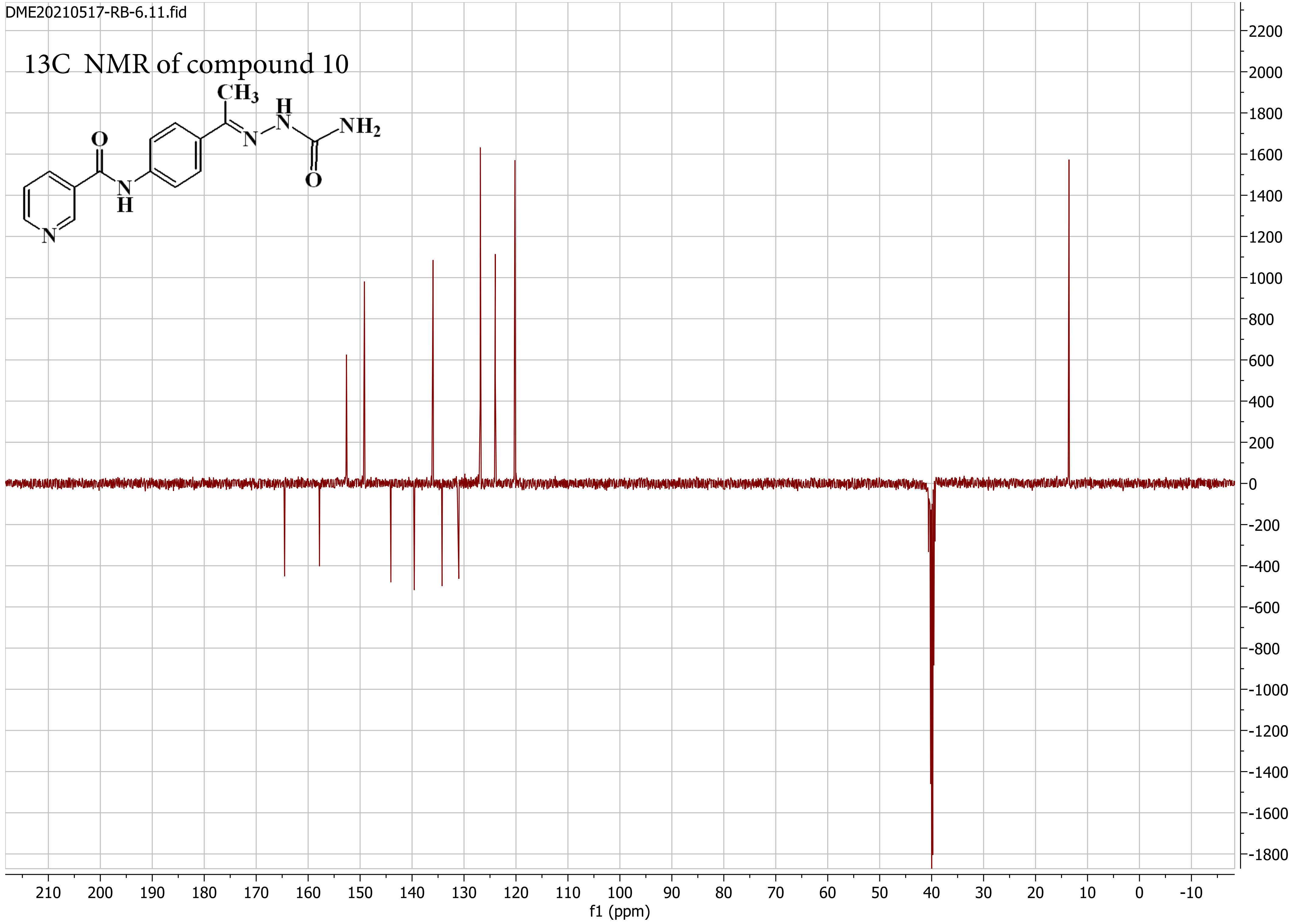

DME20210517-RB-6.11.fid
13C NMR of compound 10
CH3
H
N
NH2
O
N
N
H
O
N
f1 (ppm)

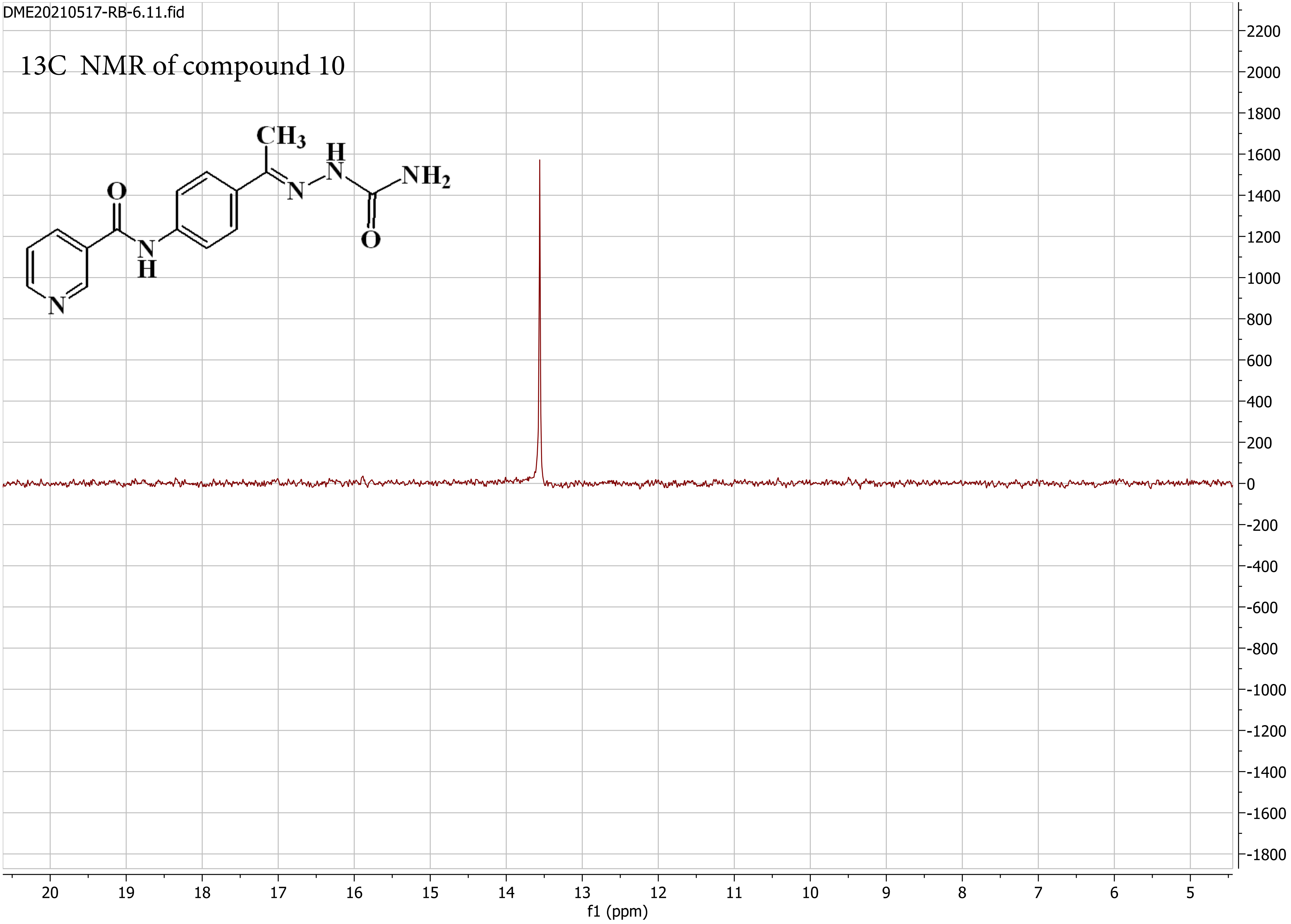
DME20210517-RB-6.11.fid
13C NMR of compound 10
CH3
H
N
NH2
O
N
H
N
O
N
f1 (ppm)

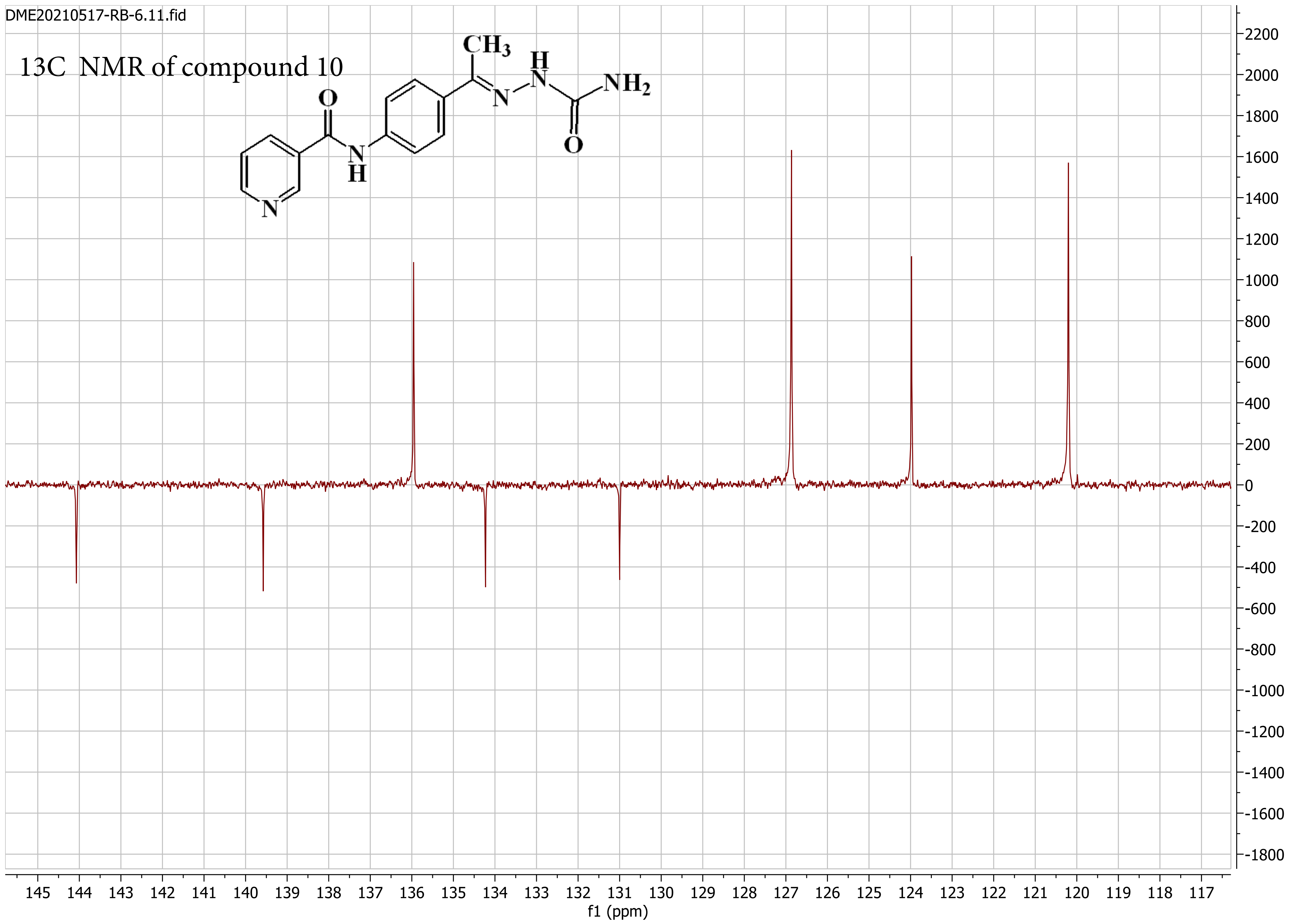
DME20210517-RB-6.11.fid
13C NMR of compound 10
f1 (ppm)

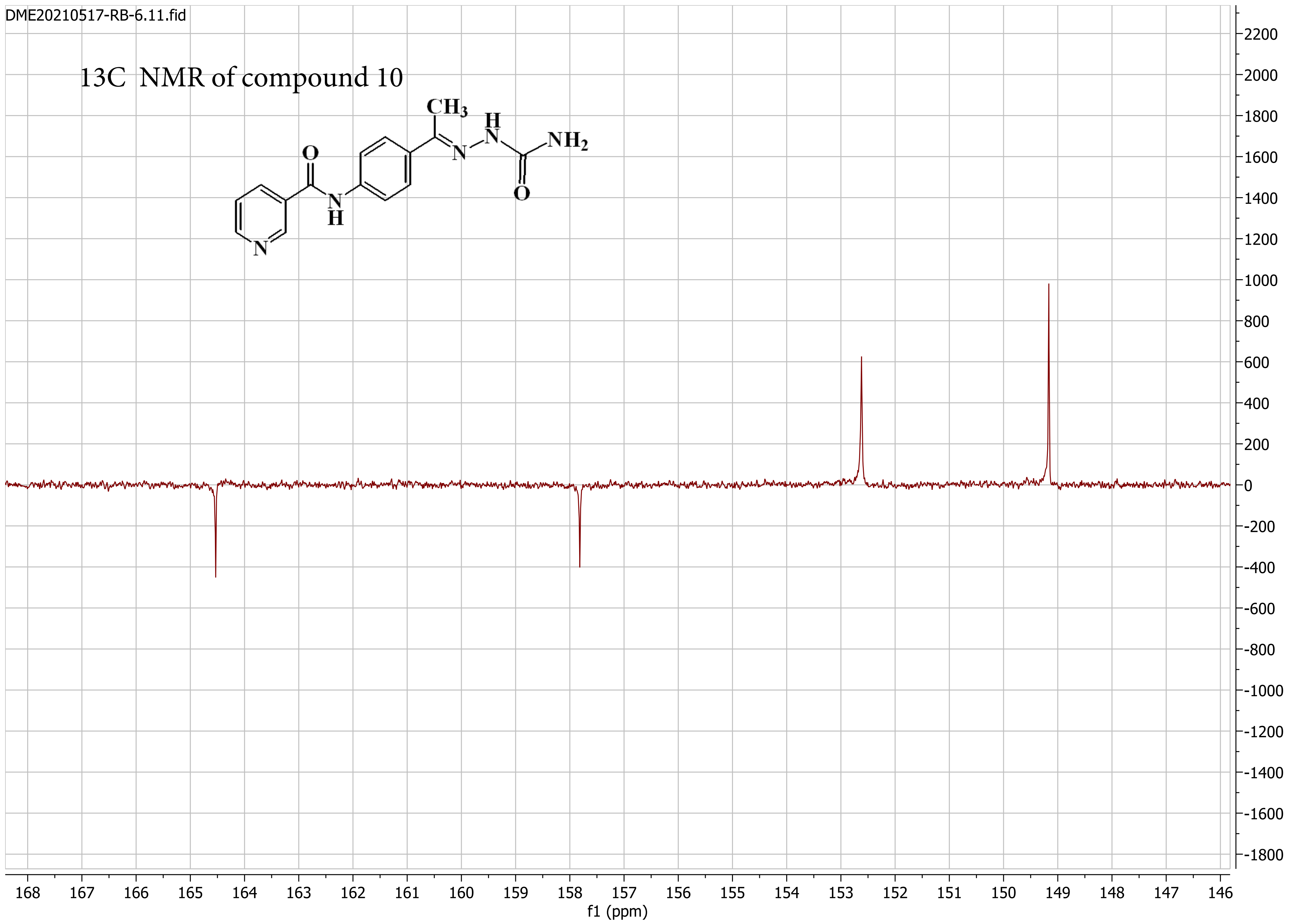
DME20210517-RB-6.11.fid
13C NMR of compound 10
CH3
H
N
NH2
O
N
N
H
O
N
f1 (ppm)

Al-Azhar University
The Regional Center for Mycology and Biotechnology

Requester Data:

Name: **Dr. Ibrahim Hasan Eisa**

Authority: Faculty of Pharmacy, Al-Azhar University

Sample Data:

Four samples had been submitted for elemental analysis.

Analysis Report:

Sample Number	Sample Code	C%	H%	N%
6	X9	63.69	4.15	15.08
7	X12	66.90	4.67	15.69
8	X17	68.74	4.58	18.48
10	RB6	60.87	5.31	23.72

INVESTIGATOR **DIRECTOR**

جامعة الأزهر
المركز الإقليمي للفطريات وتطبيقاتها

Al-Azhar University Campus - Nasr City, Cairo, Egypt.
Tel: 0202 22620373 Fax : 0202 22620373
E.mail:rcmb@azhar.edu.eg
Website: http://www.azhar.edu.eg.htm * http://www.azhar.edu.eg/pages/fungi_center.htm
Facebook : RCMB AZHAR P.O. box mail : 11751 Nasr City Cairo, Egypt.

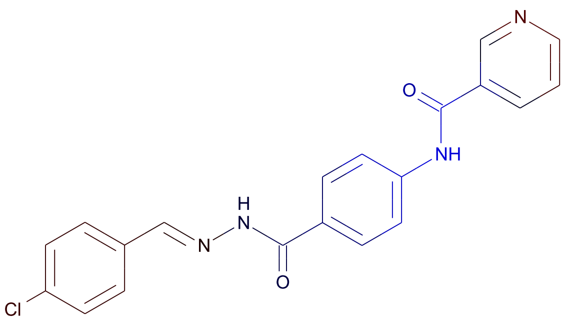

$C_{20}H_{15}ClN_4O_2$
Molecular Weight: 378.8117
ALogP: 2.981
Rotatable Bonds: 5
Acceptors: 4
Donors: 2

Model Prediction

Prediction: Mild
Probability: 0.717
Enrichment: 1.04
Bayesian Score: -3.21
Mahalanobis Distance: 6.04
Mahalanobis Distance p-value: 1

Prediction: Positive if the Bayesian score is above the estimated best cutoff value from minimizing the false positive and false negative rate.
Probability: The esimated probability that the sample is in the positive category. This assumes that the Bayesian score follows a normal distribution and is different from the prediction using a cutoff.
Enrichment: An estimate of enrichment, that is, the increased likelihood (versus random) of this sample being in the category.
Bayesian Score: The standard Laplacian-modified Bayesian score.
Mahalanobis Distance: The Mahalanobis distance (MD) is the distance to the center of the training data. The larger the MD, the less trustworthy the prediction.
Mahalanobis Distance p-value: The p-value gives the fraction of training data with an MD greater than or equal to the one for the given sample, assuming normally distributed data. The smaller the p-value, the less trustworthy the prediciton. For highly non-normal X properties (e.g., fingerprints), the MD p-value is wildly inaccurate.

Structural Similar Compounds

Name	1-AMINO-4-BENZOYLAMINO-ANTHRAQUINONE	5-NORBORNENE-2;3-DICARBOXYLIC ACID; 1;4;5;6;7;7-HEXACHLORO-	ANTHRAQUINONE; 1-AMINO-4-HYDROXY-2-PHENOXY-
Structure	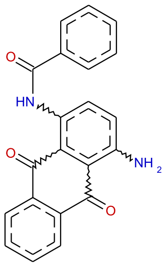	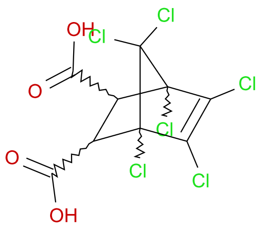	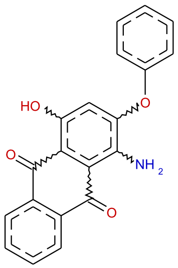
Actual Endpoint	Mild	Moderate_Severe	Mild
Predicted Endpoint	Mild	Moderate_Severe	Mild
Distance	0.564	0.587	0.646
Reference	28ZPAK-;124;72	28ZPAK-;92;72	28ZPAK 239;72

Model Applicability

Unknown features are fingerprint features in the query molecule, but not found or appearing too infrequently in the training set.

1. All properties and OPS components are within expected ranges.
2. Unknown FCFP_2 feature: 581019816: [*]N\N=C\[*]

Feature Contribution

Top features for positive contribution				
**Fingerprint**	**Bit/Smiles**	**Feature Structure**	**Score**	**Moderate_Severe in training set**
FCFP_10	-149636017	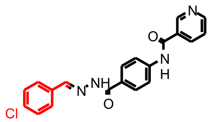 [*]=C[c]1:[cH]:[cH]:[c](Cl):[cH]:[cH]:1	0.352	7 out of 7

FCFP_10	-1508180856	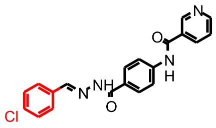 [*][c]1:[cH]:[cH]:[c](Cl):[cH]:[cH]:1	0.329	16 out of 17
FCFP_10	547884906	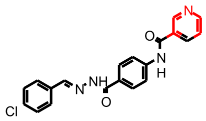 [*][c]1:[*]:[cH]:[cH]:n:[cH]:1	0.317	4 out of 4
**Top Features for negative contribution**				
**Fingerprint**	**Bit/Smiles**	**Feature Structure**	**Score**	**Moderate_Severe in training set**
FCFP_10	-581879738	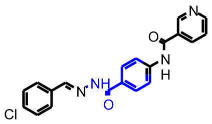 [*]NC(=O)[c]1:[cH]:[cH]:[*]:[cH]:[cH]:1	-1.29	0 out of 4
FCFP_10	1175232969	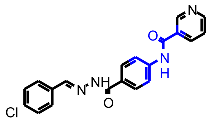 [*]:[cH]:[c](NC(=O)[c](:[*]):[*]):[cH]:[*]	-1.29	0 out of 4
FCFP_10	-1925475824	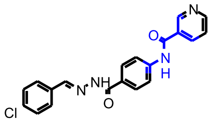 [*]:[cH]:[c](:[cH]:[*])C(=O)N[c](:[*]):[*]	-1.29	0 out of 4

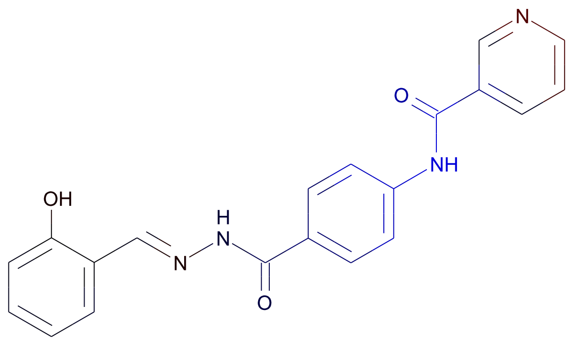

$C_{20}H_{16}N_4O_3$
Molecular Weight: 360.36604
ALogP: 2.075
Rotatable Bonds: 5
Acceptors: 5
Donors: 3

Structural Similar Compounds

Name	ANTHRAQUINONE; 1-((2-HYDROXYETHYL)AMINO)-4-(METHYLAMINO)-	2-NAPHTHALENESULFONIC ACID; 4-HYDROXY-7-(METHYLAMINO)-	p-Acetophenetidide; 3'-(bis(2-hydroxyethyl)amino)-
Structure	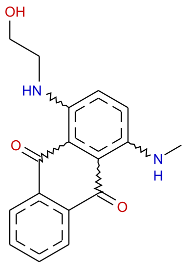	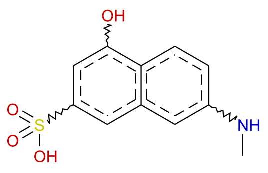	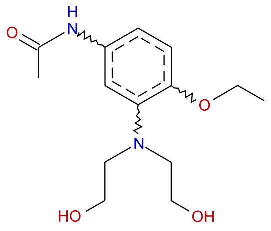
Actual Endpoint	Mild	Mild	Moderate_Severe
Predicted Endpoint	Mild	Mild	Moderate_Severe
Distance	0.563	0.698	0.699
Reference	28ZPAK 245;72	28ZPAK 190;72	Prehled Prumyslove Toxikologie; Organicke Latky; Marhold; J. -;645;86

Model Prediction

Prediction: Mild
Probability: 0.674
Enrichment: 0.978
Bayesian Score: -3.96
Mahalanobis Distance: 6.34
Mahalanobis Distance p-value: 1

Prediction: Positive if the Bayesian score is above the estimated best cutoff value from minimizing the false positive and false negative rate.
Probability: The esimated probability that the sample is in the positive category. This assumes that the Bayesian score follows a normal distribution and is different from the prediction using a cutoff.
Enrichment: An estimate of enrichment, that is, the increased likelihood (versus random) of this sample being in the category.
Bayesian Score: The standard Laplacian-modified Bayesian score.
Mahalanobis Distance: The Mahalanobis distance (MD) is the distance to the center of the training data. The larger the MD, the less trustworthy the prediction.
Mahalanobis Distance p-value: The p-value gives the fraction of training data with an MD greater than or equal to the one for the given sample, assuming normally distributed data. The smaller the p-value, the less trustworthy the prediciton. For highly non-normal X properties (e.g., fingerprints), the MD p-value is wildly inaccurate.

Model Applicability

Unknown features are fingerprint features in the query molecule, but not found or appearing too infrequently in the training set.

1. All properties and OPS components are within expected ranges.
2. Unknown FCFP_2 feature: 581019816: [*]N\N=C\[*]

Feature Contribution

Top features for positive contribution

Fingerprint	Bit/Smiles	Feature Structure	Score	Moderate_Severe in training set
FCFP_10	547884906	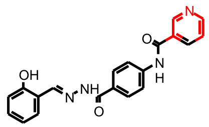 [*][c]1:[*]:[cH]:[cH]:n:[cH]:1	0.317	4 out of 4

FCFP_10	-1695756380	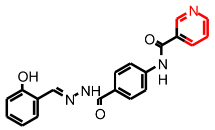 [*]1:[cH]:[cH]:[cH]:n :[cH]:1	0.285	10 out of 11
FCFP_10	-124655670	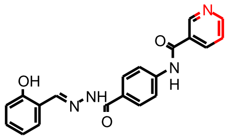 [*]:[cH]:[cH]:n:[*]	0.259	14 out of 16

Top Features for negative contribution

Fingerprint	Bit/Smiles	Feature Structure	Score	Moderate_Severe in training set
FCFP_10	-581879738	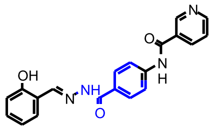 [*]NC(=O)[c]1:[cH]:[c H]:[*]:[cH]:[cH]:1	-1.29	0 out of 4
FCFP_10	-1925475824	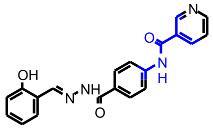 [*]:[cH]:[c](:[cH]:[*])C(=O)N[c](:[*]):[*]	-1.29	0 out of 4
FCFP_10	1175232969	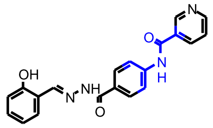 [*]:[cH]:[c](NC(=O)[c](:[*]):[*]):[cH]:[*]	-1.29	0 out of 4

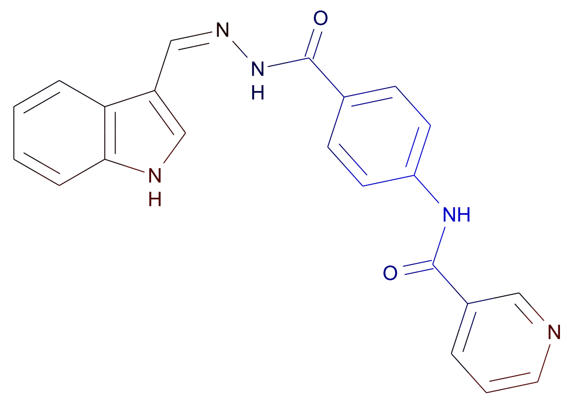

$C_{22}H_{17}N_5O_2$
Molecular Weight: 383.40268
ALogP: 2.611
Rotatable Bonds: 5
Acceptors: 4
Donors: 3

Model Prediction

Prediction: Mild
Probability: 0.694
Enrichment: 1.01
Bayesian Score: -3.64
Mahalanobis Distance: 6.77
Mahalanobis Distance p-value: 0.999

Prediction: Positive if the Bayesian score is above the estimated best cutoff value from minimizing the false positive and false negative rate.
Probability: The esimated probability that the sample is in the positive category. This assumes that the Bayesian score follows a normal distribution and is different from the prediction using a cutoff.
Enrichment: An estimate of enrichment, that is, the increased likelihood (versus random) of this sample being in the category.
Bayesian Score: The standard Laplacian-modified Bayesian score.
Mahalanobis Distance: The Mahalanobis distance (MD) is the distance to the center of the training data. The larger the MD, the less trustworthy the prediction.
Mahalanobis Distance p-value: The p-value gives the fraction of training data with an MD greater than or equal to the one for the given sample, assuming normally distributed data. The smaller the p-value, the less trustworthy the prediciton. For highly non-normal X properties (e.g., fingerprints), the MD p-value is wildly inaccurate.

Structural Similar Compounds

Name	ANTHRAQUINONE; 1-((2-HYDROXYETHYL)AMINO)-4-(METHYLAMINO)-	METHANE;TRIS(4-AMINOPHENYL)-	1-AMINO-4-BENZOYLAMINO-ANTHRAQUINONE
Structure	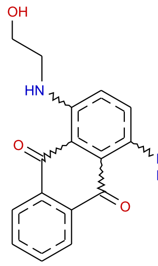	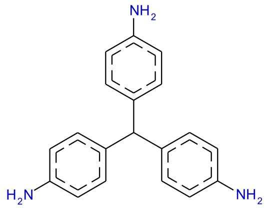	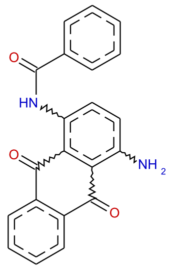
Actual Endpoint	Mild	Moderate_Severe	Mild
Predicted Endpoint	Mild	Moderate_Severe	Mild
Distance	0.647	0.660	0.695
Reference	28ZPAK 245;72	28ZPAK-;73;72	28ZPAK-;124;72

Model Applicability

Unknown features are fingerprint features in the query molecule, but not found or appearing too infrequently in the training set.

1. All properties and OPS components are within expected ranges.
2. Unknown FCFP_2 feature: 581019816: [*]N\N=C\[*]

Feature Contribution

Top features for positive contribution				
**Fingerprint**	**Bit/Smiles**	**Feature Structure**	**Score**	**Moderate_Severe in training set**
FCFP_10	547884906	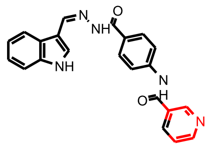 [*][c]1:[*]:[cH]:[cH]:n:[cH]:1	0.317	4 out of 4

FCFP_10	-1695756380	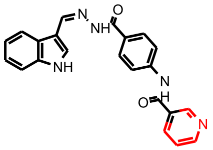<br>[*]1:[cH]:[cH]:[cH]:n<br>:[cH]:1	0.285	10 out of 11
FCFP_10	-124655670	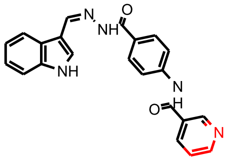<br>[*]:[cH]:[cH]:n:[*]	0.259	14 out of 16
**Top Features for negative contribution**				
**Fingerprint**	**Bit/Smiles**	**Feature Structure**	**Score**	**Moderate_Severe in training set**
FCFP_10	-1925475824	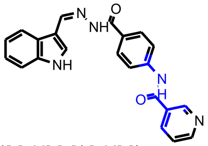<br>[*]:[cH]:[c](:[cH]:[*<br>])C(=O)N[c](:[*]):[*<br>]	-1.29	0 out of 4
FCFP_10	1175232969	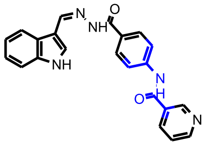<br>[*]:[cH]:[c](NC(=O)[c<br>](:[*]):[*]):[cH]:[*<br>]	-1.29	0 out of 4
FCFP_10	-581879738	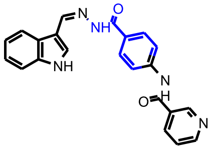<br>[*]NC(=O)[c]1:[cH]:[c<br>H]:[*]:[cH]:[cH]:1	-1.29	0 out of 4

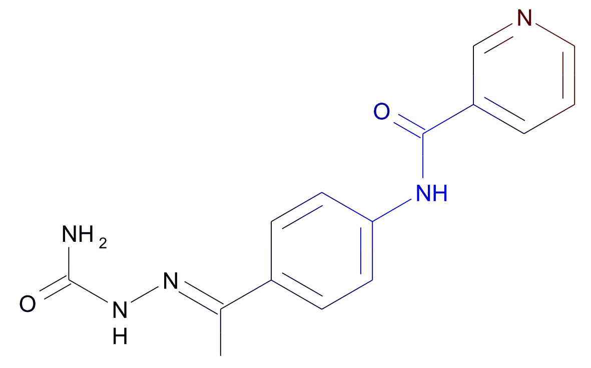

$C_{15}H_{15}N_5O_2$
Molecular Weight: 297.3119
ALogP: 0.423
Rotatable Bonds: 4
Acceptors: 4
Donors: 3

Structural Similar Compounds

Name	O-TOLUENESULFONAMIDE; 4-AMINO-N-(2-HYDROXYETHYL)-	2-NAPHTHALENESULFONIC ACID; 4-HYDROXY-7-(METHYLAMINO)-	p-Phenylenediamine; N;N-bis(2-hydroxyethyl)-; sulfate (1:1)
Structure	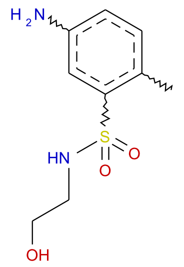	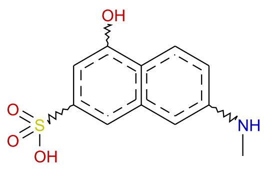	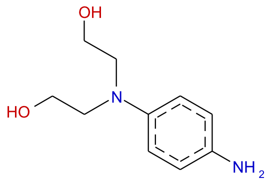
Actual Endpoint	Moderate_Severe	Mild	Mild
Predicted Endpoint	Moderate_Severe	Mild	Mild
Distance	0.601	0.633	0.640
Reference	28ZPAK-;200;72	28ZPAK 190;72	Prehled Prumyslove Toxikologie; Organicke Latky; Marhold; J. -;695;86

Model Prediction

Prediction: Mild
Probability: 0.709
Enrichment: 1.03
Bayesian Score: -3.36
Mahalanobis Distance: 6.71
Mahalanobis Distance p-value: 1

Prediction: Positive if the Bayesian score is above the estimated best cutoff value from minimizing the false positive and false negative rate.
Probability: The esimated probability that the sample is in the positive category. This assumes that the Bayesian score follows a normal distribution and is different from the prediction using a cutoff.
Enrichment: An estimate of enrichment, that is, the increased likelihood (versus random) of this sample being in the category.
Bayesian Score: The standard Laplacian-modified Bayesian score.
Mahalanobis Distance: The Mahalanobis distance (MD) is the distance to the center of the training data. The larger the MD, the less trustworthy the prediction.
Mahalanobis Distance p-value: The p-value gives the fraction of training data with an MD greater than or equal to the one for the given sample, assuming normally distributed data. The smaller the p-value, the less trustworthy the prediciton. For highly non-normal X properties (e.g., fingerprints), the MD p-value is wildly inaccurate.

Model Applicability

Unknown features are fingerprint features in the query molecule, but not found or appearing too infrequently in the training set.

1. All properties and OPS components are within expected ranges.
2. Unknown FCFP_2 feature: 581019816: [*]N\N=C\[*]

Feature Contribution

Top features for positive contribution				
Fingerprint	Bit/Smiles	Feature Structure	Score	Moderate_Severe in training set
FCFP_10	547884906	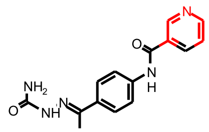 [*][c]1:[*]:[cH]:[cH]:n:[cH]:1	0.317	4 out of 4

FCFP_10	-1695756380	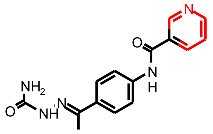 [*]1:[cH]:[cH]:[cH]:n :[cH]:1	0.285	10 out of 11
FCFP_10	-124655670	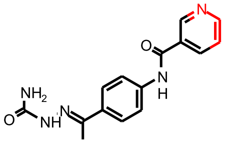 [*]:[cH]:[cH]:n:[*]	0.259	14 out of 16
**Top Features for negative contribution**				
**Fingerprint**	**Bit/Smiles**	**Feature Structure**	**Score**	**Moderate_Severe in training set**
FCFP_10	1175232969	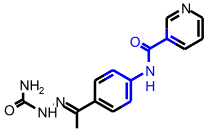 [*]:[cH]:[c](NC(=O)[c](:[*]):[*]):[cH]:[*]	-1.29	0 out of 4
FCFP_10	-1925475824	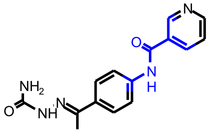 [*]:[cH]:[c](:[cH]:[*])C(=O)N[c](:[*]):[*]	-1.29	0 out of 4
FCFP_10	-790336137	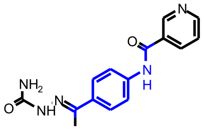 [*]C(=[*])N[c]1:[cH]: [cH]:[c](:[cH]:[cH]: 1)C(=[*])[*]	-0.507	0 out of 1

Sorafenib

TOPKAT_Ocular_Irritancy_Mild_vs_Moderate_Severe

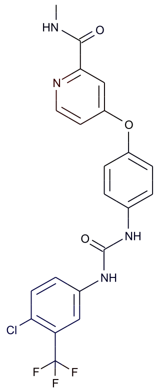

$C_{21}H_{16}ClF_3N_4O_3$
Molecular Weight: 464.82494
ALogP: 4.175
Rotatable Bonds: 6
Acceptors: 4
Donors: 3

Model Prediction

Prediction: Mild
Probability: 0.776
Enrichment: 1.13
Bayesian Score: -1.8
Mahalanobis Distance: 8.95
Mahalanobis Distance p-value: 0.537

Prediction: Positive if the Bayesian score is above the estimated best cutoff value from minimizing the false positive and false negative rate.
Probability: The esimated probability that the sample is in the positive category. This assumes that the Bayesian score follows a normal distribution and is different from the prediction using a cutoff.
Enrichment: An estimate of enrichment, that is, the increased likelihood (versus random) of this sample being in the category.
Bayesian Score: The standard Laplacian-modified Bayesian score.
Mahalanobis Distance: The Mahalanobis distance (MD) is the distance to the center of the training data. The larger the MD, the less trustworthy the prediction.
Mahalanobis Distance p-value: The p-value gives the fraction of training data with an MD greater than or equal to the one for the given sample, assuming normally distributed data. The smaller the p-value, the less trustworthy the prediciton. For highly non-normal X properties (e.g., fingerprints), the MD p-value is wildly inaccurate.

Structural Similar Compounds

Name	4;4'-DIAMINO-1;1'-DIANTHRIMIDE	5-NORBORNENE-2;3-DICARBOXYLIC ACID; 1;4;5;6;7;7-HEXACHLORO-	METHANE;TRIS(4-AMINOPHENYL)-
Structure	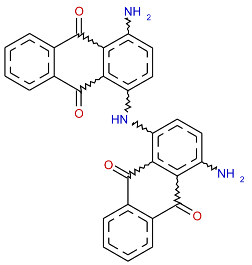	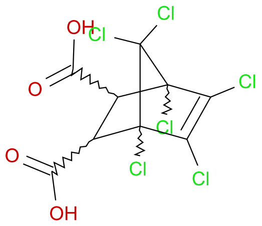	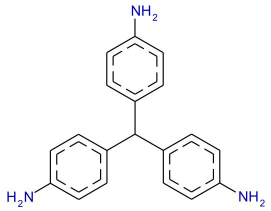
Actual Endpoint	Mild	Moderate_Severe	Moderate_Severe
Predicted Endpoint	Mild	Moderate_Severe	Moderate_Severe
Distance	0.799	0.816	0.827
Reference	28ZPAK-;125;72	28ZPAK-;92;72	28ZPAK-;73;72

Model Applicability

Unknown features are fingerprint features in the query molecule, but not found or appearing too infrequently in the training set.

1. All properties and OPS components are within expected ranges.

Feature Contribution

Top features for positive contribution

Fingerprint	Bit/Smiles	Feature Structure	Score	Moderate_Severe in training set
FCFP_10	-1695756380	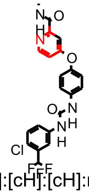 [*]1:[cH]:[cH]:[cH]:n:[cH]:1	0.285	10 out of 11

FCFP_10	-124655670	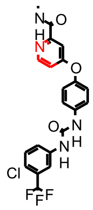<br>[*]:[cH]:[cH]:n:[*]	0.259	14 out of 16
FCFP_10	-885550502	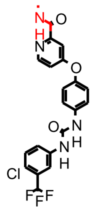<br>[*]C(=[*])NC	0.239	54 out of 64

Top Features for negative contribution

Fingerprint	Bit/Smiles	Feature Structure	Score	Moderate_Severe in training set
FCFP_10	2104062943	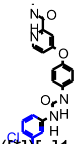<br>[*]C([*])([*])[c]1:[cH]:[*]:[cH]:[cH]:[c]:1Cl	-0.745	7 out of 24
FCFP_10	-174293376	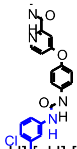<br>[*]N[c]1:[cH]:[cH]:[c](Cl):[c](:[cH]:1)C([*])([*])[*]	-0.507	0 out of 1
FCFP_10	-1549103449	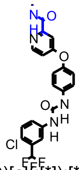<br>[*]NC(=O)[c](:[*]):[*]	-0.504	2 out of 6

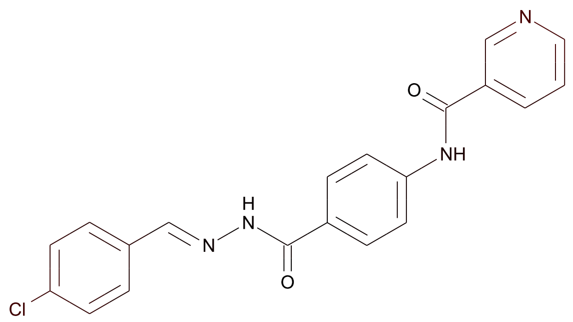

$C_{20}H_{15}ClN_4O_2$
Molecular Weight: 378.8117
ALogP: 2.981
Rotatable Bonds: 5
Acceptors: 4
Donors: 2

Model Prediction

Prediction: Irritant
Probability: 1
Enrichment: 1.18
Bayesian Score: 3.13
Mahalanobis Distance: 4.47
Mahalanobis Distance p-value: 1

Prediction: Positive if the Bayesian score is above the estimated best cutoff value from minimizing the false positive and false negative rate.
Probability: The esimated probability that the sample is in the positive category. This assumes that the Bayesian score follows a normal distribution and is different from the prediction using a cutoff.
Enrichment: An estimate of enrichment, that is, the increased likelihood (versus random) of this sample being in the category.
Bayesian Score: The standard Laplacian-modified Bayesian score.
Mahalanobis Distance: The Mahalanobis distance (MD) is the distance to the center of the training data. The larger the MD, the less trustworthy the prediction.
Mahalanobis Distance p-value: The p-value gives the fraction of training data with an MD greater than or equal to the one for the given sample, assuming normally distributed data. The smaller the p-value, the less trustworthy the prediciton. For highly non-normal X properties (e.g., fingerprints), the MD p-value is wildly inaccurate.

Structural Similar Compounds

Name	1-AMINO-4-BENZOYLAMINO-ANTHRAQUINONE	5-NORBORNENE-2;3-DICARBOXYLIC ACID; 1;4;5;6;7;7-HEXACHLORO-	ANTHRAQUINONE; 1-AMINO-4-HYDROXY-2-PHENOXY-
Structure	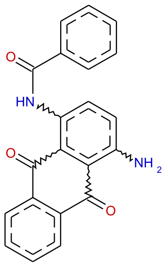	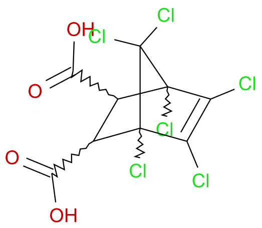	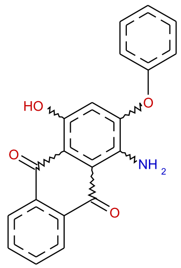
Actual Endpoint	Irritant	Irritant	Irritant
Predicted Endpoint	Irritant	Irritant	Irritant
Distance	0.563	0.579	0.640
Reference	28ZPAK-;124;72	28ZPAK-;92;72	28ZPAK 239;72

Model Applicability

Unknown features are fingerprint features in the query molecule, but not found or appearing too infrequently in the training set.

1. All properties and OPS components are within expected ranges.
2. Unknown FCFP_2 feature: 581019816: [*]N\N=C\[*]

Feature Contribution

Top features for positive contribution

Fingerprint	Bit/Smiles	Feature Structure	Score	Irritant in training set
FCFP_12	1747237384	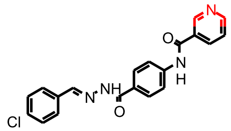 [*]:[cH]:n:[cH]:[*]	0.208	44 out of 44

FCFP_12	-1508180856	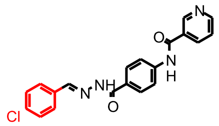 [*][c]1:[cH]:[cH]:[c](Cl):[cH]:[cH]:1	0.2	17 out of 17
FCFP_12	-124655670	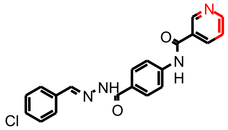 [*]:[cH]:[cH]:n:[*]	0.2	16 out of 16
**Top Features for negative contribution**				
**Fingerprint**	**Bit/Smiles**	**Feature Structure**	**Score**	**Irritant in training set**
FCFP_12	0	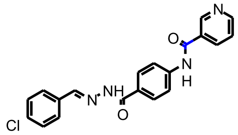 [*]C(=[*])[*]	0	1184 out of 1397
FCFP_12	1	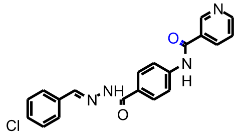 [*]=O	0	872 out of 1051
FCFP_12	-581879738	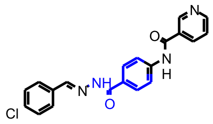 [*]NC(=O)[c]1:[cH]:[cH]:[*]:[cH]:[cH]:1	0	4 out of 5

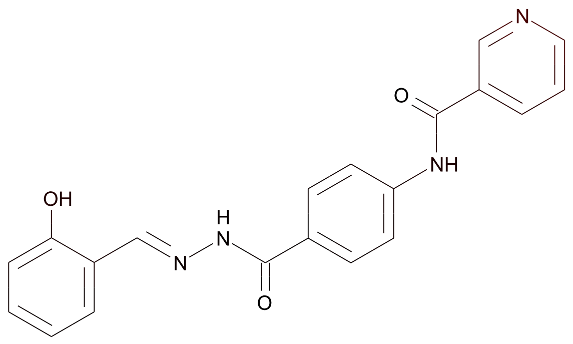

$C_{20}H_{16}N_4O_3$
Molecular Weight: 360.36604
ALogP: 2.075
Rotatable Bonds: 5
Acceptors: 5
Donors: 3

Structural Similar Compounds

Name	ANTHRAQUINONE; 1-((2-HYDROXYETHYL)AMINO)-4-(METHYLAMINO)-	Disperse Black 9	p-Acetophenetidide; 3'-(bis(2-hydroxyethyl)amino)-
Structure	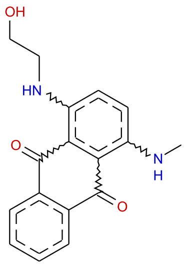	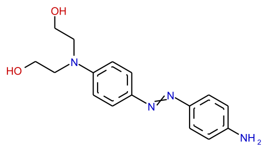	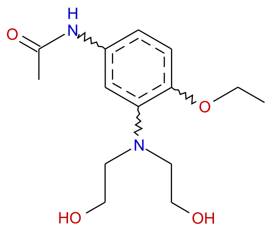
Actual Endpoint	Irritant	Non-Irritant	Irritant
Predicted Endpoint	Irritant	Non-Irritant	Irritant
Distance	0.561	0.622	0.679
Reference	28ZPAK 245;72	J. Am. Coll. Toxicol. 5(3):205;1986	Prehled Prumyslove Toxikologie; Organicke Latky; Marhold; J. -;645;86

Model Prediction

Prediction: Irritant
Probability: 1
Enrichment: 1.18
Bayesian Score: 2.75
Mahalanobis Distance: 4.94
Mahalanobis Distance p-value: 1

Prediction: Positive if the Bayesian score is above the estimated best cutoff value from minimizing the false positive and false negative rate.
Probability: The esimated probability that the sample is in the positive category. This assumes that the Bayesian score follows a normal distribution and is different from the prediction using a cutoff.
Enrichment: An estimate of enrichment, that is, the increased likelihood (versus random) of this sample being in the category.
Bayesian Score: The standard Laplacian-modified Bayesian score.
Mahalanobis Distance: The Mahalanobis distance (MD) is the distance to the center of the training data. The larger the MD, the less trustworthy the prediction.
Mahalanobis Distance p-value: The p-value gives the fraction of training data with an MD greater than or equal to the one for the given sample, assuming normally distributed data. The smaller the p-value, the less trustworthy the prediciton. For highly non-normal X properties (e.g., fingerprints), the MD p-value is wildly inaccurate.

Model Applicability

Unknown features are fingerprint features in the query molecule, but not found or appearing too infrequently in the training set.

1. All properties and OPS components are within expected ranges.
2. Unknown FCFP_2 feature: 581019816: [*]N\N=C\[*]

Feature Contribution

Top features for positive contribution				
Fingerprint	Bit/Smiles	Feature Structure	Score	Irritant in training set
FCFP_12	1747237384	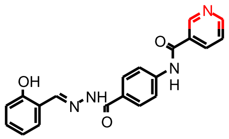 [*]:[cH]:n:[cH]:[*]	0.208	44 out of 44

FCFP_12	-124655670	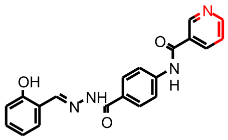 [*]:[cH]:[cH]:n:[*]	0.2	16 out of 16
FCFP_12	-1695756380	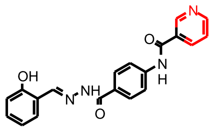 [*]1:[cH]:[cH]:[cH]:n :[cH]:1	0.194	11 out of 11

Top Features for negative contribution

Fingerprint	Bit/Smiles	Feature Structure	Score	Irritant in training set
FCFP_12	-1698724694	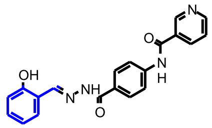 [*][c]1:[cH]:[cH]:[cH]:[cH]:[c]:1C=[*]	-0.0964	107 out of 146
FCFP_12	-453677277	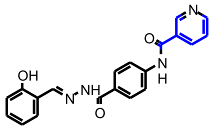 [*]C(=[*])[c]1:[cH]:[*]:[cH]:[cH]:[cH]:1	0	264 out of 323
FCFP_12	203677720	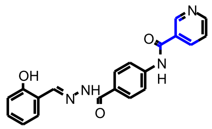 [*]C(=[*])[c](:[cH]:[*]):[cH]:[*]	0	319 out of 382

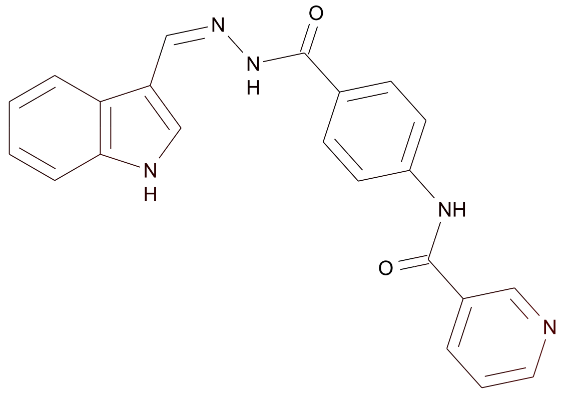

$C_{22}H_{17}N_5O_2$
Molecular Weight: 383.40268
ALogP: 2.611
Rotatable Bonds: 5
Acceptors: 4
Donors: 3

Model Prediction

Prediction: Irritant
Probability: 1
Enrichment: 1.18
Bayesian Score: 3.04
Mahalanobis Distance: 5.63
Mahalanobis Distance p-value: 1

Prediction: Positive if the Bayesian score is above the estimated best cutoff value from minimizing the false positive and false negative rate.
Probability: The esimated probability that the sample is in the positive category. This assumes that the Bayesian score follows a normal distribution and is different from the prediction using a cutoff.
Enrichment: An estimate of enrichment, that is, the increased likelihood (versus random) of this sample being in the category.
Bayesian Score: The standard Laplacian-modified Bayesian score.
Mahalanobis Distance: The Mahalanobis distance (MD) is the distance to the center of the training data. The larger the MD, the less trustworthy the prediction.
Mahalanobis Distance p-value: The p-value gives the fraction of training data with an MD greater than or equal to the one for the given sample, assuming normally distributed data. The smaller the p-value, the less trustworthy the prediciton. For highly non-normal X properties (e.g., fingerprints), the MD p-value is wildly inaccurate.

Structural Similar Compounds

Name	ANTHRAQUINONE; 1-((2-HYDROXYETHYL)AMINO)-4-(METHYLAMINO)-	METHANE;TRIS(4-AMINOPHENYL)-	1-AMINO-4-BENZOYLAMINO-ANTHRAQUINONE
Structure	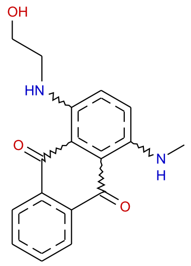	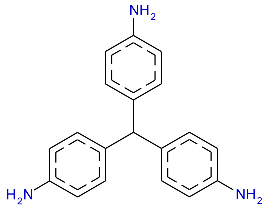	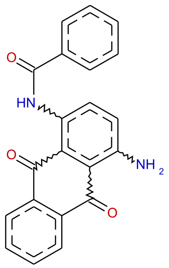
Actual Endpoint	Irritant	Irritant	Irritant
Predicted Endpoint	Irritant	Irritant	Irritant
Distance	0.639	0.652	0.697
Reference	28ZPAK 245;72	28ZPAK-;73;72	28ZPAK-;124;72

Model Applicability

Unknown features are fingerprint features in the query molecule, but not found or appearing too infrequently in the training set.

1. All properties and OPS components are within expected ranges.
2. Unknown FCFP_2 feature: 581019816: [*]N\N=C\[*]

Feature Contribution

Top features for positive contribution

Fingerprint	Bit/Smiles	Feature Structure	Score	Irritant in training set
FCFP_12	1747237384	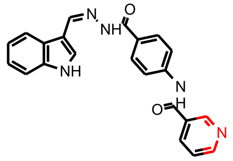 [*]:[cH]:n:[cH]:[*]	0.208	44 out of 44

FCFP_12	-124655670	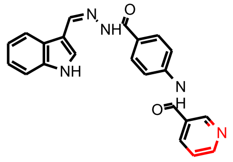 [*]:[cH]:[cH]:n:[*]	0.2	16 out of 16
FCFP_12	-1695756380	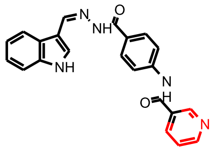 [*]1:[cH]:[cH]:[cH]:n :[cH]:1	0.194	11 out of 11
**Top Features for negative contribution**				
**Fingerprint**	**Bit/Smiles**	**Feature Structure**	**Score**	**Irritant in training set**
FCFP_12	-1925475824	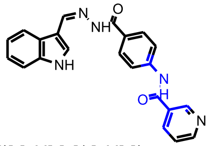 [*]:[cH]:[c](:[cH]:[*])C(=O)N[c](:[*]):[*]	0	4 out of 5
FCFP_12	1175232969	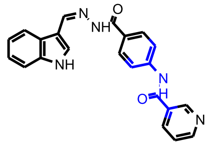 [*]:[cH]:[c](NC(=O)[c](:[*]):[*]):[cH]:[*]	0	4 out of 5
FCFP_12	-773983804	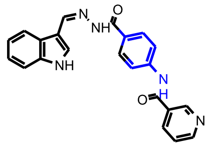 [*]N[c]1:[cH]:[*]:[c] ([*]):[cH]:[cH]:1	0	102 out of 121

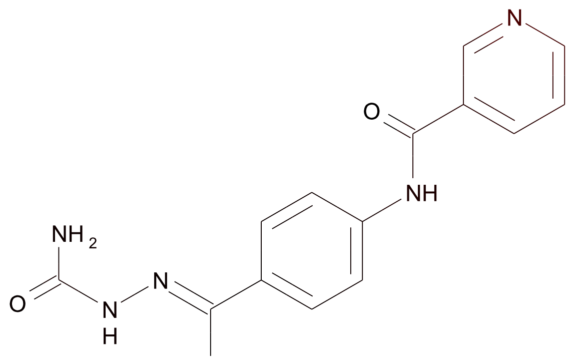

$C_{15}H_{15}N_5O_2$
Molecular Weight: 297.3119
ALogP: 0.423
Rotatable Bonds: 4
Acceptors: 4
Donors: 3

Structural Similar Compounds

Name	O-TOLUENESULFONAMIDE; 4-AMINO-N-(2-HYDROXYETHYL)-	2-NAPHTHALENESULFONIC ACID; 4-HYDROXY-7-(METHYLAMINO)-	p-Phenylenediamine; N;N-bis(2-hydroxyethyl)-; sulfate (1:1)
Structure	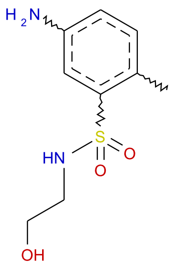	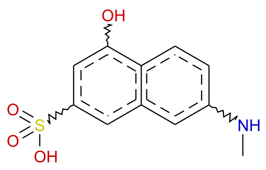	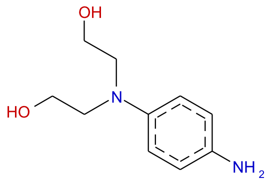
Actual Endpoint	Irritant	Irritant	Irritant
Predicted Endpoint	Irritant	Irritant	Irritant
Distance	0.599	0.622	0.633
Reference	28ZPAK-;200;72	28ZPAK 190;72	Prehled Prumyslove Toxikologie; Organicke Latky; Marhold; J. -;695;86

Model Prediction

Prediction: Irritant
Probability: 1
Enrichment: 1.18
Bayesian Score: 2.29
Mahalanobis Distance: 6.2
Mahalanobis Distance p-value: 1

Prediction: Positive if the Bayesian score is above the estimated best cutoff value from minimizing the false positive and false negative rate.
Probability: The esimated probability that the sample is in the positive category. This assumes that the Bayesian score follows a normal distribution and is different from the prediction using a cutoff.
Enrichment: An estimate of enrichment, that is, the increased likelihood (versus random) of this sample being in the category.
Bayesian Score: The standard Laplacian-modified Bayesian score.
Mahalanobis Distance: The Mahalanobis distance (MD) is the distance to the center of the training data. The larger the MD, the less trustworthy the prediction.
Mahalanobis Distance p-value: The p-value gives the fraction of training data with an MD greater than or equal to the one for the given sample, assuming normally distributed data. The smaller the p-value, the less trustworthy the prediciton. For highly non-normal X properties (e.g., fingerprints), the MD p-value is wildly inaccurate.

Model Applicability

Unknown features are fingerprint features in the query molecule, but not found or appearing too infrequently in the training set.

1. All properties and OPS components are within expected ranges.
2. Unknown FCFP_2 feature: 581019816: [*]N\N=C\[*]

Feature Contribution

Top features for positive contribution

Fingerprint	Bit/Smiles	Feature Structure	Score	Irritant in training set
FCFP_12	1747237384	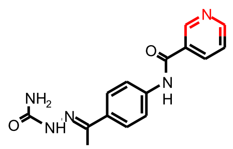 [*]:[cH]:n:[cH]:[*]	0.208	44 out of 44

FCFP_12	-124655670	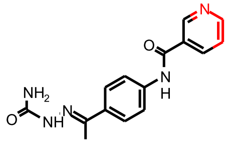 [*]:[cH]:[cH]:n:[*]	0.2	16 out of 16
FCFP_12	-1695756380	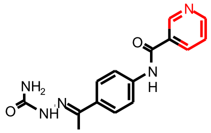 [*]1:[cH]:[cH]:[cH]:n :[cH]:1	0.194	11 out of 11

Top Features for negative contribution

Fingerprint	Bit/Smiles	Feature Structure	Score	Irritant in training set
FCFP_12	0	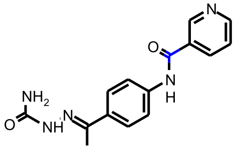 [*]C(=[*])[*]	0	1184 out of 1397
FCFP_12	1	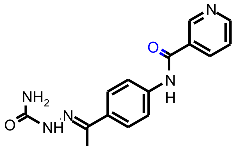 [*]=O	0	872 out of 1051
FCFP_12	203677720	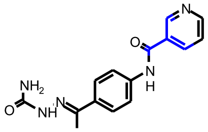 [*]C(=[*])[c](:[cH]:[*]):[cH]:[*]	0	319 out of 382

Sorafenib

TOPKAT_Ocular_Irritancy_None_vs_Irritant

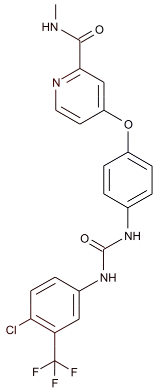

$C_{21}H_{16}ClF_3N_4O_3$
Molecular Weight: 464.82494
ALogP: 4.175
Rotatable Bonds: 6
Acceptors: 4
Donors: 3

Structural Similar Compounds

Name	BENZANILIDE;2';2'''-DITHIOBIS-	4;4'-DIAMINO-1;1'-DIANTHRIMIDE	5-NORBORNENE-2;3-DICARBOXYLIC ACID; 1;4;5;6;7;7-HEXACHLORO-
Structure	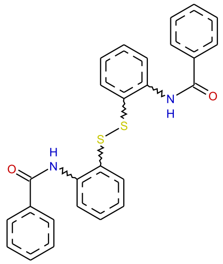	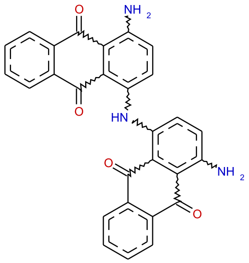	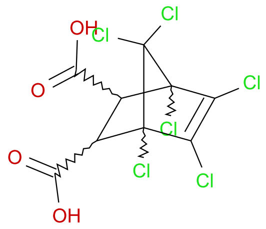
Actual Endpoint	Non-Irritant	Irritant	Irritant
Predicted Endpoint	Non-Irritant	Irritant	Irritant
Distance	0.743	0.791	0.801
Reference	28ZPAK-;173;72	28ZPAK-;125;72	28ZPAK-;92;72

Model Prediction

Prediction: Irritant
Probability: 1
Enrichment: 1.18
Bayesian Score: 3.04
Mahalanobis Distance: 6.28
Mahalanobis Distance p-value: 1

Prediction: Positive if the Bayesian score is above the estimated best cutoff value from minimizing the false positive and false negative rate.
Probability: The esimated probability that the sample is in the positive category. This assumes that the Bayesian score follows a normal distribution and is different from the prediction using a cutoff.
Enrichment: An estimate of enrichment, that is, the increased likelihood (versus random) of this sample being in the category.
Bayesian Score: The standard Laplacian-modified Bayesian score.
Mahalanobis Distance: The Mahalanobis distance (MD) is the distance to the center of the training data. The larger the MD, the less trustworthy the prediction.
Mahalanobis Distance p-value: The p-value gives the fraction of training data with an MD greater than or equal to the one for the given sample, assuming normally distributed data. The smaller the p-value, the less trustworthy the prediciton. For highly non-normal X properties (e.g., fingerprints), the MD p-value is wildly inaccurate.

Model Applicability

Unknown features are fingerprint features in the query molecule, but not found or appearing too infrequently in the training set.

1. All properties and OPS components are within expected ranges.

Feature Contribution

Top features for positive contribution				
**Fingerprint**	**Bit/Smiles**	**Feature Structure**	**Score**	**Irritant in training set**
FCFP_12	1747237384	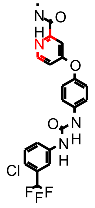 [*]:[cH]:n:[cH]:[*]	0.208	44 out of 44

FCFP_12	-124655670	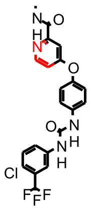 [*]:[cH]:[cH]:n:[*]	0.2	16 out of 16
FCFP_12	-1539132615	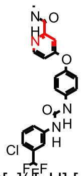 [*]C(=[*])[c](:[cH]:[*]):n:[*]	0.197	13 out of 13
**Top Features for negative contribution**				
**Fingerprint**	**Bit/Smiles**	**Feature Structure**	**Score**	**Irritant in training set**
FCFP_12	-747629521	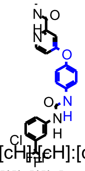 [*]N[c]1:[cH]:[cH]:[c](O[c](:[*]):[*]):[cH]:[cH]:1	-0.268	1 out of 2
FCFP_12	702861189	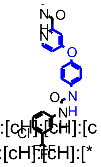 [*]N[c]1:[cH]:[cH]:[c](O[c]2:[cH]:[cH]:[*]:[c]([*]):[cH]:2):[cH]:[cH]:1	-0.268	1 out of 2
FCFP_12	-215363676	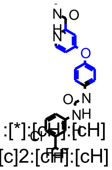 [*][c]1:[*]:[cH]:[cH]:[c](O[c]2:[cH]:[cH]:[*]:[cH]:[cH]:2):[cH]:1	0	4 out of 5

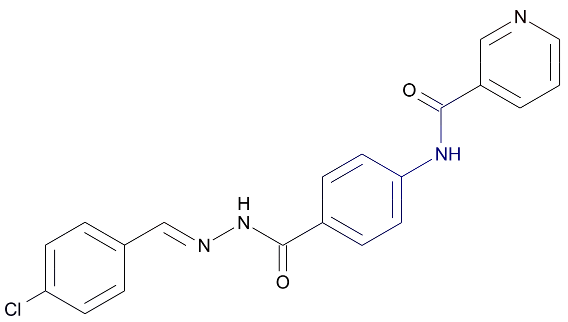

$C_{20}H_{15}ClN_4O_2$

Molecular Weight: 378.8117

ALogP: 2.981

Rotatable Bonds: 5

Acceptors: 4

Donors: 2

Model Prediction

Prediction: Non-Irritant

Probability: 0.884

Enrichment: 0.959

Bayesian Score: -2.93

Mahalanobis Distance: 6.35

Mahalanobis Distance p-value: 1

Prediction: Positive if the Bayesian score is above the estimated best cutoff value from minimizing the false positive and false negative rate.
Probability: The esimated probability that the sample is in the positive category. This assumes that the Bayesian score follows a normal distribution and is different from the prediction using a cutoff.
Enrichment: An estimate of enrichment, that is, the increased likelihood (versus random) of this sample being in the category.
Bayesian Score: The standard Laplacian-modified Bayesian score.
Mahalanobis Distance: The Mahalanobis distance (MD) is the distance to the center of the training data. The larger the MD, the less trustworthy the prediction.
Mahalanobis Distance p-value: The p-value gives the fraction of training data with an MD greater than or equal to the one for the given sample, assuming normally distributed data. The smaller the p-value, the less trustworthy the prediciton. For highly non-normal X properties (e.g., fingerprints), the MD p-value is wildly inaccurate.

Structural Similar Compounds

Name	5-Norbornene-2,3-dicarboxylic acid, 1,4,5,6,7,7-hexachloro-	1-Amino-2-bromo-4-hydroxyanthraquinone	1-Piperazineacetic acid, 4-(2-hydroxyethyl)-alpha-phenyl-, 2,6-xyly l ester, monohydrochloride
Structure	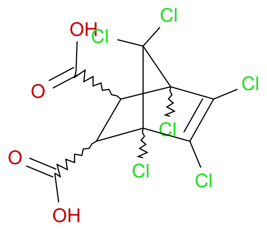	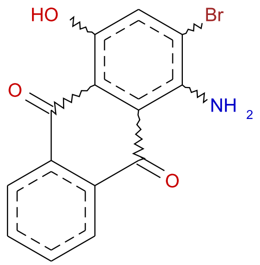	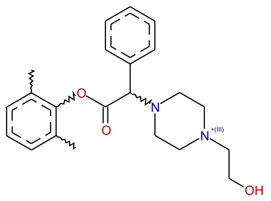
Actual Endpoint	Irritant	Non-Irritant	Irritant
Predicted Endpoint	Irritant	Non-Irritant	Irritant
Distance	0.635	0.738	0.773
Reference	85JCAE "Prehled Prumyslove Toxikologie; Organicke Latky," Marhold, J., Prague , Czechoslovakia, Avicenum, 1986 Volume(issue)/page/year: -,581,1986	28ZPAK -,83,72	BCFAAI Bollettino Chimico Farmaceutico. (Societa Editoriale Farmaceutica, Vi a Ausonio 12, 20123 Milan, Italy) V.33- 1894- Volume(issue)/page/year: 107,3 10,1968

Model Applicability

Unknown features are fingerprint features in the query molecule, but not found or appearing too infrequently in the training set.

1. All properties and OPS components are within expected ranges.

Feature Contribution

Top features for positive contribution				
Fingerprint	Bit/Smiles	Feature Structure	Score	Irritant in training set

FCFP_12	-124655670	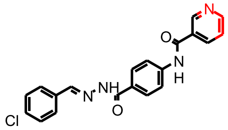<br>[*]:[cH]:[cH]:n:[*]	0.0821	13 out of 13
FCFP_12	-2100785893	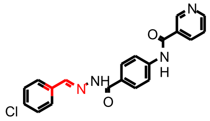<br>[*]\N=C\[c](:[*]):[*]	0.081	11 out of 11
FCFP_12	-1695756380	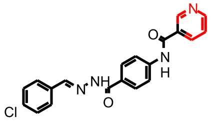<br>[*]1:[cH]:[cH]:[cH]:n<br>:[cH]:1	0.0772	7 out of 7
**Top Features for negative contribution**				
**Fingerprint**	**Bit/Smiles**	**Feature Structure**	**Score**	**Irritant in training set**
FCFP_12	-1838187238	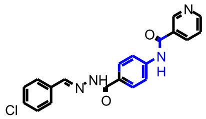<br>[*]C(=[*])N[c]1:[cH]:<br>[cH]:[*]:[cH]:[cH]:1	-0.692	5 out of 12
FCFP_12	1294255210	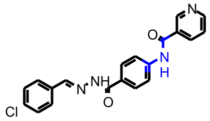<br>[*]C(=[*])N[c](:[*]):<br>[*]	-0.486	12 out of 22

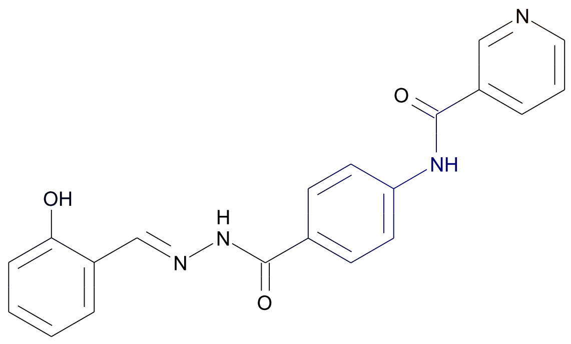

$C_{20}H_{16}N_4O_3$
Molecular Weight: 360.36604
ALogP: 2.075
Rotatable Bonds: 5
Acceptors: 5
Donors: 3

Model Prediction

Prediction: Non-Irritant
Probability: 0.883
Enrichment: 0.959
Bayesian Score: -2.93
Mahalanobis Distance: 6.89
Mahalanobis Distance p-value: 0.998

Prediction: Positive if the Bayesian score is above the estimated best cutoff value from minimizing the false positive and false negative rate.
Probability: The esimated probability that the sample is in the positive category. This assumes that the Bayesian score follows a normal distribution and is different from the prediction using a cutoff.
Enrichment: An estimate of enrichment, that is, the increased likelihood (versus random) of this sample being in the category.
Bayesian Score: The standard Laplacian-modified Bayesian score.
Mahalanobis Distance: The Mahalanobis distance (MD) is the distance to the center of the training data. The larger the MD, the less trustworthy the prediction.
Mahalanobis Distance p-value: The p-value gives the fraction of training data with an MD greater than or equal to the one for the given sample, assuming normally distributed data. The smaller the p-value, the less trustworthy the prediciton. For highly non-normal X properties (e.g., fingerprints), the MD p-value is wildly inaccurate.

Structural Similar Compounds

Name	8-Methylamino-4-hydroxy-2-naphthalene sulfonic acid	p-Acetophenetidide, 3'-(bis(2-hydroxyethyl)amino)-	C.I. Fluorescent Brightening Agent 24
Structure	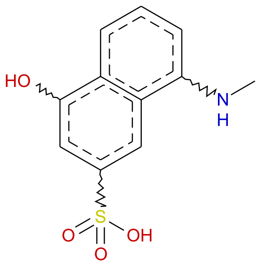	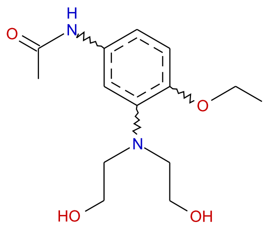	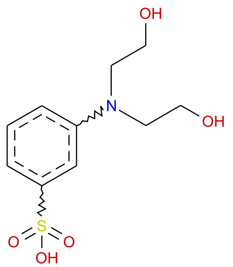
Actual Endpoint	Non-Irritant	Non-Irritant	Irritant
Predicted Endpoint	Non-Irritant	Non-Irritant	Non-Irritant
Distance	0.746	0.750	0.786
Reference	28ZPAK -,190,72	28ZPAK -,100,72	MVCRB3 MVC-Report. (Stockholm, Sweden) No.1-2, 1972-73. Discontinued. Volu me(issue)/page/year: 2,193,1973

Model Applicability

Unknown features are fingerprint features in the query molecule, but not found or appearing too infrequently in the training set.

1. All properties and OPS components are within expected ranges.

Feature Contribution

Top features for positive contribution

Fingerprint	Bit/Smiles	Feature Structure	Score	Irritant in training set
FCFP_12	-309153329	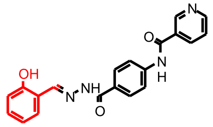 [*]=C[c]1:[cH]:[cH]:[cH]:[cH]:[c]:1O	0.0821	13 out of 13

FCFP_12	-124655670	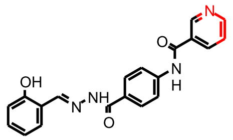 [*]:[cH]:[cH]:n:[*]	0.0821	13 out of 13
FCFP_12	-2100785893	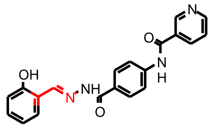 [*]\N=C\[c](:[*]):[*]	0.081	11 out of 11
**Top Features for negative contribution**				
**Fingerprint**	**Bit/Smiles**	**Feature Structure**	**Score**	**Irritant in training set**
FCFP_12	-1838187238	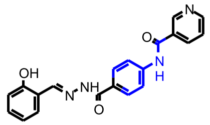 [*]C(=[*])N[c]1:[cH]:[cH]:[*]:[cH]:[cH]:1	-0.692	5 out of 12
FCFP_12	1294255210	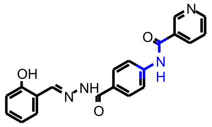 [*]C(=[*])N[c](:[*]):[*]	-0.486	12 out of 22
FCFP_12	-773983804	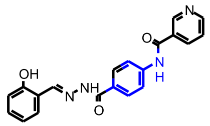 [*]N[c]1:[cH]:[*]:[c]([*]):[cH]:[cH]:1	-0.444	46 out of 79

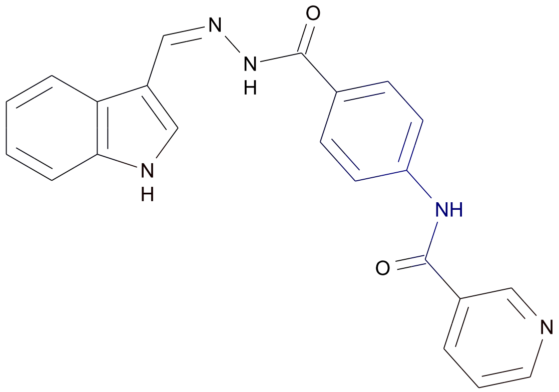

$C_{22}H_{17}N_5O_2$
Molecular Weight: 383.40268
ALogP: 2.611
Rotatable Bonds: 5
Acceptors: 4
Donors: 3

Model Prediction

Prediction: Non-Irritant
Probability: 0.923
Enrichment: 1
Bayesian Score: -2.45
Mahalanobis Distance: 6.5
Mahalanobis Distance p-value: 1

Prediction: Positive if the Bayesian score is above the estimated best cutoff value from minimizing the false positive and false negative rate.
Probability: The esimated probability that the sample is in the positive category. This assumes that the Bayesian score follows a normal distribution and is different from the prediction using a cutoff.
Enrichment: An estimate of enrichment, that is, the increased likelihood (versus random) of this sample being in the category.
Bayesian Score: The standard Laplacian-modified Bayesian score.
Mahalanobis Distance: The Mahalanobis distance (MD) is the distance to the center of the training data. The larger the MD, the less trustworthy the prediction.
Mahalanobis Distance p-value: The p-value gives the fraction of training data with an MD greater than or equal to the one for the given sample, assuming normally distributed data. The smaller the p-value, the less trustworthy the prediciton. For highly non-normal X properties (e.g., fingerprints), the MD p-value is wildly inaccurate.

Structural Similar Compounds

Name	5-Norbornene-2,3-dicarboxylic acid, 1,4,5,6,7,7-hexachloro-	p-Acetophenetidide, 3'-(bis(2-hydroxyethyl)amino)-	1-Amino-2-bromo-4-hydroxyanthraquinone
Structure	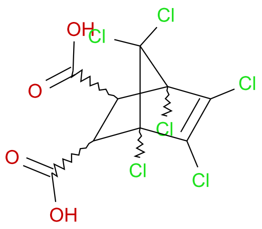	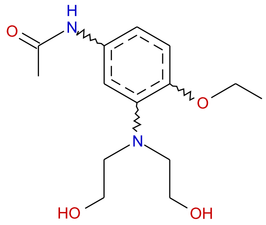	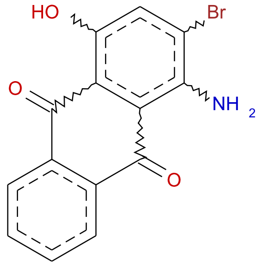
Actual Endpoint	Irritant	Non-Irritant	Non-Irritant
Predicted Endpoint	Irritant	Non-Irritant	Non-Irritant
Distance	0.764	0.815	0.817
Reference	85JCAE "Prehled Prumyslove Toxikologie; Organicke Latky," Marhold, J., Prague , Czechoslovakia, Avicenum, 1986 Volume(issue)/page/year: -,581,1986	28ZPAK -,100,72	28ZPAK -,83,72

Model Applicability

Unknown features are fingerprint features in the query molecule, but not found or appearing too infrequently in the training set.

1. All properties and OPS components are within expected ranges.
2. Unknown FCFP_2 feature: 1618184456: [*][c]1:[*]:[*]:[nH]:c:1

Feature Contribution

Top features for positive contribution				
Fingerprint	Bit/Smiles	Feature Structure	Score	Irritant in training set

FCFP_12	-124655670	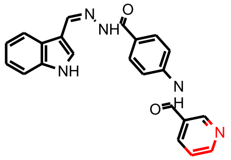 [*]:[cH]:[cH]:n:[*]	0.0821	13 out of 13
FCFP_12	-2100785893	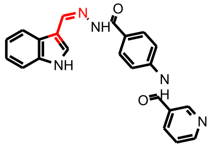 [*]\N=C\[c](:[*]):[*]	0.081	11 out of 11
FCFP_12	-1695756380	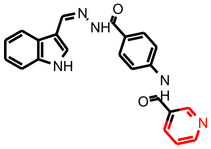 [*]1:[cH]:[cH]:[cH]:n:[cH]:1	0.0772	7 out of 7
**Top Features for negative contribution**				
**Fingerprint**	**Bit/Smiles**	**Feature Structure**	**Score**	**Irritant in training set**
FCFP_12	-1838187238	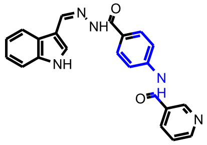 [*]C(=[*])N[c]1:[cH]:[cH]:[*]:[cH]:[cH]:1	-0.692	5 out of 12
FCFP_12	1294255210	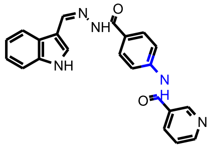 [*]C(=[*])N[c](:[*]):[*]	-0.486	12 out of 22

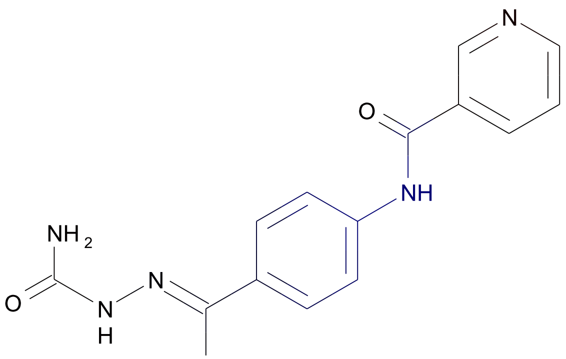

$C_{15}H_{15}N_5O_2$
Molecular Weight: 297.3119
ALogP: 0.423
Rotatable Bonds: 4
Acceptors: 4
Donors: 3

Model Prediction

Prediction: Non-Irritant
Probability: 0.889
Enrichment: 0.965
Bayesian Score: -2.87
Mahalanobis Distance: 6.43
Mahalanobis Distance p-value: 1

Prediction: Positive if the Bayesian score is above the estimated best cutoff value from minimizing the false positive and false negative rate.
Probability: The esimated probability that the sample is in the positive category. This assumes that the Bayesian score follows a normal distribution and is different from the prediction using a cutoff.
Enrichment: An estimate of enrichment, that is, the increased likelihood (versus random) of this sample being in the category.
Bayesian Score: The standard Laplacian-modified Bayesian score.
Mahalanobis Distance: The Mahalanobis distance (MD) is the distance to the center of the training data. The larger the MD, the less trustworthy the prediction.
Mahalanobis Distance p-value: The p-value gives the fraction of training data with an MD greater than or equal to the one for the given sample, assuming normally distributed data. The smaller the p-value, the less trustworthy the prediciton. For highly non-normal X properties (e.g., fingerprints), the MD p-value is wildly inaccurate.

Structural Similar Compounds

Name	o-Toluenesulfonamide, 4-amino-N-(2-hydroxyethyl)-	8-Methylamino-4-hydroxy-2-naphthalene sulfonic acid	Acetanilide, 4'-(2-hydroxyethylsulfonyl)-
Structure	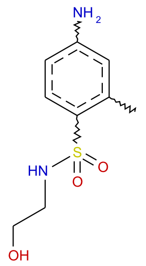	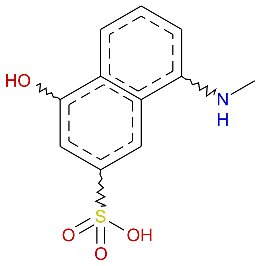	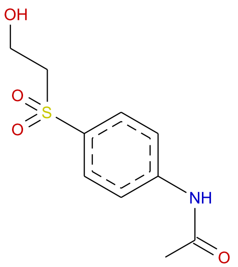
Actual Endpoint	Irritant	Non-Irritant	Non-Irritant
Predicted Endpoint	Non-Irritant	Non-Irritant	Non-Irritant
Distance	0.624	0.667	0.674
Reference	85JCAE "Prehled Prumyslove Toxikologie; Organicke Latky," Marhold, J., Prague , Czechoslovakia, Avicenum, 1986 Volume(issue)/page/year: -,1076,1986	28ZPAK -,190,72	28ZPAK -,533,72

Model Applicability

Unknown features are fingerprint features in the query molecule, but not found or appearing too infrequently in the training set.

1. All properties and OPS components are within expected ranges.

Feature Contribution

Top features for positive contribution				
Fingerprint	Bit/Smiles	Feature Structure	Score	Irritant in training set

FCFP_12	-124655670	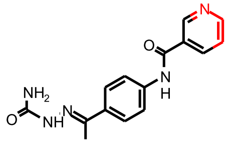<br>[*]:[cH]:[cH]:n:[*]	0.0821	13 out of 13
FCFP_12	-1695756380	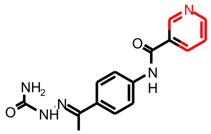<br>[*]1:[cH]:[cH]:[cH]:n<br>:[cH]:1	0.0772	7 out of 7
FCFP_12	730557100	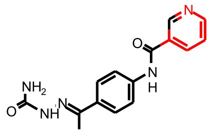<br>[*][c]1:[*]:n:[cH]:[c<br>H]:[cH]:1	0.0756	6 out of 6

Top Features for negative contribution

**Fingerprint**	**Bit/Smiles**	**Feature Structure**	**Score**	**Irritant in training set**
FCFP_12	-1838187238	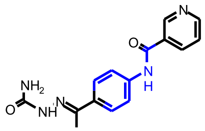<br>[*]C(=[*])N[c]1:[cH]:<br>[cH]:[*]:[cH]:[cH]:1	-0.692	5 out of 12
FCFP_12	1294255210	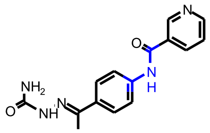<br>[*]C(=[*])N[c](:[*]):<br>[*]	-0.486	12 out of 22

Sorafenib

TOPKAT_Skin_Irritancy_None_vs_Irritant

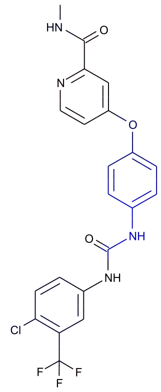

$C_{21}H_{16}ClF_3N_4O_3$

Molecular Weight: 464.82494

ALogP: 4.175

Rotatable Bonds: 6

Acceptors: 4

Donors: 3

Model Prediction

Prediction: Non-Irritant

Probability: 0.264

Enrichment: 0.287

Bayesian Score: -5.23

Mahalanobis Distance: 8.27

Mahalanobis Distance p-value: 0.791

Prediction: Positive if the Bayesian score is above the estimated best cutoff value from minimizing the false positive and false negative rate.
Probability: The esimated probability that the sample is in the positive category. This assumes that the Bayesian score follows a normal distribution and is different from the prediction using a cutoff.
Enrichment: An estimate of enrichment, that is, the increased likelihood (versus random) of this sample being in the category.
Bayesian Score: The standard Laplacian-modified Bayesian score.
Mahalanobis Distance: The Mahalanobis distance (MD) is the distance to the center of the training data. The larger the MD, the less trustworthy the prediction.
Mahalanobis Distance p-value: The p-value gives the fraction of training data with an MD greater than or equal to the one for the given sample, assuming normally distributed data. The smaller the p-value, the less trustworthy the prediciton. For highly non-normal X properties (e.g., fingerprints), the MD p-value is wildly inaccurate.

Structural Similar Compounds

Name	5-Norbornene-2,3-dicarboxylic acid, 1,4,5,6,7,7-hexachloro-	Benzenesulfonic acid, 2,2'-(4,4'-biphenylylenedivinylene)di-, disod ium salt	Sulfide, bis(4-t-butyl-m-cresyl)-
Structure	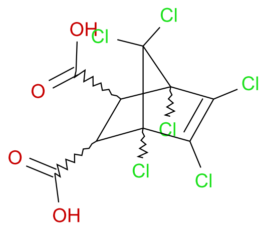	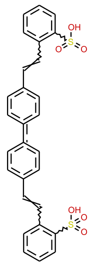	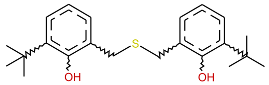
Actual Endpoint	Irritant	Irritant	Irritant
Predicted Endpoint	Irritant	Non-Irritant	Irritant
Distance	0.844	0.871	0.884
Reference	85JCAE "Prehled Prumyslove Toxikologie; Organicke Latky," Marhold, J., Prague , Czechoslovakia, Avicenum, 1986 Volume(issue)/page/year: -,581,1986	MVCRB3 MVC-Report. (Stockholm, Sweden) No.1-2, 1972-73. Discontinued. Volu me(issue)/page/year: 2,193,1973	AMIHBC AMA Archives of Industrial Hygiene and Occupational Medicine. (Chicago , IL) V.2-10, 1950-54. For publisher information, see AEHLAU. Volume(issue)/pag e/year: 5,311,1952

Model Applicability

Unknown features are fingerprint features in the query molecule, but not found or appearing too infrequently in the training set.

1. All properties and OPS components are within expected ranges.

Feature Contribution

Top features for positive contribution				
Fingerprint	Bit/Smiles	Feature Structure	Score	Irritant in training set

FCFP_12	-124655670	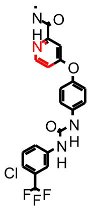<br>[*]:[cH]:[cH]:n:[*]	0.0821	13 out of 13
FCFP_12	-1539132615	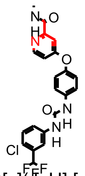<br>[*]C(=[*])[c](:[cH]:[<br>*]):n:[*]	0.0795	9 out of 9
FCFP_12	-1695756380	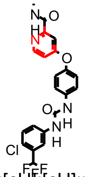<br>[*]1:[cH]:[cH]:[cH]:n<br>:[cH]:1	0.0772	7 out of 7
**Top Features for negative contribution**				
**Fingerprint**	**Bit/Smiles**	**Feature Structure**	**Score**	**Irritant in training set**
FCFP_12	-789307649	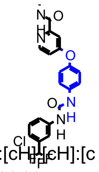<br>[*]O[c]1:[cH]:[cH]:[c<br>](NC(=[*])[*]):[cH]:<br>[cH]:1	-1.54	0 out of 4
FCFP_12	-1838187238	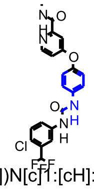<br>[*]C(=[*])N[c]1:[cH]:<br>[cH]:[*]:[cH]:[cH]:1	-0.692	5 out of 12

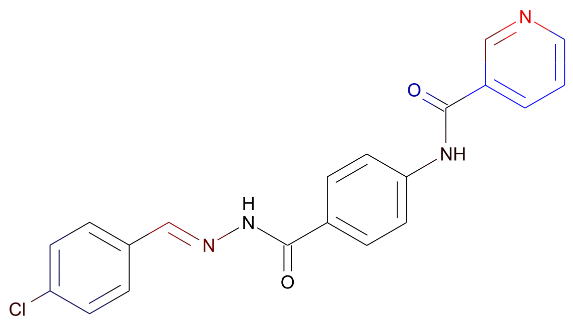

$C_{20}H_{15}ClN_4O_2$
Molecular Weight: 378.8117
ALogP: 2.981
Rotatable Bonds: 5
Acceptors: 4
Donors: 2

Model Prediction

Prediction: 11.1
Unit: mg/kg_body_weight/day
Mahalanobis Distance: 12.5
Mahalanobis Distance p-value: 1.69e-006

Mahalanobis Distance: The Mahalanobis distance (MD) is a generalization of the Euclidean distance that accounts for correlations among the X properties. It is calculated as the distance to the center of the training data. The larger the MD, the less trustworthy the prediction.
Mahalanobis Distance p-value: The p-value gives the fraction of training data with an MD greater than or equal to the one for the given sample, assuming normally distributed data. The smaller the p-value, the less trustworthy the prediciton. For highly non-normal X properties (e.g., fingerprints), the MD p-value is wildly inaccurate.

Structural Similar Compounds

Name	422	455	Phenolphthalein
Structure	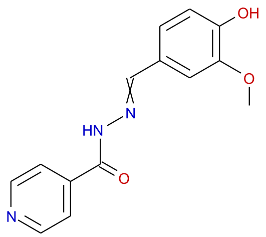	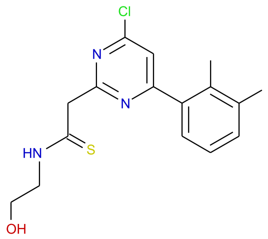	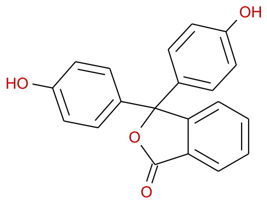
Actual Endpoint (-log C)	3.99565	3.87681	2.43468
Predicted Endpoint (-log C)	3.22211	3.77582	3.66084
Distance	0.626	0.687	0.689
Reference	CPDB	CPDB	CPDB

Model Applicability

Unknown features are fingerprint features in the query molecule, but not found or appearing too infrequently in the training set.

1. All properties and OPS components are within expected ranges.

Feature Contribution

Top features for positive contribution

Fingerprint	Bit/Smiles	Feature Structure	Score
ECFP_6	655739385	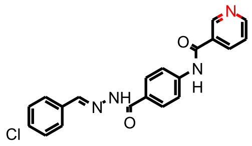 [*]:n:[*]	0.229

ECFP_6	-1925046727	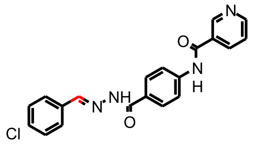 [*]C=[*]	0.145
ECFP_6	-817402818	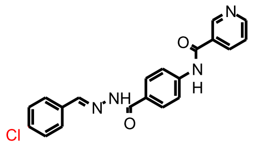 [*]Cl	0.129

Top Features for negative contribution

**Fingerprint**	**Bit/Smiles**	**Feature Structure**	**Score**
ECFP_6	1996767644	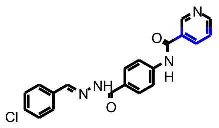 [*][c](:[*]):[cH]:[cH]:[*]	-0.251
ECFP_6	642810091	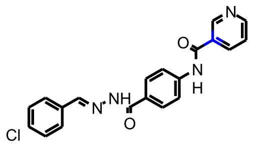 [*][c](:[*]):[*]	-0.247
ECFP_6	-182236392	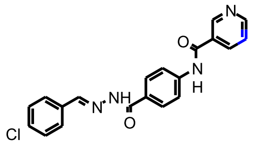 [*]:[cH]:[*]	-0.232

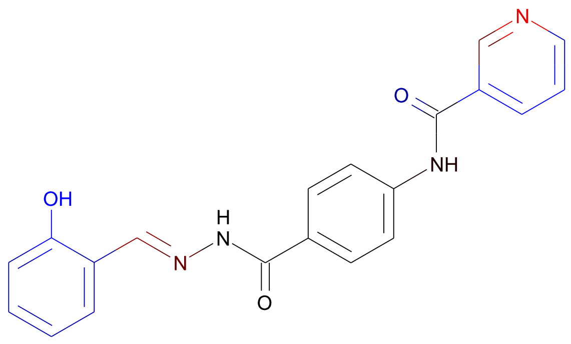

$C_{20}H_{16}N_4O_3$

Molecular Weight: 360.36604

ALogP: 2.075

Rotatable Bonds: 5

Acceptors: 5

Donors: 3

Structural Similar Compounds

Name	422	542	Ochratoxin A
Structure	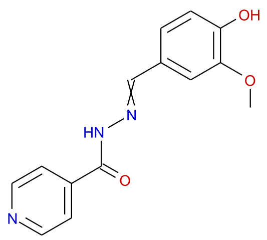	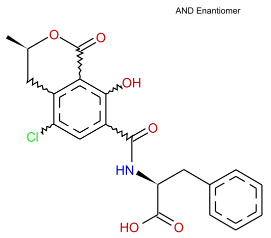	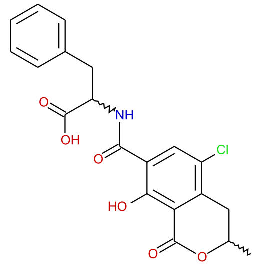
Actual Endpoint (-log C)	3.99565	4.79932	4.79932
Predicted Endpoint (-log C)	3.22211	3.6353	3.6353
Distance	0.644	0.663	0.663
Reference	CPDB	CPDB	CPDB

Model Prediction

Prediction: 50.4

Unit: mg/kg_body_weight/day

Mahalanobis Distance: 11.7

Mahalanobis Distance p-value: 7.03e-005

Mahalanobis Distance: The Mahalanobis distance (MD) is a generalization of the Euclidean distance that accounts for correlations among the X properties. It is calculated as the distance to the center of the training data. The larger the MD, the less trustworthy the prediction.
Mahalanobis Distance p-value: The p-value gives the fraction of training data with an MD greater than or equal to the one for the given sample, assuming normally distributed data. The smaller the p-value, the less trustworthy the prediciton. For highly non-normal X properties (e.g., fingerprints), the MD p-value is wildly inaccurate.

Model Applicability

Unknown features are fingerprint features in the query molecule, but not found or appearing too infrequently in the training set.

1. All properties and OPS components are within expected ranges.

Feature Contribution

Top features for positive contribution

Fingerprint	Bit/Smiles	Feature Structure	Score
ECFP_6	655739385	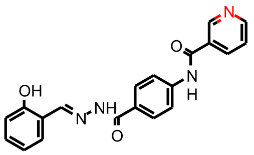 [*]:n:[*]	0.229

ECFP_6	-1925046727	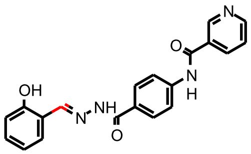 [*]C=[*]	0.145
ECFP_6	-175146122	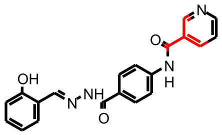 [*]C(=[*])[c](:[cH]:[*]):[cH]:[*]	0.107
**Top Features for negative contribution**			
**Fingerprint**	**Bit/Smiles**	**Feature Structure**	**Score**
ECFP_6	2019062761	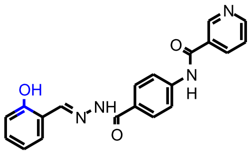 [*]:[c](:[*])O	-0.258
ECFP_6	1996767644	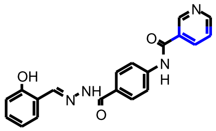 [*][c](:[*]):[cH]:[cH]:[*]	-0.251
ECFP_6	642810091	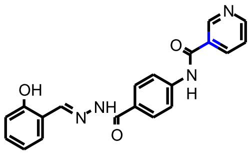 [*][c](:[*]):[*]	-0.247

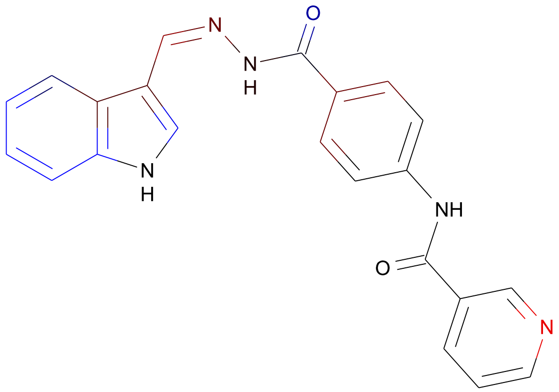

$C_{22}H_{17}N_5O_2$

Molecular Weight: 383.40268

ALogP: 2.611

Rotatable Bonds: 5

Acceptors: 4

Donors: 3

Model Prediction

Prediction: 6.02

Unit: mg/kg_body_weight/day

Mahalanobis Distance: 11.7

Mahalanobis Distance p-value: 9.87e-005

Mahalanobis Distance: The Mahalanobis distance (MD) is a generalization of the Euclidean distance that accounts for correlations among the X properties. It is calculated as the distance to the center of the training data. The larger the MD, the less trustworthy the prediction.
Mahalanobis Distance p-value: The p-value gives the fraction of training data with an MD greater than or equal to the one for the given sample, assuming normally distributed data. The smaller the p-value, the less trustworthy the prediciton. For highly non-normal X properties (e.g., fingerprints), the MD p-value is wildly inaccurate.

Structural Similar Compounds

Name	Phenolphthalein	422	542
Structure	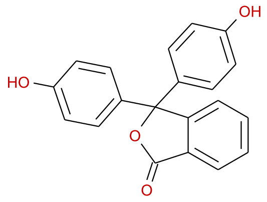	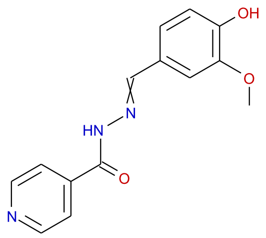	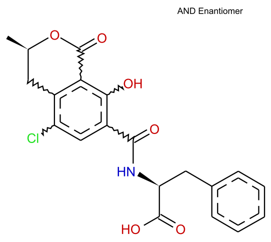
Actual Endpoint (-log C)	2.43468	3.99565	4.79932
Predicted Endpoint (-log C)	3.66084	3.22211	3.6353
Distance	0.771	0.842	0.843
Reference	CPDB	CPDB	CPDB

Model Applicability

Unknown features are fingerprint features in the query molecule, but not found or appearing too infrequently in the training set.

1. All properties and OPS components are within expected ranges.
2. Unknown ECFP_2 feature: -1020449580: [*][c]1:[*]:[*]:[nH]:c:1

Feature Contribution

Top features for positive contribution

Fingerprint	Bit/Smiles	Feature Structure	Score
ECFP_6	655739385	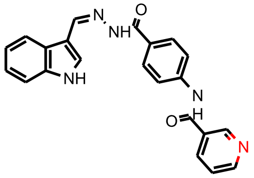 [*]:n:[*]	0.229

ECFP_6	-1925046727	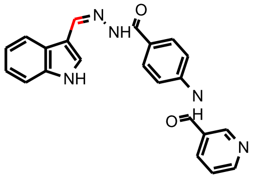 [*]C=[*]	0.145
ECFP_6	-175146122	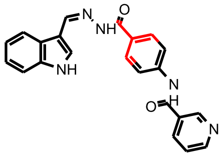 [*]C(=[*])[c](:[cH]:[*]):[cH]:[*]	0.107
**Top Features for negative contribution**			
**Fingerprint**	**Bit/Smiles**	**Feature Structure**	**Score**
ECFP_6	1996767644	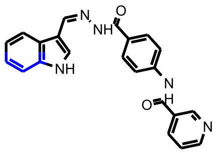 [*][c](:[*]):[cH]:[cH]:[*]	-0.251
ECFP_6	642810091	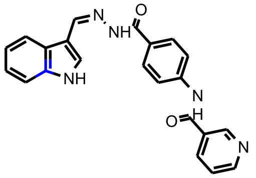 [*][c](:[*]):[*]	-0.247
ECFP_6	-182236392	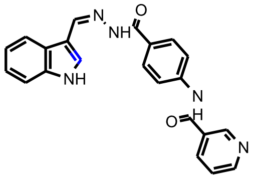 [*]:[cH]:[*]	-0.232

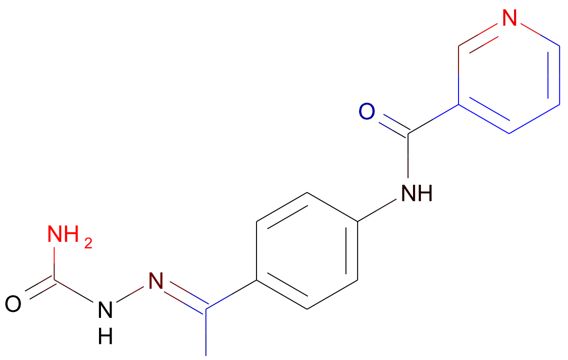

$C_{15}H_{15}N_5O_2$
Molecular Weight: 297.3119
ALogP: 0.423
Rotatable Bonds: 4
Acceptors: 4
Donors: 3

Model Prediction

Prediction: 36.9
Unit: mg/kg_body_weight/day
Mahalanobis Distance: 12
Mahalanobis Distance p-value: 1.84e-005

Mahalanobis Distance: The Mahalanobis distance (MD) is a generalization of the Euclidean distance that accounts for correlations among the X properties. It is calculated as the distance to the center of the training data. The larger the MD, the less trustworthy the prediction.
Mahalanobis Distance p-value: The p-value gives the fraction of training data with an MD greater than or equal to the one for the given sample, assuming normally distributed data. The smaller the p-value, the less trustworthy the prediciton. For highly non-normal X properties (e.g., fingerprints), the MD p-value is wildly inaccurate.

Structural Similar Compounds

Name	Sulfamethazine	2-Hydrazino-4-(p-aminophenyl) thi-azole	422
Structure	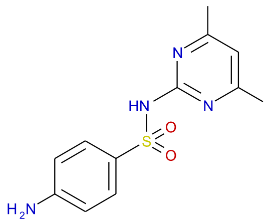	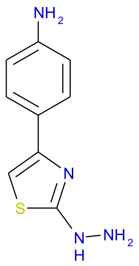	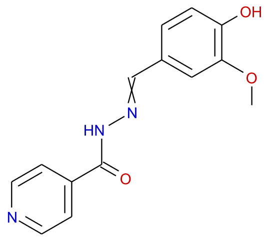
Actual Endpoint (-log C)	2.26558	4.26135	3.99565
Predicted Endpoint (-log C)	3.67808	4.32504	3.22211
Distance	0.605	0.625	0.629
Reference	CPDB	CPDB	CPDB

Model Applicability

Unknown features are fingerprint features in the query molecule, but not found or appearing too infrequently in the training set.

1. All properties and OPS components are within expected ranges.
2. Unknown ECFP_2 feature: 128986386: [*]\N=C(/C)\[c](:[*]):[*]
3. Unknown ECFP_2 feature: 560380707: [*]NN=C([*])[*]

Feature Contribution

Top features for positive contribution

Fingerprint	Bit/Smiles	Feature Structure	Score
ECFP_6	655739385	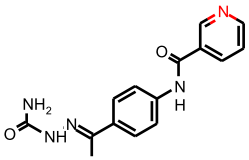 [*]:n:[*]	0.229

ECFP_6	1572579716	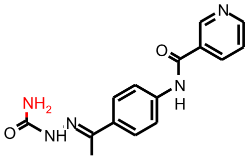 [*]N	0.225
ECFP_6	-175146122	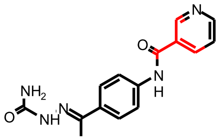 [*]C(=[*])[c](:[cH]:[*]):[cH]:[*]	0.107

Top Features for negative contribution

Fingerprint	Bit/Smiles	Feature Structure	Score
ECFP_6	1996767644	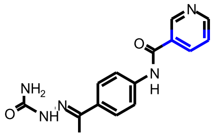 [*][c](:[*]):[cH]:[cH]:[*]	-0.251
ECFP_6	642810091	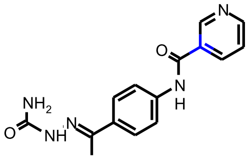 [*][c](:[*]):[*]	-0.247
ECFP_6	-182236392	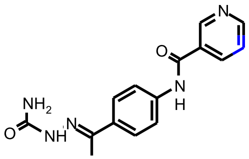 [*]:[cH]:[*]	-0.232

Sorafenib

TOPKAT_Carcinogenic_Potency_TD50_Mouse

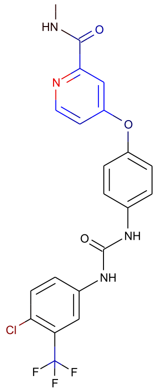

$C_{21}H_{16}ClF_3N_4O_3$

Molecular Weight: 464.82494

ALogP: 4.175

Rotatable Bonds: 6

Acceptors: 4

Donors: 3

Model Prediction

Prediction: 19.2

Unit: mg/kg_body_weight/day

Mahalanobis Distance: 12.4

Mahalanobis Distance p-value: 2.94e-006

Mahalanobis Distance: The Mahalanobis distance (MD) is a generalization of the Euclidean distance that accounts for correlations among the X properties. It is calculated as the distance to the center of the training data. The larger the MD, the less trustworthy the prediction.
Mahalanobis Distance p-value: The p-value gives the fraction of training data with an MD greater than or equal to the one for the given sample, assuming normally distributed data. The smaller the p-value, the less trustworthy the prediciton. For highly non-normal X properties (e.g., fingerprints), the MD p-value is wildly inaccurate.

Structural Similar Compounds

Name	Ochratoxin A	542	4-Chloro-6-(2,3-xylidino)-2-pyri-mi-dinylthio(N-b-hydroxy-ethyl) acetamide
Structure	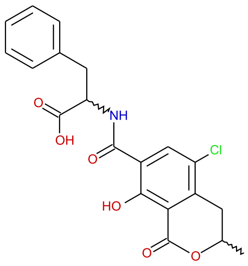	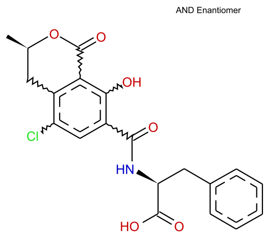	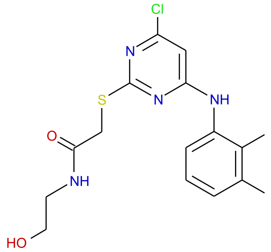
Actual Endpoint (-log C)	4.79932	4.79932	3.91517
Predicted Endpoint (-log C)	3.6353	3.6353	3.92186
Distance	0.718	0.718	0.738
Reference	CPDB	CPDB	CPDB

Model Applicability

Unknown features are fingerprint features in the query molecule, but not found or appearing too infrequently in the training set.

1. All properties and OPS components are within expected ranges.
2. Unknown ECFP_2 feature: 1413420509: [*]C(=[*])[c](:n:[*]):c:[*]
3. Unknown ECFP_2 feature: 1338334141: [*]C(=[*])NC

Feature Contribution

Top features for positive contribution

Fingerprint	Bit/Smiles	Feature Structure	Score
ECFP_6	655739385	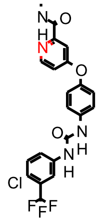 [*]:n:[*]	0.229

ECFP_6	-817402818	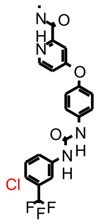 [*]Cl	0.129
ECFP_6	-176455838	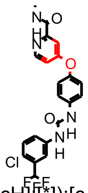 [*]O[c](:[cH]:[*]):[cH]:[*]	0.0818

Top Features for negative contribution

Fingerprint	Bit/Smiles	Feature Structure	Score
ECFP_6	1996767644	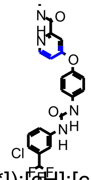 [*][c](:[*]):[cH]:[cH]:[*]	-0.251
ECFP_6	642810091	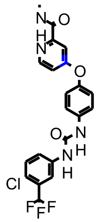 [*][c](:[*]):[*]	-0.247
ECFP_6	-182236392	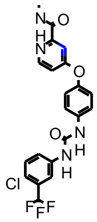 [*]:[cH]:[*]	-0.232

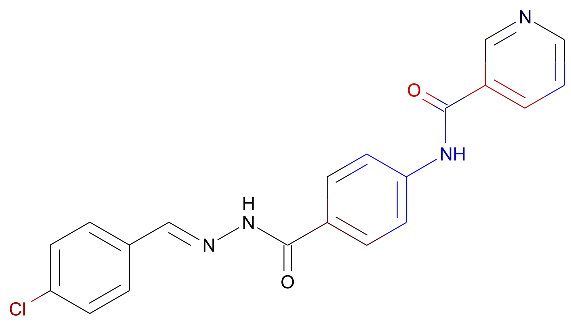

$C_{20}H_{15}ClN_4O_2$
Molecular Weight: 378.8117
ALogP: 2.981
Rotatable Bonds: 5
Acceptors: 4
Donors: 2

Model Prediction

Prediction: 12.3
Unit: mg/kg_body_weight/day
Mahalanobis Distance: 11.9
Mahalanobis Distance p-value: 0.00067

Mahalanobis Distance: The Mahalanobis distance (MD) is a generalization of the Euclidean distance that accounts for correlations among the X properties. It is calculated as the distance to the center of the training data. The larger the MD, the less trustworthy the prediction.
Mahalanobis Distance p-value: The p-value gives the fraction of training data with an MD greater than or equal to the one for the given sample, assuming normally distributed data. The smaller the p-value, the less trustworthy the prediciton. For highly non-normal X properties (e.g., fingerprints), the MD p-value is wildly inaccurate.

Structural Similar Compounds

Name	Indomethacin	3-(Cyclopentyloxy)-N-(3,5-di-chloro-4-pyridyl)-4-methoxy-benzamide	Omeprazole
Structure	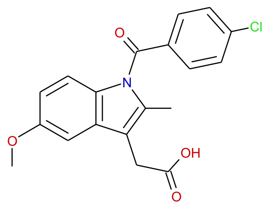	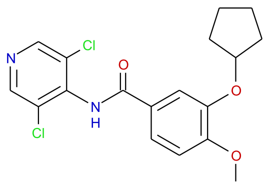	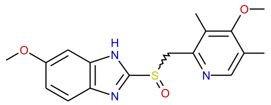
Actual Endpoint (-log C)	5.49293	5.39369	3.4628
Predicted Endpoint (-log C)	4.9569	4.27874	4.7324
Distance	0.586	0.596	0.597
Reference	CPDB	CPDB	CPDB

Model Applicability

Unknown features are fingerprint features in the query molecule, but not found or appearing too infrequently in the training set.

1. OPS PC6 out of range. Value: 6.702. Training min, max, SD, explained variance: -5.5832, 6.4847, 1.973, 0.0374.

Feature Contribution

Top features for positive contribution			
Fingerprint	Bit/Smiles	Feature Structure	Score
FCFP_6	1	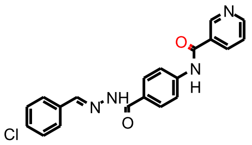 [*]=O	0.234

FCFP_6	32	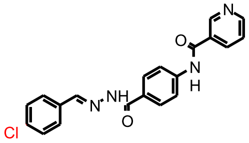 [*]Cl	0.154
FCFP_6	730557100	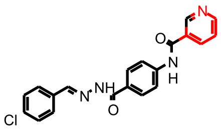 [*][c]1:[*]:n:[cH]:[cH]:[cH]:1	0.141
**Top Features for negative contribution**			
**Fingerprint**	**Bit/Smiles**	**Feature Structure**	**Score**
FCFP_6	16	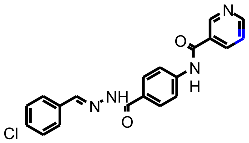 [*]:[cH]:[*]	-0.354
FCFP_6	590925877	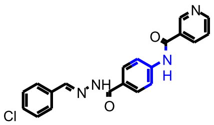 [*]N[c](:[cH]:[*]):[cH]:[*]	-0.323
FCFP_6	17	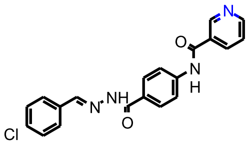 [*]:n:[*]	-0.149

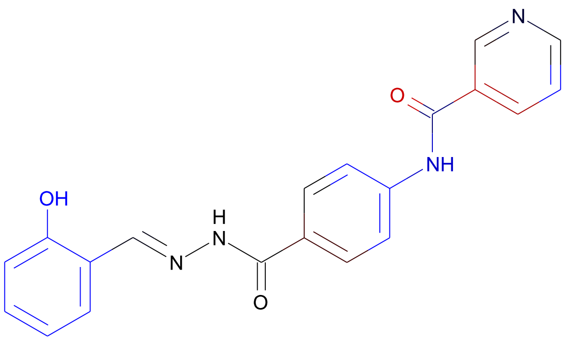

$C_{20}H_{16}N_4O_3$
Molecular Weight: 360.36604
ALogP: 2.075
Rotatable Bonds: 5
Acceptors: 5
Donors: 3

Model Prediction

Prediction: 276
Unit: mg/kg_body_weight/day
Mahalanobis Distance: 12.8
Mahalanobis Distance p-value: 1.72e-005

Mahalanobis Distance: The Mahalanobis distance (MD) is a generalization of the Euclidean distance that accounts for correlations among the X properties. It is calculated as the distance to the center of the training data. The larger the MD, the less trustworthy the prediction.
Mahalanobis Distance p-value: The p-value gives the fraction of training data with an MD greater than or equal to the one for the given sample, assuming normally distributed data. The smaller the p-value, the less trustworthy the prediciton. For highly non-normal X properties (e.g., fingerprints), the MD p-value is wildly inaccurate.

Structural Similar Compounds

Name	542	Ochratoxin A	4,4´-Sulfonylbisacetanilide
Structure	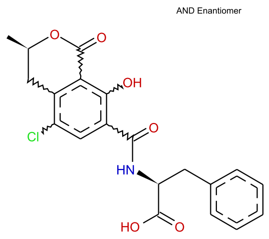	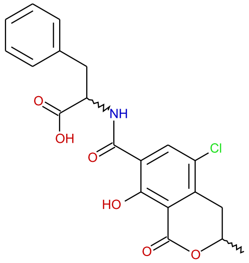	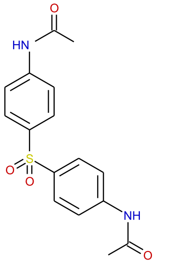
Actual Endpoint (-log C)	6.59334	6.47264	3.77655
Predicted Endpoint (-log C)	5.06501	5.06501	3.55337
Distance	0.606	0.606	0.640
Reference	CPDB	CPDB	CPDB

Model Applicability

Unknown features are fingerprint features in the query molecule, but not found or appearing too infrequently in the training set.

1. All properties and OPS components are within expected ranges.

Feature Contribution

Top features for positive contribution

Fingerprint	Bit/Smiles	Feature Structure	Score
FCFP_6	1	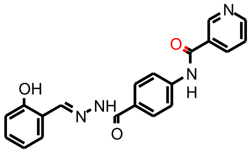 [*]=O	0.234

FCFP_6	730557100	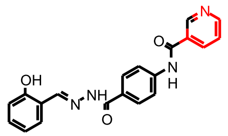 [*][c]1:[*]:n:[cH]:[cH]:[cH]:1	0.141
FCFP_6	203677720	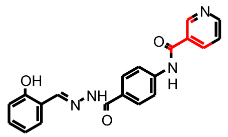 [*]C(=[*])[c](:[cH]:[*]):[cH]:[*]	0.137
**Top Features for negative contribution**			
**Fingerprint**	**Bit/Smiles**	**Feature Structure**	**Score**
FCFP_6	991735244	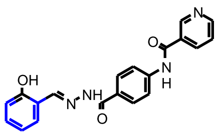 [*][c]1:[*]:[cH]:[cH]:[cH]:[cH]:1	-0.422
FCFP_6	7	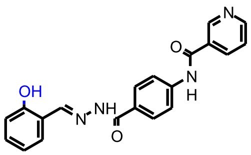 [*]O	-0.372
FCFP_6	16	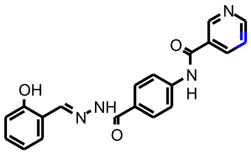 [*]:[cH]:[*]	-0.354

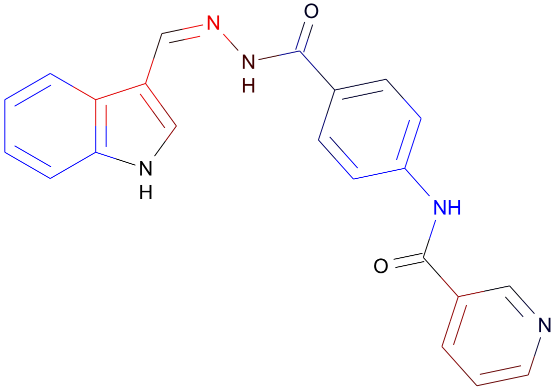

$C_{22}H_{17}N_5O_2$

Molecular Weight: 383.40268

ALogP: 2.611

Rotatable Bonds: 5

Acceptors: 4

Donors: 3

Model Prediction

Prediction: 15

Unit: mg/kg_body_weight/day

Mahalanobis Distance: 12.2

Mahalanobis Distance p-value: 0.000261

Mahalanobis Distance: The Mahalanobis distance (MD) is a generalization of the Euclidean distance that accounts for correlations among the X properties. It is calculated as the distance to the center of the training data. The larger the MD, the less trustworthy the prediction.
Mahalanobis Distance p-value: The p-value gives the fraction of training data with an MD greater than or equal to the one for the given sample, assuming normally distributed data. The smaller the p-value, the less trustworthy the prediciton. For highly non-normal X properties (e.g., fingerprints), the MD p-value is wildly inaccurate.

Structural Similar Compounds

Name	Fluvastatin	913	796
Structure	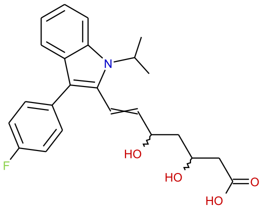	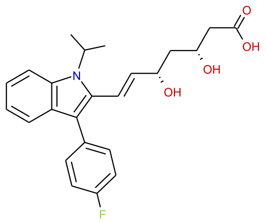	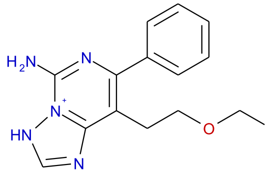
Actual Endpoint (-log C)	3.51742	3.51742	2.71505
Predicted Endpoint (-log C)	5.41573	5.41573	4.45918
Distance	0.711	0.711	0.724
Reference	CPDB	CPDB	CPDB

Model Applicability

Unknown features are fingerprint features in the query molecule, but not found or appearing too infrequently in the training set.

1. OPS PC6 out of range. Value: 6.6351. Training min, max, SD, explained variance: -5.5832, 6.4847, 1.973, 0.0374.

Feature Contribution

Top features for positive contribution			
**Fingerprint**	**Bit/Smiles**	**Feature Structure**	**Score**
FCFP_6	1	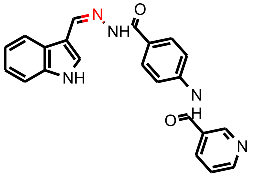 [*]=O	0.234

FCFP_6	730557100	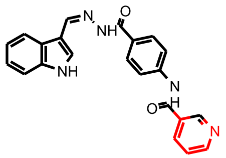 [*][c]1:[*]:n:[cH]:[cH]:[cH]:1	0.141
FCFP_6	203677720	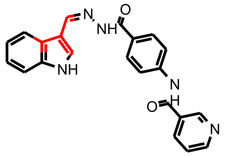 [*]C(=[*])[c](:[cH]:[*]):[cH]:[*]	0.137
**Top Features for negative contribution**			
**Fingerprint**	**Bit/Smiles**	**Feature Structure**	**Score**
FCFP_6	991735244	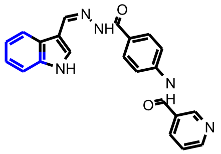 [*][c]1:[*]:[cH]:[cH]:[cH]:[cH]:1	-0.422
FCFP_6	16	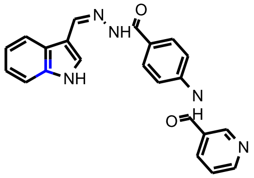 [*]:[cH]:[*]	-0.354
FCFP_6	590925877	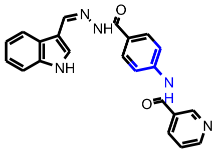 [*]N[c](:[cH]:[*]):[cH]:[*]	-0.323

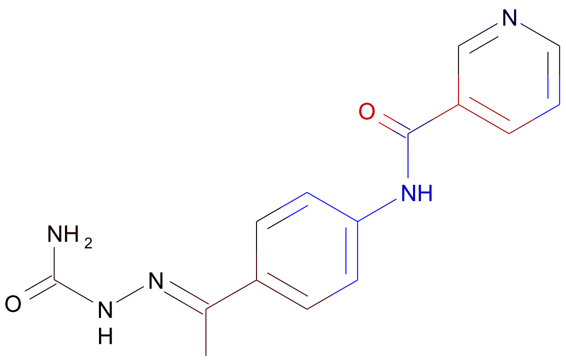

$C_{15}H_{15}N_5O_2$

Molecular Weight: 297.3119

ALogP: 0.423

Rotatable Bonds: 4

Acceptors: 4

Donors: 3

Model Prediction

Prediction: 54.8

Unit: mg/kg_body_weight/day

Mahalanobis Distance: 11.6

Mahalanobis Distance p-value: 0.00285

Mahalanobis Distance: The Mahalanobis distance (MD) is a generalization of the Euclidean distance that accounts for correlations among the X properties. It is calculated as the distance to the center of the training data. The larger the MD, the less trustworthy the prediction.
Mahalanobis Distance p-value: The p-value gives the fraction of training data with an MD greater than or equal to the one for the given sample, assuming normally distributed data. The smaller the p-value, the less trustworthy the prediciton. For highly non-normal X properties (e.g., fingerprints), the MD p-value is wildly inaccurate.

Structural Similar Compounds

Name	4,4´-Sulfonylbisacetanilide	Dapsone	1,2-Dihydro-2-(5-nitro-2-thi-enyl) quinazolin-4(3H)-one
Structure	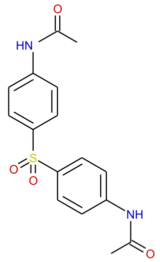	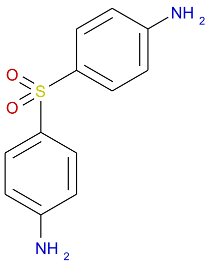	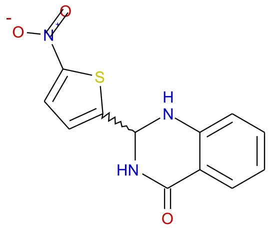
Actual Endpoint (-log C)	3.77655	4.04473	5.25509
Predicted Endpoint (-log C)	3.55337	4.05717	3.89291
Distance	0.523	0.645	0.647
Reference	CPDB	CPDB	CPDB

Model Applicability

Unknown features are fingerprint features in the query molecule, but not found or appearing too infrequently in the training set.

1. All properties and OPS components are within expected ranges.

Feature Contribution

Top features for positive contribution

Fingerprint	Bit/Smiles	Feature Structure	Score
FCFP_6	1	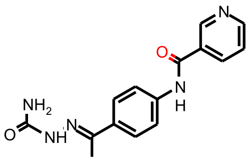 [*]=O	0.234

FCFP_6	730557100	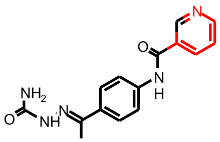 [*][c]1:[*]:n:[cH]:[c H]:[cH]:1	0.141
FCFP_6	203677720	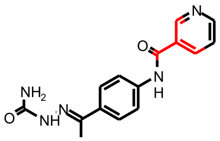 [*]C(=[*])[c](:[cH]:[*]):[cH]:[*]	0.137

Top Features for negative contribution

Fingerprint	Bit/Smiles	Feature Structure	Score
FCFP_6	16	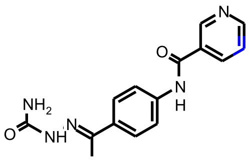 [*]:[cH]:[*]	-0.354
FCFP_6	590925877	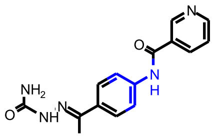 [*]N[c](:[cH]:[*]):[c H]:[*]	-0.323
FCFP_6	17	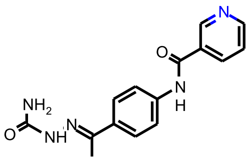 [*]:n:[*]	-0.149

Sorafenib

TOPKAT_Carcinogenic_Potency_TD50_Rat

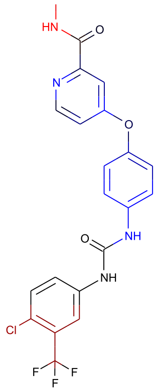

$C_{21}H_{16}ClF_3N_4O_3$

Molecular Weight: 464.82494

ALogP: 4.175

Rotatable Bonds: 6

Acceptors: 4

Donors: 3

Model Prediction

Prediction: 14.2

Unit: mg/kg_body_weight/day

Mahalanobis Distance: 20.4

Mahalanobis Distance p-value: 9.56e-031

Mahalanobis Distance: The Mahalanobis distance (MD) is a generalization of the Euclidean distance that accounts for correlations among the X properties. It is calculated as the distance to the center of the training data. The larger the MD, the less trustworthy the prediction.

Mahalanobis Distance p-value: The p-value gives the fraction of training data with an MD greater than or equal to the one for the given sample, assuming normally distributed data. The smaller the p-value, the less trustworthy the prediciton. For highly non-normal X properties (e.g., fingerprints), the MD p-value is wildly inaccurate.

Structural Similar Compounds

Name	Fluvastatin	913	Ochratoxin A
Structure	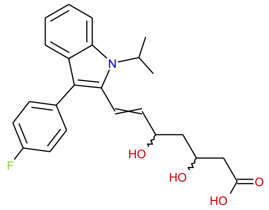	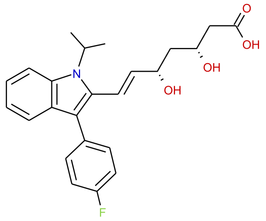	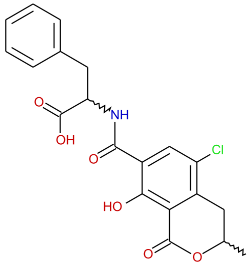
Actual Endpoint (-log C)	3.51742	3.51742	6.47264
Predicted Endpoint (-log C)	5.41573	5.41573	5.06501
Distance	0.597	0.597	0.666
Reference	CPDB	CPDB	CPDB

Model Applicability

Unknown features are fingerprint features in the query molecule, but not found or appearing too infrequently in the training set.

1. All properties and OPS components are within expected ranges.
2. Unknown FCFP_2 feature: -1029533685: [*]:[c](:[*])C(F)(F)F

Feature Contribution

Top features for positive contribution

Fingerprint	Bit/Smiles	Feature Structure	Score
FCFP_6	1	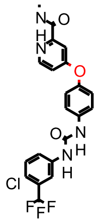 [*]=O	0.234

FCFP_6	-885550502	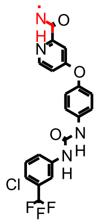 [*]C(=[*])NC	0.229
FCFP_6	32	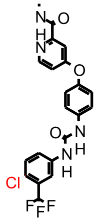 [*]Cl	0.154
**Top Features for negative contribution**			
**Fingerprint**	**Bit/Smiles**	**Feature Structure**	**Score**
FCFP_6	16	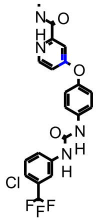 [*]:[cH]:[*]	-0.354
FCFP_6	590925877	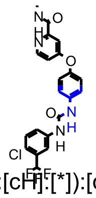 [*]N[c](:[cH]:[*]):[cH]:[*]	-0.323
FCFP_6	1674451008	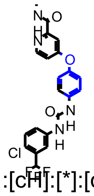 [*]O[c]1:[cH]:[*]:[c]([*]):[cH]:[cH]:1	-0.233

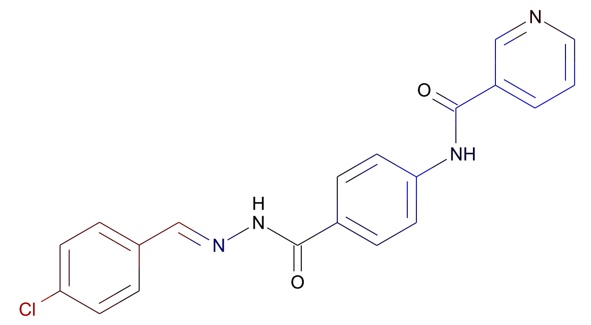

$C_{20}H_{15}ClN_4O_2$
Molecular Weight: 378.8117
ALogP: 2.981
Rotatable Bonds: 5
Acceptors: 4
Donors: 2

Model Prediction

Prediction: 0.0958
Unit: g/kg_body_weight
Mahalanobis Distance: 28.8
Mahalanobis Distance p-value: 1.8e-022

Mahalanobis Distance: The Mahalanobis distance (MD) is a generalization of the Euclidean distance that accounts for correlations among the X properties. It is calculated as the distance to the center of the training data. The larger the MD, the less trustworthy the prediction.
Mahalanobis Distance p-value: The p-value gives the fraction of training data with an MD greater than or equal to the one for the given sample, assuming normally distributed data. The smaller the p-value, the less trustworthy the prediciton. For highly non-normal X properties (e.g., fingerprints), the MD p-value is wildly inaccurate.

Structural Similar Compounds

Name	HC BLUE 1	ISOXABEN	FLUCONAZOLE
Structure	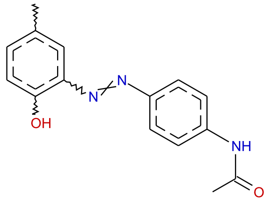	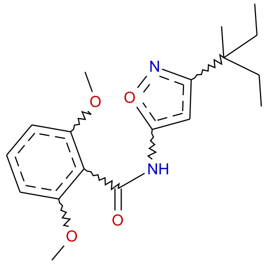	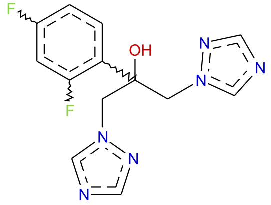
Actual Endpoint (-log C)	3.0323	3.81665	5.08818
Predicted Endpoint (-log C)	2.7171	4.42315	4.97396
Distance	0.656	0.664	0.674
Reference	NTP REPORT # 222	EPA COVER SHEET 0339;881201;(1)	NDA-19949

Model Applicability

Unknown features are fingerprint features in the query molecule, but not found or appearing too infrequently in the training set.

1. All properties and OPS components are within expected ranges.
2. Unknown ECFP_6 feature: 1997021792: [*]:[cH]:[cH]:[cH]:[*]
3. Unknown ECFP_6 feature: 1996163143: [*]:[cH]:[cH]:n:[*]
4. Unknown ECFP_6 feature: -677055651: [*]:[cH]:n:[cH]:[*]
5. Unknown ECFP_6 feature: -709633021: [*][c](:[*]):[cH]:n:[*]
6. Unknown ECFP_6 feature: -175146122: [*]C(=[*])[c](:[cH]:[*]):[cH]:[*]
7. Unknown ECFP_6 feature: 1430169877: [*]NC(=O)[c](:[*]):[*]
8. Unknown ECFP_6 feature: -177077903: [*]N[c](:[cH]:[*]):[cH]:[*]
9. Unknown ECFP_6 feature: 544048674: [*]C(=[*])NN=[*]
10. Unknown ECFP_6 feature: 1814278164: [*]N\N=C\[*]
11. Unknown ECFP_6 feature: -1832102709: [*]\N=C\[c](:[*]):[*]
12. Unknown ECFP_6 feature: -176483725: [*]=C[c](:[cH]:[*]):[cH]:[*]
13. Unknown ECFP_6 feature: -176494269: [*]:[cH]:[c](Cl):[cH]:[*]
14. Unknown ECFP_6 feature: 99947387: [*]:[c](:[*])Cl

Feature Contribution

Top features for positive contribution			
**Fingerprint**	**Bit/Smiles**	**Feature Structure**	**Score**

FCFP_6	32	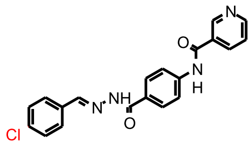 [*]Cl	0.101
FCFP_6	3	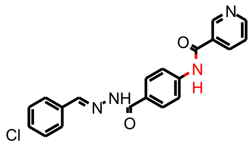 [*]N[*]	0.0924
ECFP_6	-1925046727	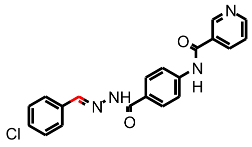 [*]C=[*]	0.0915

Top Features for negative contribution

**Fingerprint**	**Bit/Smiles**	**Feature Structure**	**Score**
FCFP_6	1	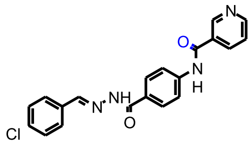 [*]=O	-0.102
ECFP_6	-1087070950	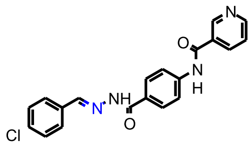 [*]N=[*]	-0.102

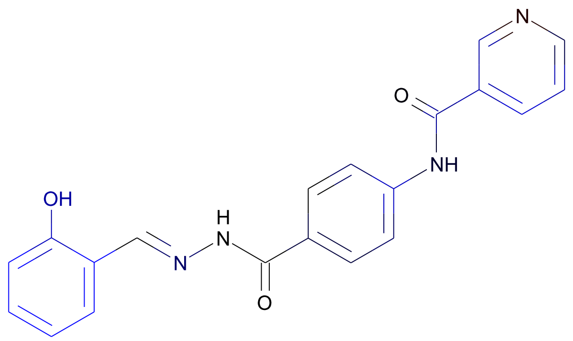

$C_{20}H_{16}N_4O_3$
Molecular Weight: 360.36604
ALogP: 2.075
Rotatable Bonds: 5
Acceptors: 5
Donors: 3

Model Prediction

Prediction: 0.481
Unit: g/kg_body_weight
Mahalanobis Distance: 27.7
Mahalanobis Distance p-value: 1.67e-020

Mahalanobis Distance: The Mahalanobis distance (MD) is a generalization of the Euclidean distance that accounts for correlations among the X properties. It is calculated as the distance to the center of the training data. The larger the MD, the less trustworthy the prediction.
Mahalanobis Distance p-value: The p-value gives the fraction of training data with an MD greater than or equal to the one for the given sample, assuming normally distributed data. The smaller the p-value, the less trustworthy the prediciton. For highly non-normal X properties (e.g., fingerprints), the MD p-value is wildly inaccurate.

Structural Similar Compounds

Name	PIROXICAM	FUROSEMIDE	CHLORSULFURON
Structure	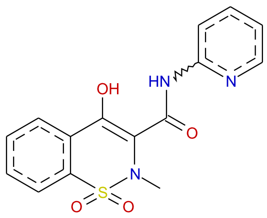	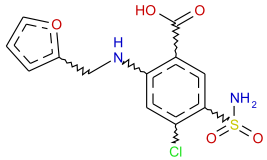	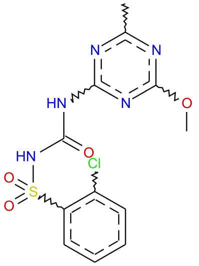
Actual Endpoint (-log C)	5.52028	4.27645	4.15566
Predicted Endpoint (-log C)	4.06087	4.40005	3.79771
Distance	0.670	0.676	0.677
Reference	NDA-18147	NTP REPORT # 356	EPA COVER SHEET 0027;880301;(1)

Model Applicability

Unknown features are fingerprint features in the query molecule, but not found or appearing too infrequently in the training set.

1. All properties and OPS components are within expected ranges.
2. Unknown ECFP_6 feature: 1997021792: [*]:[cH]:[cH]:[cH]:[*]
3. Unknown ECFP_6 feature: 1996163143: [*]:[cH]:[cH]:n:[*]
4. Unknown ECFP_6 feature: -677055651: [*]:[cH]:n:[cH]:[*]
5. Unknown ECFP_6 feature: -709633021: [*][c](:[*]):[cH]:n:[*]
6. Unknown ECFP_6 feature: -175146122: [*]C(=[*])[c](:[cH]:[*]):[cH]:[*]
7. Unknown ECFP_6 feature: 1430169877: [*]NC(=O)[c](:[*]):[*]
8. Unknown ECFP_6 feature: -177077903: [*]N[c](:[cH]:[*]):[cH]:[*]
9. Unknown ECFP_6 feature: 544048674: [*]C(=[*])NN=[*]
10. Unknown ECFP_6 feature: 1814278164: [*]N\N=C\[*]
11. Unknown ECFP_6 feature: -1832102709: [*]\N=C\[c](:[*]):[*]
12. Unknown ECFP_6 feature: 1335702447: [*][c](:[*]):[c](C=[*]):[cH]:[*]
13. Unknown ECFP_6 feature: 2019062761: [*]:[c](:[*])O

Feature Contribution

Top features for positive contribution			
Fingerprint	Bit/Smiles	Feature Structure	Score

FCFP_6	3	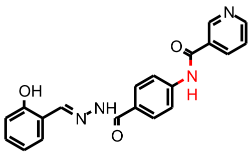 [*]N[*]	0.0924
ECFP_6	-1925046727	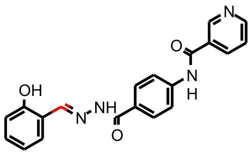 [*]C=[*]	0.0915
ECFP_6	2099970318	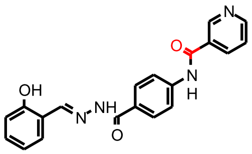 [*]C(=O)[*]	0.0766
**Top Features for negative contribution**			
**Fingerprint**	**Bit/Smiles**	**Feature Structure**	**Score**
FCFP_6	991735244	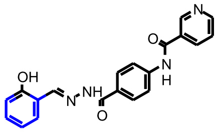 [*][c]1:[*]:[cH]:[cH]:[cH]:[cH]:1	-0.134
ECFP_6	1564392544	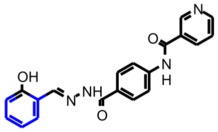 [*][c]1:[*]:[cH]:[cH]:[cH]:[cH]:1	-0.133

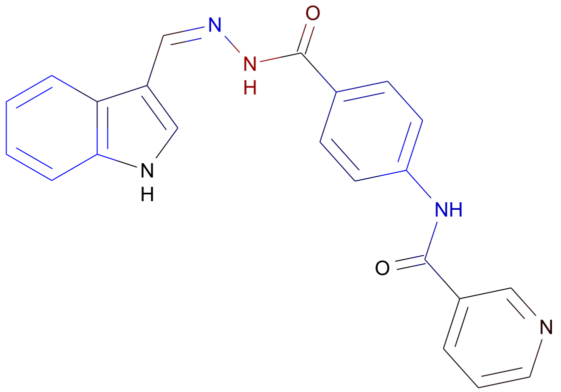

$C_{22}H_{17}N_5O_2$

Molecular Weight: 383.40268

ALogP: 2.611

Rotatable Bonds: 5

Acceptors: 4

Donors: 3

Model Prediction

Prediction: 0.316

Unit: g/kg_body_weight

Mahalanobis Distance: 32.1

Mahalanobis Distance p-value: 2.01e-028

Mahalanobis Distance: The Mahalanobis distance (MD) is a generalization of the Euclidean distance that accounts for correlations among the X properties. It is calculated as the distance to the center of the training data. The larger the MD, the less trustworthy the prediction.
Mahalanobis Distance p-value: The p-value gives the fraction of training data with an MD greater than or equal to the one for the given sample, assuming normally distributed data. The smaller the p-value, the less trustworthy the prediciton. For highly non-normal X properties (e.g., fingerprints), the MD p-value is wildly inaccurate.

Structural Similar Compounds

Name	D & C RED 9	FLUCONAZOLE	C.I. PIGMENT RED 3
Structure	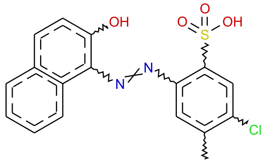	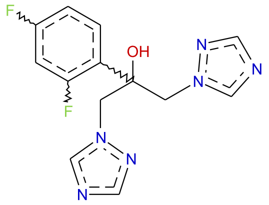	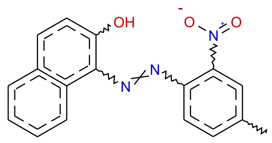
Actual Endpoint (-log C)	3.87715	5.08818	3.0252
Predicted Endpoint (-log C)	3.6546	4.97396	3.34768
Distance	0.781	0.812	0.827
Reference	NTP REPORT # 225	NDA-19949	NTP REPORT # 407

Model Applicability

Unknown features are fingerprint features in the query molecule, but not found or appearing too infrequently in the training set.

1. All properties and OPS components are within expected ranges.
2. Unknown ECFP_6 feature: -152683720: [*]:[nH]:[*]
3. Unknown ECFP_6 feature: 1099224616: [*]:[cH]:[c]1:[nH]:[*]:[*]:[c]:1:[*]
4. Unknown ECFP_6 feature: 1333660716: [*][c]1:[*]:[*]:[c](:[*]):[c]:1:[cH]:[*]
5. Unknown ECFP_6 feature: 1335702447: [*][c](:[*]):[c](C=[*]):[cH]:[*]
6. Unknown ECFP_6 feature: -1020449580: [*][c]1:[*]:[*]:[nH]:[cH]:1
7. Unknown ECFP_6 feature: -953984246: [*]:[c]1:[*]:[*]:[cH]:[nH]:1
8. Unknown ECFP_6 feature: 1997021792: [*]:[cH]:[cH]:[cH]:[*]
9. Unknown ECFP_6 feature: -1832102709: [*]\N=C\[c](:[*]):[*]
10. Unknown ECFP_6 feature: 1814278164: [*]N\N=C\[*]
11. Unknown ECFP_6 feature: 544048674: [*]C(=[*])NN=[*]
12. Unknown ECFP_6 feature: 1430169877: [*]NC(=O)[c](:[*]):[*]
13. Unknown ECFP_6 feature: -175146122: [*]C(=[*])[c](:[cH]:[*]):[cH]:[*]
14. Unknown ECFP_6 feature: -177077903: [*]N[c](:[cH]:[*]):[cH]:[*]
15. Unknown ECFP_6 feature: 1996163143: [*]:[cH]:[cH]:n:[*]
16. Unknown ECFP_6 feature: -677055651: [*]:[cH]:n:[cH]:[*]
17. Unknown ECFP_6 feature: -709633021: [*][c](:[*]):[cH]:n:[*]

Feature Contribution

Top features for positive contribution			
**Fingerprint**	**Bit/Smiles**	**Feature Structure**	**Score**
FCFP_6	3	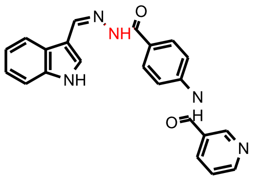 [*]N[*]	0.0924
ECFP_6	-1925046727	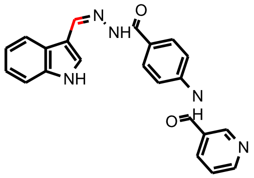 [*]C=[*]	0.0915
ECFP_6	2099970318	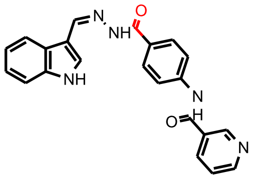 [*]C(=O)[*]	0.0766

Top Features for negative contribution			
**Fingerprint**	**Bit/Smiles**	**Feature Structure**	**Score**
FCFP_6	991735244	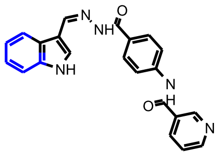 [*][c]1:[*]:[cH]:[cH]:[cH]:[cH]:1	-0.134

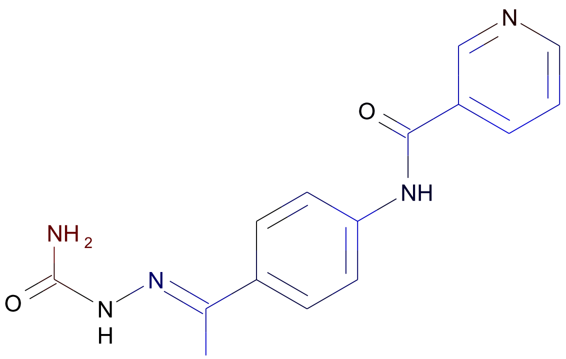

$C_{15}H_{15}N_5O_2$
Molecular Weight: 297.3119
ALogP: 0.423
Rotatable Bonds: 4
Acceptors: 4
Donors: 3

Model Prediction

Prediction: 0.23
Unit: g/kg_body_weight
Mahalanobis Distance: 27.8
Mahalanobis Distance p-value: 1.37e-020

Mahalanobis Distance: The Mahalanobis distance (MD) is a generalization of the Euclidean distance that accounts for correlations among the X properties. It is calculated as the distance to the center of the training data. The larger the MD, the less trustworthy the prediction.
Mahalanobis Distance p-value: The p-value gives the fraction of training data with an MD greater than or equal to the one for the given sample, assuming normally distributed data. The smaller the p-value, the less trustworthy the prediciton. For highly non-normal X properties (e.g., fingerprints), the MD p-value is wildly inaccurate.

Structural Similar Compounds

Name	PIROXICAM	FUROSEMIDE	DAPSONE
Structure	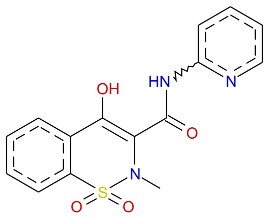	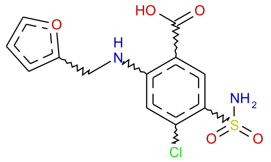	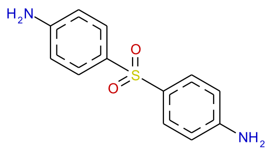
Actual Endpoint (-log C)	5.52028	4.27645	3.6168
Predicted Endpoint (-log C)	4.06087	4.40005	3.43657
Distance	0.558	0.582	0.625
Reference	NDA-18147	NTP REPORT # 356	NTP 20 47

Model Applicability

Unknown features are fingerprint features in the query molecule, but not found or appearing too infrequently in the training set.

1. All properties and OPS components are within expected ranges.
2. Unknown ECFP_6 feature: 1997021792: [*]:[cH]:[cH]:[cH]:[*]
3. Unknown ECFP_6 feature: 1996163143: [*]:[cH]:[cH]:n:[*]
4. Unknown ECFP_6 feature: -677055651: [*]:[cH]:n:[cH]:[*]
5. Unknown ECFP_6 feature: -709633021: [*][c](:[*]):[cH]:n:[*]
6. Unknown ECFP_6 feature: -175146122: [*]C(=[*])[c](:[cH]:[*]):[cH]:[*]
7. Unknown ECFP_6 feature: 1430169877: [*]NC(=O)[c](:[*]):[*]
8. Unknown ECFP_6 feature: -177077903: [*]N[c](:[cH]:[*]):[cH]:[*]
9. Unknown ECFP_6 feature: 128986386: [*]\N=C(/C)\[c](:[*]):[*]
10. Unknown ECFP_6 feature: 560380707: [*]NN=C([*])[*]
11. Unknown ECFP_6 feature: 544048674: [*]C(=[*])NN=[*]
12. Unknown ECFP_6 feature: 432952415: [*]NC(=O)N
13. Unknown ECFP_6 feature: -932108170: [*]C(=[*])N

Feature Contribution

Top features for positive contribution

Fingerprint	Bit/Smiles	Feature Structure	Score

FCFP_6	3	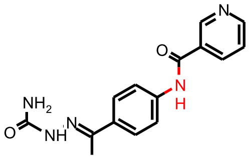 [*]N[*]	0.0924
ECFP_6	2099970318	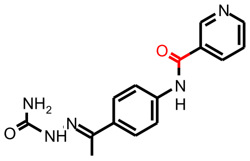 [*]C(=O)[*]	0.0766
ECFP_6	1572579716	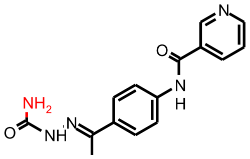 [*]N	0.0576
**Top Features for negative contribution**			
**Fingerprint**	**Bit/Smiles**	**Feature Structure**	**Score**
FCFP_6	1	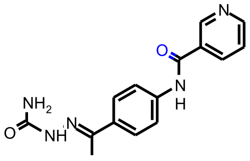 [*]=O	-0.102
ECFP_6	-1087070950	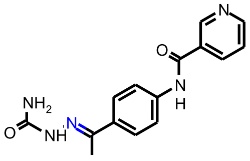 [*]N=[*]	-0.102

Sorafenib

TOPKAT_Chronic_LOAEL

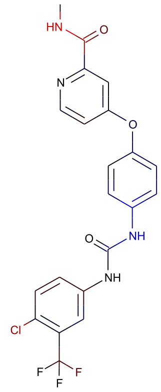

$C_{21}H_{16}ClF_3N_4O_3$

Molecular Weight: 464.82494

ALogP: 4.175

Rotatable Bonds: 6

Acceptors: 4

Donors: 3

Model Prediction

Prediction: 0.00483

Unit: g/kg_body_weight

Mahalanobis Distance: 30

Mahalanobis Distance p-value: 1.21e-024

Mahalanobis Distance: The Mahalanobis distance (MD) is a generalization of the Euclidean distance that accounts for correlations among the X properties. It is calculated as the distance to the center of the training data. The larger the MD, the less trustworthy the prediction.

Mahalanobis Distance p-value: The p-value gives the fraction of training data with an MD greater than or equal to the one for the given sample, assuming normally distributed data. The smaller the p-value, the less trustworthy the prediciton. For highly non-normal X properties (e.g., fingerprints), the MD p-value is wildly inaccurate.

Structural Similar Compounds

Name	GLYBURIDE	D & C RED 9	SODIUM ACIFLUORFEN
Structure	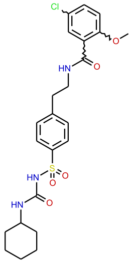	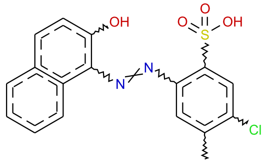	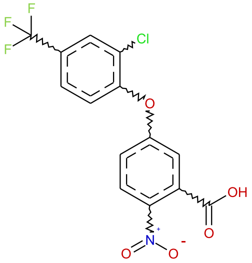
Actual Endpoint (-log C)	4.21661	3.87715	4.16036
Predicted Endpoint (-log C)	4.21035	3.6546	4.65915
Distance	0.636	0.722	0.736
Reference	UPJ-26452	NTP REPORT # 225	EPA COVER SHEET 0192;891101;(1)

Model Applicability

Unknown features are fingerprint features in the query molecule, but not found or appearing too infrequently in the training set.

1. All properties and OPS components are within expected ranges.
2. Unknown ECFP_6 feature: -1046436026: [*]F
3. Unknown ECFP_6 feature: 1305253718: [*]:[c](:[*])O[c](:[*]):[*]
4. Unknown ECFP_6 feature: 1413420509: [*]C(=[*])[c](:[cH]:[*]):n:[*]
5. Unknown ECFP_6 feature: -677309799: [*][c](:[*]):n:[cH]:[*]
6. Unknown ECFP_6 feature: 1996163143: [*]:[cH]:[cH]:n:[*]
7. Unknown ECFP_6 feature: 1430169877: [*]NC(=O)[c](:[*]):[*]
8. Unknown ECFP_6 feature: 1338334141: [*]C(=[*])NC
9. Unknown ECFP_6 feature: 864287155: [*]NC
10. Unknown ECFP_6 feature: -177077903: [*]N[c](:[cH]:[*]):[cH]:[*]
11. Unknown ECFP_6 feature: -649580166: [*]NC(=O)N[*]
12. Unknown ECFP_6 feature: 1336678434: [*][c](:[*]):[c](:[cH]:[*])C([*])([*])[*]
13. Unknown ECFP_6 feature: 99947387: [*]:[c](:[*])Cl
14. Unknown ECFP_6 feature: -1952889961: [*]:[c](:[*])C(F)(F)F
15. Unknown ECFP_6 feature: 226796801: [*]C([*])([*])F

Feature Contribution

Top features for positive contribution

Fingerprint	Bit/Smiles	Feature Structure	Score
ECFP_6	-176455838	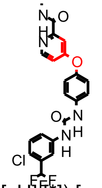 [*]O[c](:[cH]:[*]):[cH]:[*]	0.106
FCFP_6	32	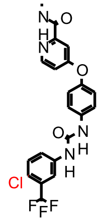 [*]Cl	0.101
FCFP_6	3	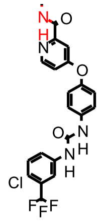 [*]N[*]	0.0924

Top Features for negative contribution

Fingerprint	Bit/Smiles	Feature Structure	Score
FCFP_6	1	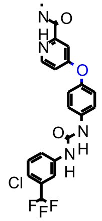 [*]=O	-0.102

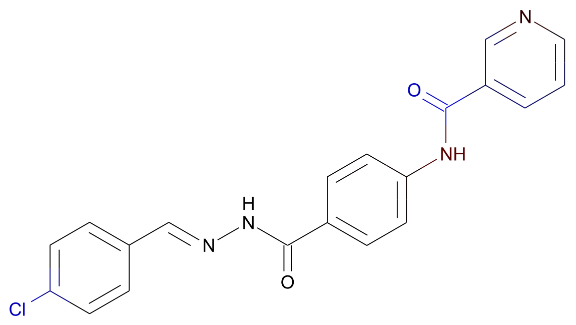

$C_{20}H_{15}ClN_4O_2$

Molecular Weight: 378.8117

ALogP: 2.981

Rotatable Bonds: 5

Acceptors: 4

Donors: 2

Structural Similar Compounds

Name	PHENOLPHTHALEIN	OXAZEPAM	1-AMINO-2,4-DIBROMOANTHRAQUINONE
Structure	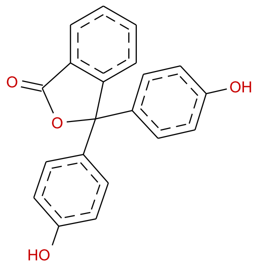	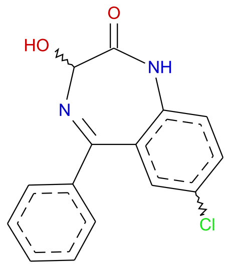	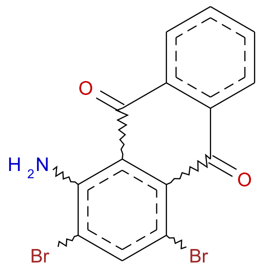
Actual Endpoint (-log C)	2.20184	3.05262	2.82966
Predicted Endpoint (-log C)	2.8857	3.13073	3.92444
Distance	0.607	0.618	0.639
Reference	NCI/NTP TR-465	NCI/NTP TR-468	NCI/NTP TR-383

Model Prediction

Prediction: 0.154

Unit: g/kg_body_weight

Mahalanobis Distance: 8.03

Mahalanobis Distance p-value: 0.0226

Mahalanobis Distance: The Mahalanobis distance (MD) is a generalization of the Euclidean distance that accounts for correlations among the X properties. It is calculated as the distance to the center of the training data. The larger the MD, the less trustworthy the prediction.

Mahalanobis Distance p-value: The p-value gives the fraction of training data with an MD greater than or equal to the one for the given sample, assuming normally distributed data. The smaller the p-value, the less trustworthy the prediciton. For highly non-normal X properties (e.g., fingerprints), the MD p-value is wildly inaccurate.

Model Applicability

Unknown features are fingerprint features in the query molecule, but not found or appearing too infrequently in the training set.

1. All properties and OPS components are within expected ranges.

Feature Contribution

Top features for positive contribution

Fingerprint	Bit/Smiles	Feature Structure	Score
FCFP_2	3	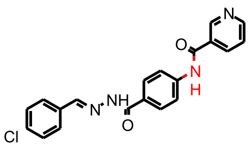 [*]N[*]	0.0737

FCFP_2	17	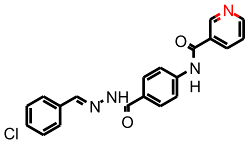 [*]:n:[*]	0.0441
FCFP_2	590925877	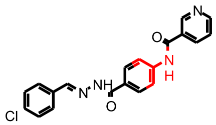 [*]N[c](:[cH]:[*]):[cH]:[*]	0.00762

Top Features for negative contribution

Fingerprint	Bit/Smiles	Feature Structure	Score
FCFP_2	71476542	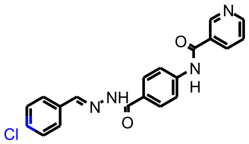 [*]:[c](:[*])Cl	-0.134
FCFP_2	1872154524	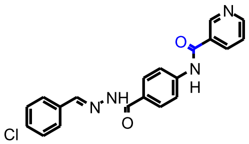 [*]C(=O)[*]	-0.105
FCFP_2	203677720	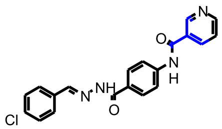 [*]C(=[*])[c](:[cH]:[*]):[cH]:[*]	-0.0829

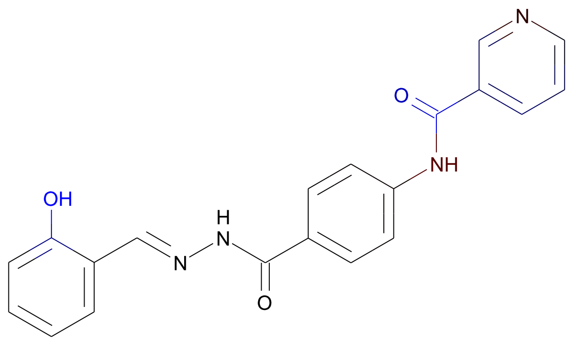

$C_{20}H_{16}N_4O_3$
Molecular Weight: 360.36604
ALogP: 2.075
Rotatable Bonds: 5
Acceptors: 5
Donors: 3

Model Prediction

Prediction: 0.414
Unit: g/kg_body_weight
Mahalanobis Distance: 7.93
Mahalanobis Distance p-value: 0.0293

Mahalanobis Distance: The Mahalanobis distance (MD) is a generalization of the Euclidean distance that accounts for correlations among the X properties. It is calculated as the distance to the center of the training data. The larger the MD, the less trustworthy the prediction.
Mahalanobis Distance p-value: The p-value gives the fraction of training data with an MD greater than or equal to the one for the given sample, assuming normally distributed data. The smaller the p-value, the less trustworthy the prediciton. For highly non-normal X properties (e.g., fingerprints), the MD p-value is wildly inaccurate.

Structural Similar Compounds

Name	FUROSEMIDE	SALICYLAZOSULFAPYRIDINE	DISPERSE YELLOW 3
Structure	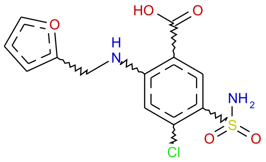	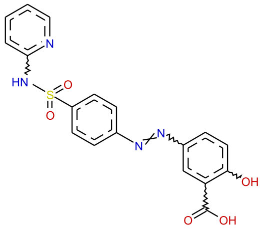	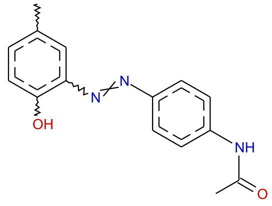
Actual Endpoint (-log C)	4.04236	3.375	2.77703
Predicted Endpoint (-log C)	2.8614	2.80292	2.80195
Distance	0.584	0.615	0.647
Reference	NCI/NTP TR-356	NCI/NTP TR-457	NCI/NTP TR-222

Model Applicability

Unknown features are fingerprint features in the query molecule, but not found or appearing too infrequently in the training set.

1. All properties and OPS components are within expected ranges.

Feature Contribution

Top features for positive contribution

Fingerprint	Bit/Smiles	Feature Structure	Score
FCFP_2	3	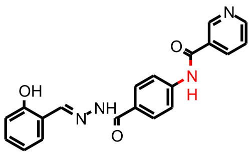 [*]N[*]	0.0737

FCFP_2	17	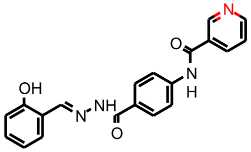 [*]:n:[*]	0.0441
FCFP_2	590925877	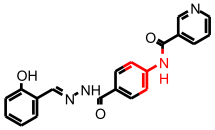 [*]N[c](:[cH]:[*]):[cH]:[*]	0.00762
**Top Features for negative contribution**			
**Fingerprint**	**Bit/Smiles**	**Feature Structure**	**Score**
FCFP_2	7	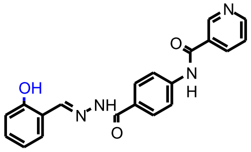 [*]O	-0.214
FCFP_2	-549108873	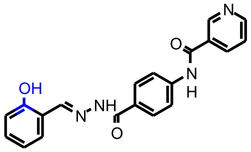 [*]:[c](:[*])O	-0.127
FCFP_2	1872154524	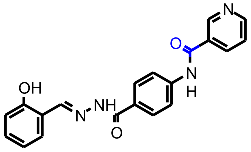 [*]C(=O)[*]	-0.105

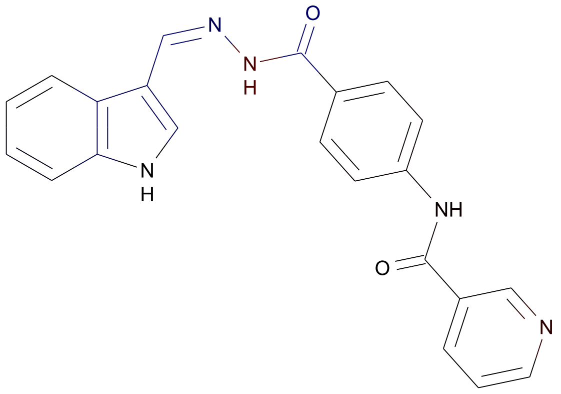

$C_{22}H_{17}N_5O_2$

Molecular Weight: 383.40268

ALogP: 2.611

Rotatable Bonds: 5

Acceptors: 4

Donors: 3

Model Prediction

Prediction: 0.181

Unit: g/kg_body_weight

Mahalanobis Distance: 8.67

Mahalanobis Distance p-value: 0.00386

Mahalanobis Distance: The Mahalanobis distance (MD) is a generalization of the Euclidean distance that accounts for correlations among the X properties. It is calculated as the distance to the center of the training data. The larger the MD, the less trustworthy the prediction.

Mahalanobis Distance p-value: The p-value gives the fraction of training data with an MD greater than or equal to the one for the given sample, assuming normally distributed data. The smaller the p-value, the less trustworthy the prediciton. For highly non-normal X properties (e.g., fingerprints), the MD p-value is wildly inaccurate.

Structural Similar Compounds

Name	PHENOLPHTHALEIN	SALICYLAZOSULFAPYRIDINE	FUROSEMIDE
Structure	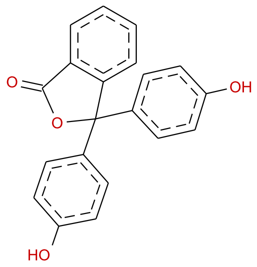	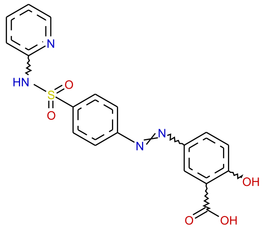	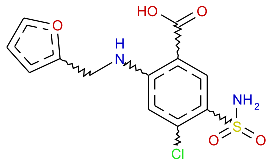
Actual Endpoint (-log C)	2.20184	3.375	4.04236
Predicted Endpoint (-log C)	2.8857	2.80292	2.8614
Distance	0.769	0.823	0.828
Reference	NCI/NTP TR-465	NCI/NTP TR-457	NCI/NTP TR-356

Model Applicability

Unknown features are fingerprint features in the query molecule, but not found or appearing too infrequently in the training set.

1. All properties and OPS components are within expected ranges.

Feature Contribution

Top features for positive contribution			
**Fingerprint**	**Bit/Smiles**	**Feature Structure**	**Score**
FCFP_2	3	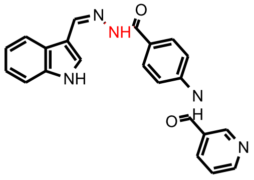 [*]N[*]	0.0737

FCFP_2	17	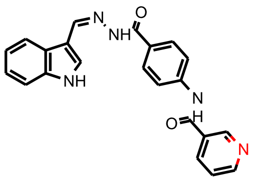 [*]:n:[*]	0.0441
FCFP_2	590925877	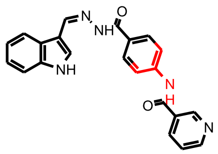 [*]N[c](:[cH]:[*]):[cH]:[*]	0.00762

Top Features for negative contribution

**Fingerprint**	**Bit/Smiles**	**Feature Structure**	**Score**
FCFP_2	1872154524	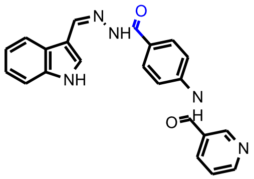 [*]C(=O)[*]	-0.105
FCFP_2	203677720	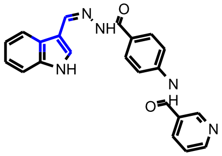 [*]C(=[*])[c](:[cH]:[*]):[cH]:[*]	-0.0829
FCFP_2	1	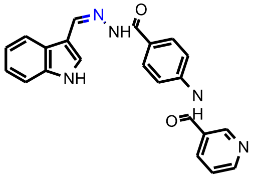 [*]=O	-0.0796

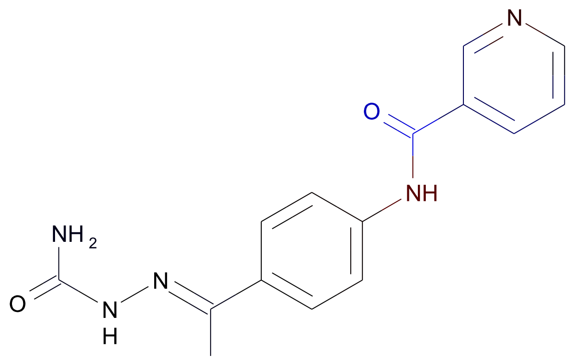

$C_{15}H_{15}N_5O_2$
Molecular Weight: 297.3119
ALogP: 0.423
Rotatable Bonds: 4
Acceptors: 4
Donors: 3

Model Prediction

Prediction: 0.144
Unit: g/kg_body_weight
Mahalanobis Distance: 7.79
Mahalanobis Distance p-value: 0.0408

Mahalanobis Distance: The Mahalanobis distance (MD) is a generalization of the Euclidean distance that accounts for correlations among the X properties. It is calculated as the distance to the center of the training data. The larger the MD, the less trustworthy the prediction.
Mahalanobis Distance p-value: The p-value gives the fraction of training data with an MD greater than or equal to the one for the given sample, assuming normally distributed data. The smaller the p-value, the less trustworthy the prediciton. For highly non-normal X properties (e.g., fingerprints), the MD p-value is wildly inaccurate.

Structural Similar Compounds

Name	FUROSEMIDE	DAPSONE	NITROFURAZONE
Structure	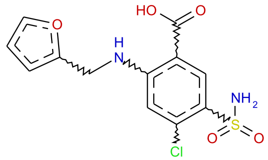	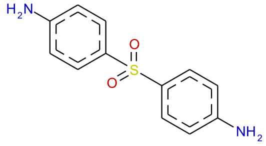	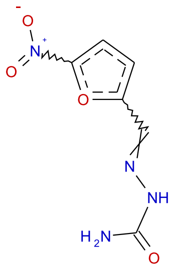
Actual Endpoint (-log C)	4.04236	3.66258	4.21779
Predicted Endpoint (-log C)	2.8614	3.26993	3.40885
Distance	0.491	0.541	0.558
Reference	NCI/NTP TR-356	NCI/NTP TR-20	NCI/NTP TR-337

Model Applicability

Unknown features are fingerprint features in the query molecule, but not found or appearing too infrequently in the training set.

1. All properties and OPS components are within expected ranges.

Feature Contribution

Top features for positive contribution			
**Fingerprint**	**Bit/Smiles**	**Feature Structure**	**Score**
FCFP_2	3	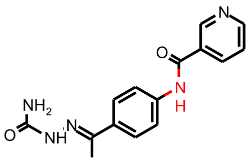 [*]N[*]	0.0737

FCFP_2	17	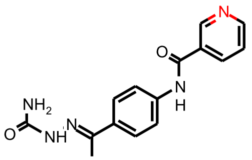 [*]:n:[*]	0.0441
FCFP_2	590925877	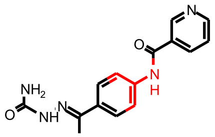 [*]N[c](:[cH]:[*]):[cH]:[*]	0.00762

Top Features for negative contribution

**Fingerprint**	**Bit/Smiles**	**Feature Structure**	**Score**
FCFP_2	1872154524	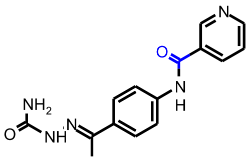 [*]C(=O)[*]	-0.105
FCFP_2	203677720	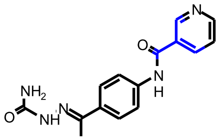 [*]C(=[*])[c](:[cH]:[*]):[cH]:[*]	-0.0829
FCFP_2	1	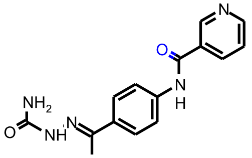 [*]=O	-0.0796

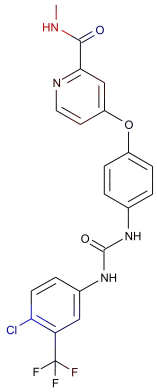

$C_{21}H_{16}ClF_3N_4O_3$
Molecular Weight: 464.82494
ALogP: 4.175
Rotatable Bonds: 6
Acceptors: 4
Donors: 3

Model Prediction

Prediction: 0.0885
Unit: g/kg_body_weight
Mahalanobis Distance: 12.4
Mahalanobis Distance p-value: 1.76e-009

Mahalanobis Distance: The Mahalanobis distance (MD) is a generalization of the Euclidean distance that accounts for correlations among the X properties. It is calculated as the distance to the center of the training data. The larger the MD, the less trustworthy the prediction.
Mahalanobis Distance p-value: The p-value gives the fraction of training data with an MD greater than or equal to the one for the given sample, assuming normally distributed data. The smaller the p-value, the less trustworthy the prediciton. For highly non-normal X properties (e.g., fingerprints), the MD p-value is wildly inaccurate.

Structural Similar Compounds

Name	FUROSEMIDE	PHENOLPHTHALEIN	DISPERSE YELLOW 3
Structure	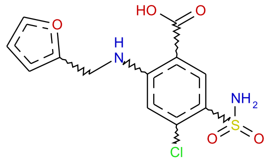	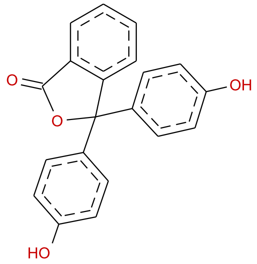	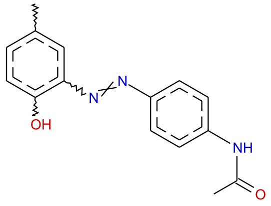
Actual Endpoint (-log C)	4.04236	2.20184	2.77703
Predicted Endpoint (-log C)	2.8614	2.8857	2.80195
Distance	0.741	0.780	0.799
Reference	NCI/NTP TR-356	NCI/NTP TR-465	NCI/NTP TR-222

Model Applicability

Unknown features are fingerprint features in the query molecule, but not found or appearing too infrequently in the training set.

1. All properties and OPS components are within expected ranges.

Feature Contribution

Top features for positive contribution			
**Fingerprint**	**Bit/Smiles**	**Feature Structure**	**Score**
FCFP_2	-885550502	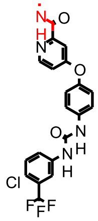 [*]C(=[*])NC	0.115

FCFP_2	3	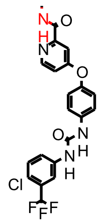 [*]N[*]	0.0737
FCFP_2	332760439	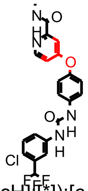 [*]O[c](:[cH]:[*]):[cH]:[*]	0.0611

Top Features for negative contribution

Fingerprint	Bit/Smiles	Feature Structure	Score
FCFP_2	71476542	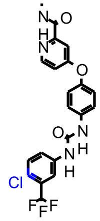 [*]:[c](:[*])Cl	-0.134
FCFP_2	1872154524	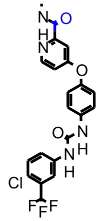 [*]C(=O)[*]	-0.105
FCFP_2	203677720	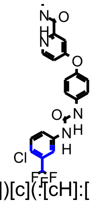 [*]C(=[*])[c](:[cH]:[*]):[cH]:[*]	-0.0829

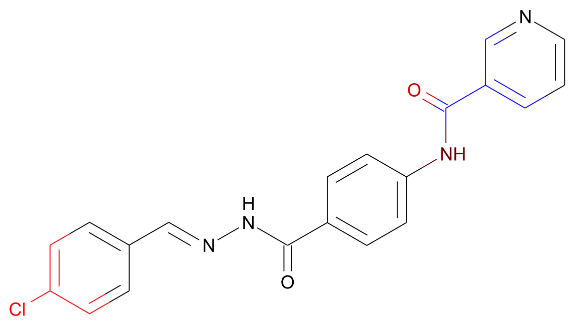

$C_{20}H_{15}ClN_4O_2$
Molecular Weight: 378.8117
ALogP: 2.981
Rotatable Bonds: 5
Acceptors: 4
Donors: 2

Model Prediction

Prediction: 0.00712
Unit: g/kg_body_weight
Mahalanobis Distance: 7.78
Mahalanobis Distance p-value: 0.00367

Mahalanobis Distance: The Mahalanobis distance (MD) is a generalization of the Euclidean distance that accounts for correlations among the X properties. It is calculated as the distance to the center of the training data. The larger the MD, the less trustworthy the prediction.
Mahalanobis Distance p-value: The p-value gives the fraction of training data with an MD greater than or equal to the one for the given sample, assuming normally distributed data. The smaller the p-value, the less trustworthy the prediciton. For highly non-normal X properties (e.g., fingerprints), the MD p-value is wildly inaccurate.

Structural Similar Compounds

Name	SULFISOOXAZOLE	OCHRATOXIN	PROBENECID
Structure	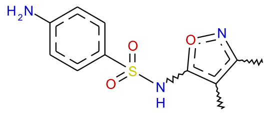	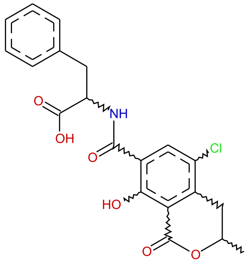	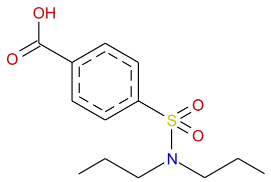
Actual Endpoint (-log C)	2.82494	6.28396	2.85333
Predicted Endpoint (-log C)	3.0705	5.12358	2.4258
Distance	0.787	0.805	1.002
Reference	NCI/NTP TR-138	NCI/NTP TR-358	NCI/NTP TR-395

Model Applicability

Unknown features are fingerprint features in the query molecule, but not found or appearing too infrequently in the training set.

1. Num_AromaticRings out of range. Value: 3. Training min, max, mean, SD: 0, 2, 0.5625, 0.693.
2. OPS PC7 out of range. Value: -3.6692. Training min, max, SD, explained variance: -2.8003, 2.9332, 1.16, 0.0416.
3. Unknown FCFP_2 feature: -885520711: [*]C(=[*])NN=[*]
4. Unknown FCFP_2 feature: 581019816: [*]N\N=C\[*]

Feature Contribution

Top features for positive contribution

Fingerprint	Bit/Smiles	Feature Structure	Score
FCFP_2	32	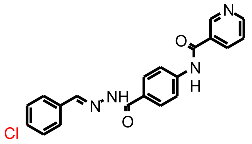 [*]Cl	0.526

FCFP_2	1	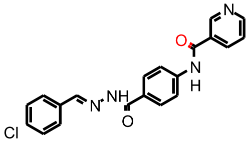<br>[*]=O	0.511
FCFP_2	367998008	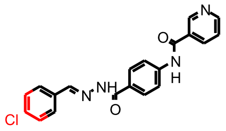<br>[*]:[cH]:[c](Cl):[cH]:[*]	0.413

Top Features for negative contribution

Fingerprint	Bit/Smiles	Feature Structure	Score
FCFP_2	203677720	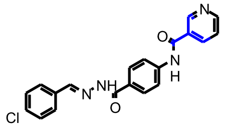<br>[*]C(=[*])[c](:[cH]:[*]):[cH]:[*]	-0.406
FCFP_2	1872154524	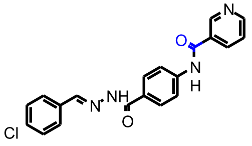<br>[*]C(=O)[*]	-0.307
FCFP_2	0	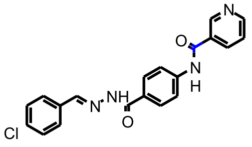<br>[*]C(=[*])[*]	-0.29

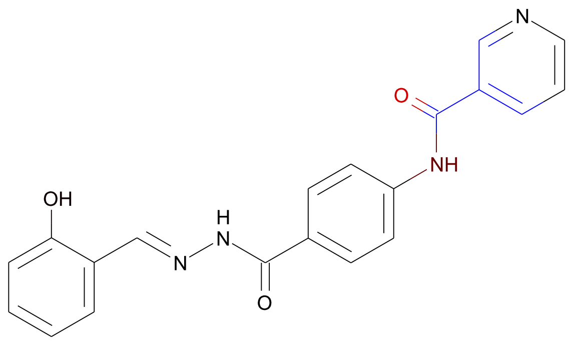

$C_{20}H_{16}N_4O_3$
Molecular Weight: 360.36604
ALogP: 2.075
Rotatable Bonds: 5
Acceptors: 5
Donors: 3

Model Prediction

Prediction: 0.0342
Unit: g/kg_body_weight
Mahalanobis Distance: 10.4
Mahalanobis Distance p-value: 1.65e-006

Mahalanobis Distance: The Mahalanobis distance (MD) is a generalization of the Euclidean distance that accounts for correlations among the X properties. It is calculated as the distance to the center of the training data. The larger the MD, the less trustworthy the prediction.
Mahalanobis Distance p-value: The p-value gives the fraction of training data with an MD greater than or equal to the one for the given sample, assuming normally distributed data. The smaller the p-value, the less trustworthy the prediciton. For highly non-normal X properties (e.g., fingerprints), the MD p-value is wildly inaccurate.

Structural Similar Compounds

Name	OCHRATOXIN	SULFISOOXAZOLE	HC RED 3
Structure	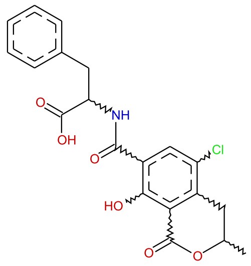	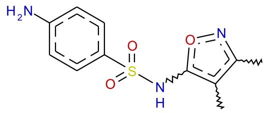	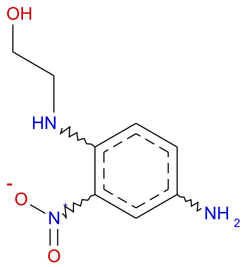
Actual Endpoint (-log C)	6.28396	2.82494	2.59592
Predicted Endpoint (-log C)	5.12358	3.0705	3.285
Distance	0.662	0.745	0.963
Reference	NCI/NTP TR-358	NCI/NTP TR-138	NCI/NTP TR-281

Model Applicability

Unknown features are fingerprint features in the query molecule, but not found or appearing too infrequently in the training set.

1. Num_AromaticRings out of range. Value: 3. Training min, max, mean, SD: 0, 2, 0.5625, 0.693.
2. Unknown FCFP_2 feature: -885520711: [*]C(=[*])NN=[*]
3. Unknown FCFP_2 feature: 581019816: [*]N\N=C\[*]

Feature Contribution

Top features for positive contribution			
**Fingerprint**	**Bit/Smiles**	**Feature Structure**	**Score**
FCFP_2	1	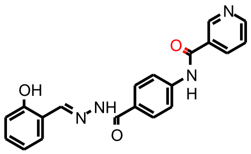 [*]=O	0.511

FCFP_2	3	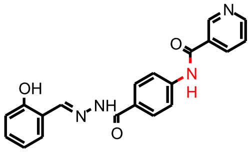<br>[*]N[*]	0.104
FCFP_2	7	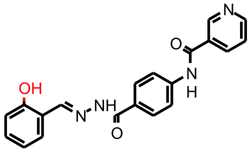<br>[*]O	0.0144
**Top Features for negative contribution**			
**Fingerprint**	**Bit/Smiles**	**Feature Structure**	**Score**
FCFP_2	203677720	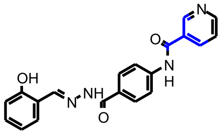<br>[*]C(=[*])[c](:[cH]:[*]):[cH]:[*]	-0.406
FCFP_2	1872154524	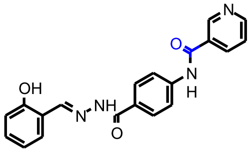<br>[*]C(=O)[*]	-0.307
FCFP_2	0	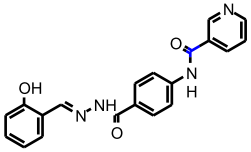<br>[*]C(=[*])[*]	-0.29

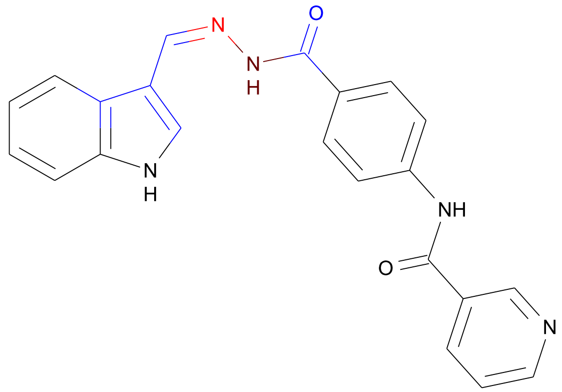

$C_{22}H_{17}N_5O_2$
Molecular Weight: 383.40268
ALogP: 2.611
Rotatable Bonds: 5
Acceptors: 4
Donors: 3

Model Prediction

Prediction: 0.0813
Unit: g/kg_body_weight
Mahalanobis Distance: 13.2
Mahalanobis Distance p-value: 1.84e-010

Mahalanobis Distance: The Mahalanobis distance (MD) is a generalization of the Euclidean distance that accounts for correlations among the X properties. It is calculated as the distance to the center of the training data. The larger the MD, the less trustworthy the prediction.
Mahalanobis Distance p-value: The p-value gives the fraction of training data with an MD greater than or equal to the one for the given sample, assuming normally distributed data. The smaller the p-value, the less trustworthy the prediciton. For highly non-normal X properties (e.g., fingerprints), the MD p-value is wildly inaccurate.

Structural Similar Compounds

Name	SULFISOOXAZOLE	OCHRATOXIN	HC RED 3
Structure	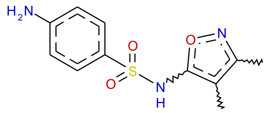	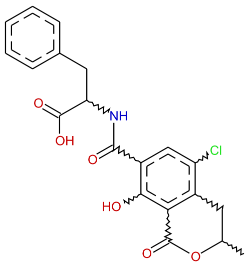	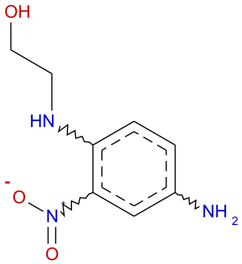
Actual Endpoint (-log C)	2.82494	6.28396	2.59592
Predicted Endpoint (-log C)	3.0705	5.12358	3.285
Distance	0.973	0.974	1.241
Reference	NCI/NTP TR-138	NCI/NTP TR-358	NCI/NTP TR-281

Model Applicability

Unknown features are fingerprint features in the query molecule, but not found or appearing too infrequently in the training set.

1. Num_AromaticRings out of range. Value: 4. Training min, max, mean, SD: 0, 2, 0.5625, 0.693.
2. Unknown FCFP_2 feature: 19: [*]:[nH]:[*]
3. Unknown FCFP_2 feature: 307448885: [*]:[c]1:[*]:[*]:[nH]:[c]:1:c:[*]
4. Unknown FCFP_2 feature: 1618184456: [*][c]1:[*]:[*]:[nH]:c:1
5. Unknown FCFP_2 feature: 2005402822: [*]:[c]1:[*]:[*]:c:[nH]:1
6. Unknown FCFP_2 feature: 581019816: [*]N\N=C\[*]
7. Unknown FCFP_2 feature: -885520711: [*]C(=[*])NN=[*]

Feature Contribution

Top features for positive contribution			
**Fingerprint**	**Bit/Smiles**	**Feature Structure**	**Score**

FCFP_2	1	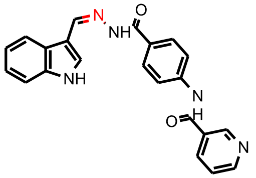 [*]=O	0.511
FCFP_2	3	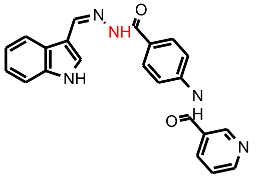 [*]N[*]	0.104

Top Features for negative contribution

**Fingerprint**	**Bit/Smiles**	**Feature Structure**	**Score**
FCFP_2	203677720	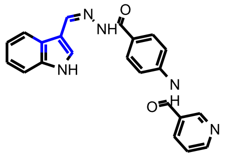 [*]C(=[*])[c](:[cH]:[*]):[cH]:[*]	-0.406
FCFP_2	1872154524	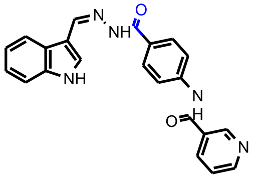 [*]C(=O)[*]	-0.307
FCFP_2	0	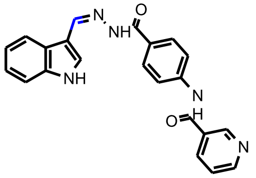 [*]C(=[*])[*]	-0.29

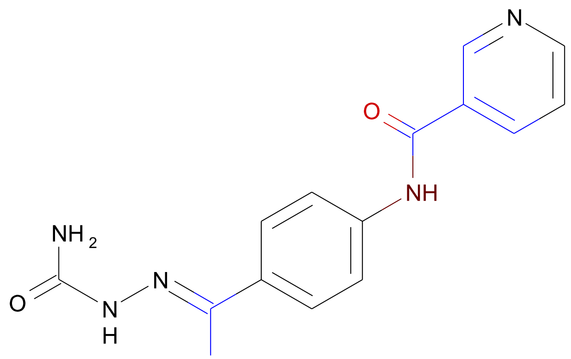

$C_{15}H_{15}N_5O_2$
Molecular Weight: 297.3119
ALogP: 0.423
Rotatable Bonds: 4
Acceptors: 4
Donors: 3

Model Prediction

Prediction: 1.08
Unit: g/kg_body_weight
Mahalanobis Distance: 7.61
Mahalanobis Distance p-value: 0.0057

Mahalanobis Distance: The Mahalanobis distance (MD) is a generalization of the Euclidean distance that accounts for correlations among the X properties. It is calculated as the distance to the center of the training data. The larger the MD, the less trustworthy the prediction.
Mahalanobis Distance p-value: The p-value gives the fraction of training data with an MD greater than or equal to the one for the given sample, assuming normally distributed data. The smaller the p-value, the less trustworthy the prediciton. For highly non-normal X properties (e.g., fingerprints), the MD p-value is wildly inaccurate.

Structural Similar Compounds

Name	SULFISOOXAZOLE	HC RED 3	OCHRATOXIN
Structure	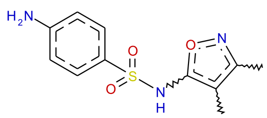	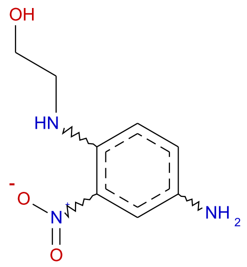	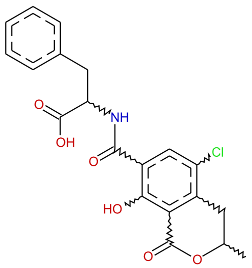
Actual Endpoint (-log C)	2.82494	2.59592	6.28396
Predicted Endpoint (-log C)	3.0705	3.285	5.12358
Distance	0.539	0.627	0.810
Reference	NCI/NTP TR-138	NCI/NTP TR-281	NCI/NTP TR-358

Model Applicability

Unknown features are fingerprint features in the query molecule, but not found or appearing too infrequently in the training set.

1. All properties and OPS components are within expected ranges.
2. Unknown FCFP_2 feature: -1549192822: [*]\N=C(/C)\[c](:[*]):[*]
3. Unknown FCFP_2 feature: 581019816: [*]N\N=C\[*]
4. Unknown FCFP_2 feature: -885520711: [*]C(=[*])NN=[*]
5. Unknown FCFP_2 feature: 1499521844: [*]NC(=O)N

Feature Contribution

Top features for positive contribution			
Fingerprint	Bit/Smiles	Feature Structure	Score
FCFP_2	1	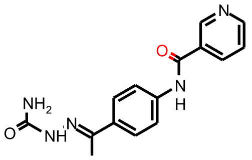 [*]=O	0.511

FCFP_2	3	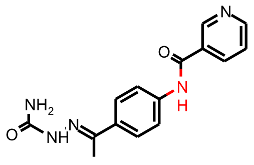 [*]N[*]	0.104

Top Features for negative contribution

Fingerprint	Bit/Smiles	Feature Structure	Score
FCFP_2	136597326	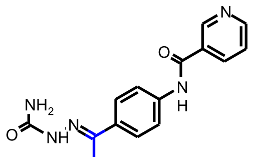 [*]C(=[*])C	-0.489
FCFP_2	203677720	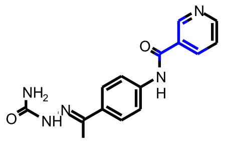 [*]C(=[*])[c](:[cH]:[*]):[cH]:[*]	-0.406
FCFP_2	1872154524	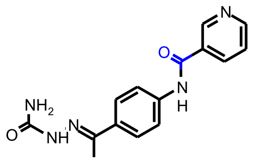 [*]C(=O)[*]	-0.307

Sorafenib

TOPKAT_Rat_Maximum_Tolerated_Dose_Gavage

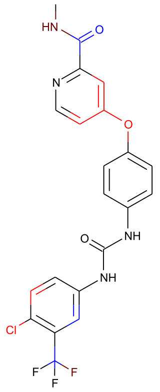

$C_{21}H_{16}ClF_3N_4O_3$
Molecular Weight: 464.82494
ALogP: 4.175
Rotatable Bonds: 6
Acceptors: 4
Donors: 3

Model Prediction

Prediction: 0.000918
Unit: g/kg_body_weight
Mahalanobis Distance: 12.2
Mahalanobis Distance p-value: 4.69e-009

Mahalanobis Distance: The Mahalanobis distance (MD) is a generalization of the Euclidean distance that accounts for correlations among the X properties. It is calculated as the distance to the center of the training data. The larger the MD, the less trustworthy the prediction.
Mahalanobis Distance p-value: The p-value gives the fraction of training data with an MD greater than or equal to the one for the given sample, assuming normally distributed data. The smaller the p-value, the less trustworthy the prediciton. For highly non-normal X properties (e.g., fingerprints), the MD p-value is wildly inaccurate.

Structural Similar Compounds

Name	OCHRATOXIN	SULFISOOXAZOLE	PENICILLIN VK
Structure	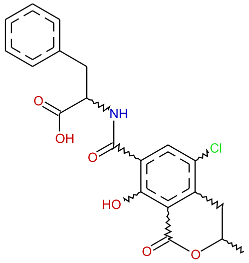	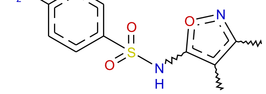	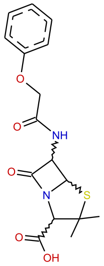
Actual Endpoint (-log C)	6.28396	2.82494	2.54455
Predicted Endpoint (-log C)	5.12358	3.0705	3.9702
Distance	0.758	0.997	1.159
Reference	NCI/NTP TR-358	NCI/NTP TR-138	NCI/NTP TR-336

Model Applicability

Unknown features are fingerprint features in the query molecule, but not found or appearing too infrequently in the training set.

1. Molecular_Weight out of range. Value: 464.82. Training min, max, mean, SD: 68.074, 434.63, 171.13, 85.06.
2. Num_AromaticRings out of range. Value: 3. Training min, max, mean, SD: 0, 2, 0.5625, 0.693.
3. OPS PC5 out of range. Value: -3.5737. Training min, max, SD, explained variance: -3.4, 4.1587, 1.489, 0.0686.
4. OPS PC7 out of range. Value: -3.8342. Training min, max, SD, explained variance: -2.8003, 2.9332, 1.16, 0.0416.
5. Unknown FCFP_2 feature: 136686699: [*]NC
6. Unknown FCFP_2 feature: 1499521844: [*]NC(=O)N
7. Unknown FCFP_2 feature: -1029533685: [*]:[c](:[*])C(F)(F)F

Feature Contribution

Top features for positive contribution			
Fingerprint	Bit/Smiles	Feature Structure	Score

FCFP_2	332760439	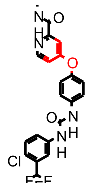 [*]O[c](:[cH]:[*]):[cH]:[*]	0.672
FCFP_2	32	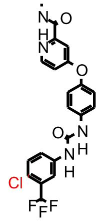 [*]Cl	0.526
FCFP_2	1	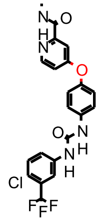 [*]=O	0.511
**Top Features for negative contribution**			
**Fingerprint**	**Bit/Smiles**	**Feature Structure**	**Score**
FCFP_2	203677720	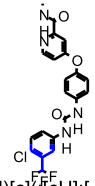 [*]C(=[*])[c](:[cH]:[*]):[cH]:[*]	-0.406
FCFP_2	1872154524	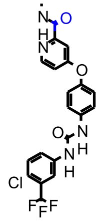 [*]C(=O)[*]	-0.307

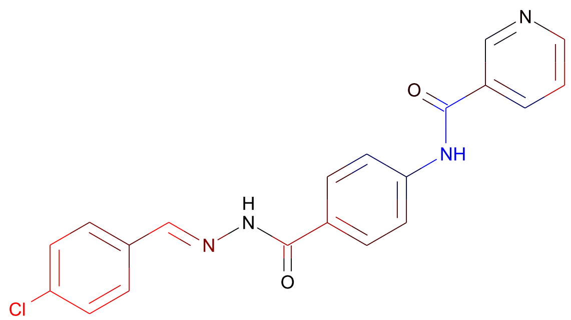

$C_{20}H_{15}ClN_4O_2$

Molecular Weight: 378.8117

ALogP: 2.981

Rotatable Bonds: 5

Acceptors: 4

Donors: 2

Structural Similar Compounds

Name	FLUBENDAZOLE	CARBAMIC ACID; N-(5-BENZOYLBENZIMIDAZOL-2-YL)-; METHYL ESTER	NAPTALAM
Structure	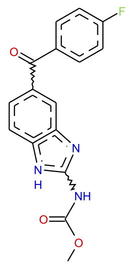	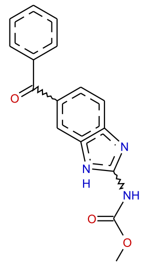	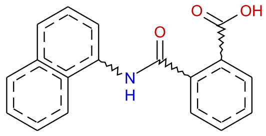
Actual Endpoint (-log C)	2.088	2.617	1.551
Predicted Endpoint (-log C)	2.69288	2.2368	1.89036
Distance	0.502	0.547	0.607
Reference	YRTMA6 9;11;78	IYKEDH 19;735;88	FMCHA2 -;C206;89

Model Prediction

Prediction: 1.32

Unit: g/kg_body_weight

Mahalanobis Distance: 18.5

Mahalanobis Distance p-value: 6.52e-005

Mahalanobis Distance: The Mahalanobis distance (MD) is a generalization of the Euclidean distance that accounts for correlations among the X properties. It is calculated as the distance to the center of the training data. The larger the MD, the less trustworthy the prediction.
Mahalanobis Distance p-value: The p-value gives the fraction of training data with an MD greater than or equal to the one for the given sample, assuming normally distributed data. The smaller the p-value, the less trustworthy the prediciton. For highly non-normal X properties (e.g., fingerprints), the MD p-value is wildly inaccurate.

Model Applicability

Unknown features are fingerprint features in the query molecule, but not found or appearing too infrequently in the training set.

1. All properties and OPS components are within expected ranges.
2. Unknown FCFP_6 feature: 16: [*]:[cH]:[*]
3. Unknown FCFP_6 feature: 1618154665: [*]:[cH]:[cH]:[cH]:[*]
4. Unknown FCFP_6 feature: 1747237384: [*]:[cH]:n:[cH]:[*]
5. Unknown FCFP_6 feature: -885520711: [*]C(=[*])NN=[*]
6. Unknown FCFP_6 feature: 581019816: [*]N\N=C\[*]
7. Unknown FCFP_6 feature: -2100785893: [*]\N=C\[c](:[*]):[*]
8. Unknown FCFP_6 feature: 71476542: [*]:[c](:[*])Cl

Feature Contribution

Top features for positive contribution			
**Fingerprint**	**Bit/Smiles**	**Feature Structure**	**Score**

ECFP_6	642810091	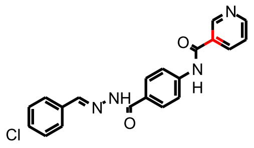 [*][c](:[*]):[*]	0.281
ECFP_6	-1897341097	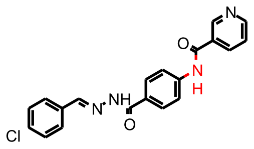 [*]N[*]	0.216
FCFP_6	-149636017	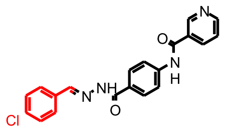 [*]=C[c]1:[cH]:[cH]:[c](Cl):[cH]:[cH]:1	0.193
**Top Features for negative contribution**			
**Fingerprint**	**Bit/Smiles**	**Feature Structure**	**Score**
ECFP_6	-817402818	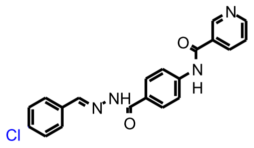 [*]Cl	-0.263
ECFP_6	655739385	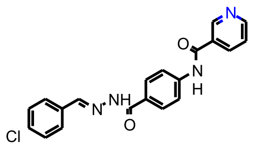 [*]:n:[*]	-0.239

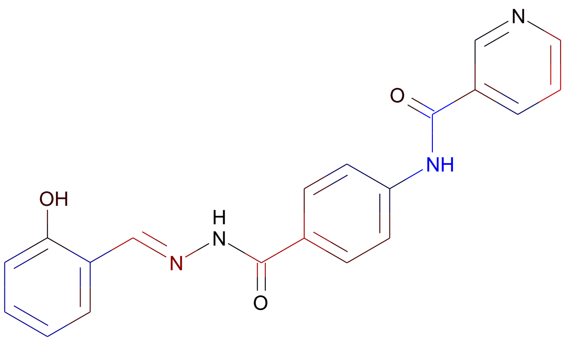

$C_{20}H_{16}N_4O_3$

Molecular Weight: 360.36604

ALogP: 2.075

Rotatable Bonds: 5

Acceptors: 5

Donors: 3

Model Prediction

Prediction: 1.53

Unit: g/kg_body_weight

Mahalanobis Distance: 18.9

Mahalanobis Distance p-value: 8.06e-006

Mahalanobis Distance: The Mahalanobis distance (MD) is a generalization of the Euclidean distance that accounts for correlations among the X properties. It is calculated as the distance to the center of the training data. The larger the MD, the less trustworthy the prediction.
Mahalanobis Distance p-value: The p-value gives the fraction of training data with an MD greater than or equal to the one for the given sample, assuming normally distributed data. The smaller the p-value, the less trustworthy the prediciton. For highly non-normal X properties (e.g., fingerprints), the MD p-value is wildly inaccurate.

Structural Similar Compounds

Name	SULFAQUINOXALINE	CARBAMIC ACID; N-(5-BENZOYLBENZIMIDAZOL-2-YL)-; METHYL ESTER	ANTHRAQUINONE; 1-[(2-HYDROXYETHYL)AMINO]-4-(METHYLAMINO)-
Structure	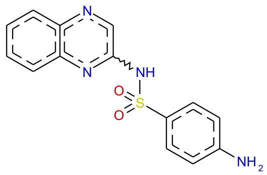	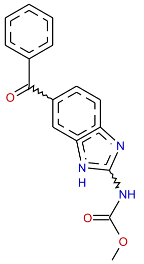	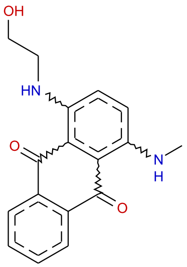
Actual Endpoint (-log C)	2.341	2.617	1.995
Predicted Endpoint (-log C)	2.42674	2.2368	1.85368
Distance	0.633	0.665	0.667
Reference	MahWM# 16NOV82	IYKEDH 19;735;88	85JCAE -;1325;86

Model Applicability

Unknown features are fingerprint features in the query molecule, but not found or appearing too infrequently in the training set.

1. All properties and OPS components are within expected ranges.
2. Unknown FCFP_6 feature: 16: [*]:[cH]:[*]
3. Unknown FCFP_6 feature: 1618154665: [*]:[cH]:[cH]:[cH]:[*]
4. Unknown FCFP_6 feature: 1747237384: [*]:[cH]:n:[cH]:[*]
5. Unknown FCFP_6 feature: -885520711: [*]C(=[*])NN=[*]
6. Unknown FCFP_6 feature: 581019816: [*]N\N=C\[*]
7. Unknown FCFP_6 feature: -2100785893: [*]\N=C\[c](:[*]):[*]
8. Unknown FCFP_6 feature: 74595001: [*][c](:[*]):[c](O):[cH]:[*]
9. Unknown FCFP_6 feature: -549108873: [*]:[c](:[*])O

Feature Contribution

Top features for positive contribution

Fingerprint	Bit/Smiles	Feature Structure	Score

ECFP_6	642810091	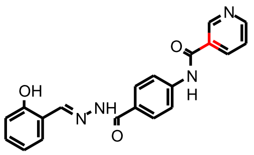 [*][c](:[*]):[*]	0.281
ECFP_6	-1897341097	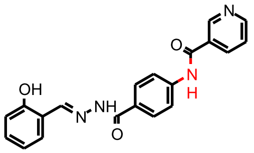 [*]N[*]	0.216
ECFP_6	1444581947	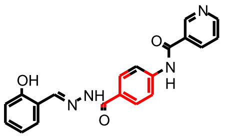 [*]C(=[*])[c]1:[cH]:[*]:[c]([*]):[cH]:[cH]:1	0.163

Top Features for negative contribution

Fingerprint	Bit/Smiles	Feature Structure	Score
ECFP_6	655739385	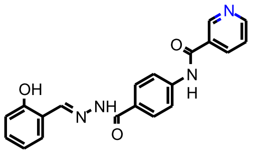 [*]:n:[*]	-0.239
FCFP_6	946589555	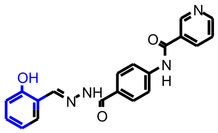 [*][c]1:[*]:[cH]:[cH] :[cH]:[c]:1O	-0.204

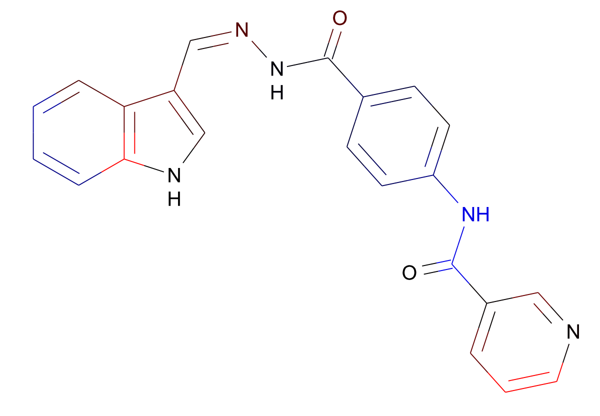

$C_{22}H_{17}N_5O_2$
Molecular Weight: 383.40268
ALogP: 2.611
Rotatable Bonds: 5
Acceptors: 4
Donors: 3

Model Prediction

Prediction: 1.86
Unit: g/kg_body_weight
Mahalanobis Distance: 21.6
Mahalanobis Distance p-value: 1.52e-014

Mahalanobis Distance: The Mahalanobis distance (MD) is a generalization of the Euclidean distance that accounts for correlations among the X properties. It is calculated as the distance to the center of the training data. The larger the MD, the less trustworthy the prediction.
Mahalanobis Distance p-value: The p-value gives the fraction of training data with an MD greater than or equal to the one for the given sample, assuming normally distributed data. The smaller the p-value, the less trustworthy the prediciton. For highly non-normal X properties (e.g., fingerprints), the MD p-value is wildly inaccurate.

Structural Similar Compounds

Name	CARBAMIC ACID; N-(5-BENZOYLBENZIMIDAZOL-2-YL)-; METHYL ESTER	FLUBENDAZOLE	SULFAQUINOXALINE
Structure	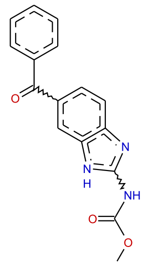	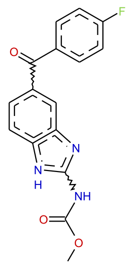	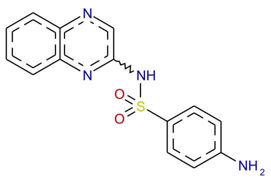
Actual Endpoint (-log C)	2.617	2.088	2.341
Predicted Endpoint (-log C)	2.2368	2.69288	2.42674
Distance	0.703	0.704	0.759
Reference	IYKEDH 19;735;88	YRTMA6 9;11;78	MahWM# 16NOV82

Model Applicability

Unknown features are fingerprint features in the query molecule, but not found or appearing too infrequently in the training set.

1. All properties and OPS components are within expected ranges.
2. Unknown FCFP_6 feature: 16: [*]:[cH]:[*]
3. Unknown FCFP_6 feature: 19: [*]:[nH]:[*]
4. Unknown FCFP_6 feature: 307448885: [*]:[cH]:[c]1:[nH]:[*]:[*]:[c]:1:[*]
5. Unknown FCFP_6 feature: 1618184456: [*][c]1:[*]:[*]:[nH]:[cH]:1
6. Unknown FCFP_6 feature: 2005402822: [*]:[c]1:[*]:[*]:[cH]:[nH]:1
7. Unknown FCFP_6 feature: 1618154665: [*]:[cH]:[cH]:[cH]:[*]
8. Unknown FCFP_6 feature: -2100785893: [*]\N=C\[c](:[*]):[*]
9. Unknown FCFP_6 feature: 581019816: [*]N\N=C\[*]
10. Unknown FCFP_6 feature: -885520711: [*]C(=[*])NN=[*]
11. Unknown FCFP_6 feature: 1747237384: [*]:[cH]:n:[cH]:[*]

Feature Contribution

Top features for positive contribution			
Fingerprint	Bit/Smiles	Feature Structure	Score

ECFP_6	642810091	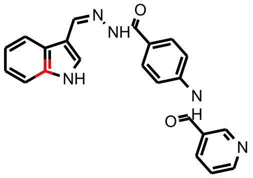 [*][c](:[*]):[*]	0.281
ECFP_6	-1897341097	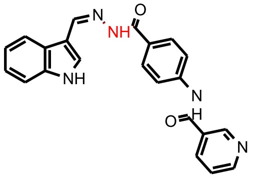 [*]N[*]	0.216
ECFP_6	1444581947	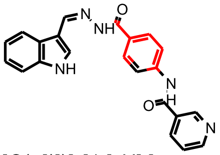 [*]C(=[*])[c]1:[cH]:[*]:[c]([*]):[cH]:[cH]:1	0.163

Top Features for negative contribution

Fingerprint	Bit/Smiles	Feature Structure	Score
ECFP_6	655739385	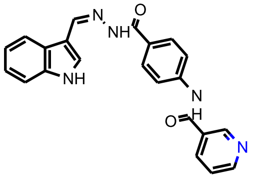 [*]:n:[*]	-0.239
FCFP_6	-1549103449	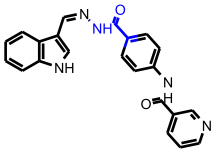 [*]NC(=O)[c](:[*]):[*]	-0.117

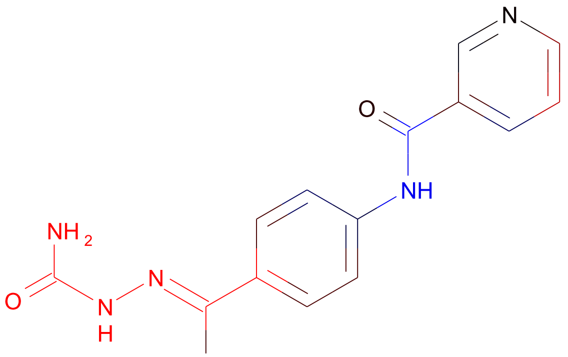

$C_{15}H_{15}N_5O_2$
Molecular Weight: 297.3119
ALogP: 0.423
Rotatable Bonds: 4
Acceptors: 4
Donors: 3

Model Prediction

Prediction: 0.744
Unit: g/kg_body_weight
Mahalanobis Distance: 21.2
Mahalanobis Distance p-value: 5.36e-013

Mahalanobis Distance: The Mahalanobis distance (MD) is a generalization of the Euclidean distance that accounts for correlations among the X properties. It is calculated as the distance to the center of the training data. The larger the MD, the less trustworthy the prediction.
Mahalanobis Distance p-value: The p-value gives the fraction of training data with an MD greater than or equal to the one for the given sample, assuming normally distributed data. The smaller the p-value, the less trustworthy the prediciton. For highly non-normal X properties (e.g., fingerprints), the MD p-value is wildly inaccurate.

Structural Similar Compounds

Name	SULFAFURAZOLE	SULFAPYRIDINE	SULFAMETHOXAZOLE
Structure	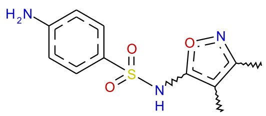	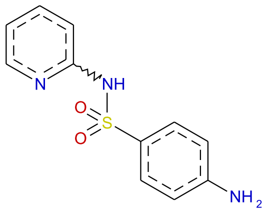	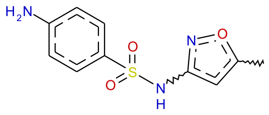
Actual Endpoint (-log C)	1.427	1.198	1.611
Predicted Endpoint (-log C)	1.37011	2.06465	1.72769
Distance	0.575	0.582	0.582
Reference	NIIRDN 6;391;82	QJPPAL 11;217;38	TXAPA9 18;185;71

Model Applicability

Unknown features are fingerprint features in the query molecule, but not found or appearing too infrequently in the training set.

1. All properties and OPS components are within expected ranges.
2. Unknown ECFP_2 feature: 128986386: [*]\N=C(/C)\[c](:[*]):[*]
3. Unknown FCFP_6 feature: 16: [*]:[cH]:[*]
4. Unknown FCFP_6 feature: 1618154665: [*]:[cH]:[cH]:[cH]:[*]
5. Unknown FCFP_6 feature: 1747237384: [*]:[cH]:n:[cH]:[*]
6. Unknown FCFP_6 feature: 581019816: [*]N\N=C\[*]
7. Unknown FCFP_6 feature: -885520711: [*]C(=[*])NN=[*]

Feature Contribution

Top features for positive contribution

Fingerprint	Bit/Smiles	Feature Structure	Score

ECFP_6	642810091	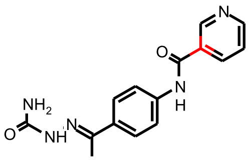 [*][c](:[*]):[*]	0.281
FCFP_6	1499521844	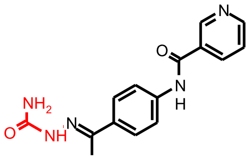 [*]NC(=O)N	0.258
ECFP_6	-1897341097	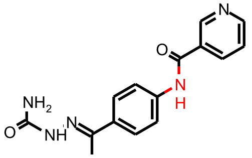 [*]N[*]	0.216
**Top Features for negative contribution**			
**Fingerprint**	**Bit/Smiles**	**Feature Structure**	**Score**
ECFP_6	655739385	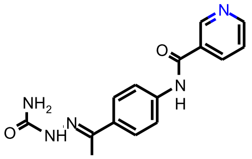 [*]:n:[*]	-0.239
ECFP_6	734603939	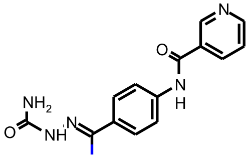 [*]C	-0.201

Sorafenib

TOPKAT_Rat_Oral_LD50

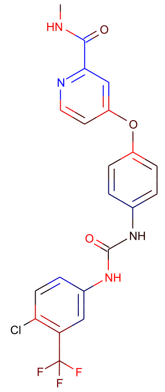

$C_{21}H_{16}ClF_3N_4O_3$

Molecular Weight: 464.82494

ALogP: 4.175

Rotatable Bonds: 6

Acceptors: 4

Donors: 3

Structural Similar Compounds

Name	FLUBENDAZOLE	PHOSPHORAMIDOTHIOIC ACID; ACETIMIDOYL-; O;O-bis-(p-CHLOROPHENYL)ESTER	BEZAFIBRATE
Structure	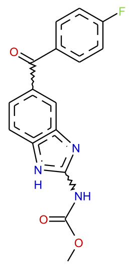	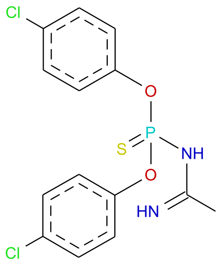	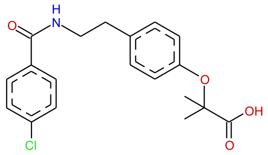
Actual Endpoint (-log C)	2.088	5.006	1.946
Predicted Endpoint (-log C)	2.69288	3.23989	2.54395
Distance	0.697	0.703	0.721
Reference	YRTMA6 9;11;78	FMCHA2 -;C149;89	ARZNAD 30;2023;80

Model Prediction

Prediction: 0.823

Unit: g/kg_body_weight

Mahalanobis Distance: 21

Mahalanobis Distance p-value: 1.93e-012

Mahalanobis Distance: The Mahalanobis distance (MD) is a generalization of the Euclidean distance that accounts for correlations among the X properties. It is calculated as the distance to the center of the training data. The larger the MD, the less trustworthy the prediction.
Mahalanobis Distance p-value: The p-value gives the fraction of training data with an MD greater than or equal to the one for the given sample, assuming normally distributed data. The smaller the p-value, the less trustworthy the prediciton. For highly non-normal X properties (e.g., fingerprints), the MD p-value is wildly inaccurate.

Model Applicability

Unknown features are fingerprint features in the query molecule, but not found or appearing too infrequently in the training set.

1. All properties and OPS components are within expected ranges.
2. Unknown FCFP_6 feature: 16: [*]:[cH]:[*]
3. Unknown FCFP_6 feature: 1618154665: [*]:[cH]:[cH]:[cH]:[*]
4. Unknown FCFP_6 feature: 1747237384: [*]:[cH]:n:[cH]:[*]
5. Unknown FCFP_6 feature: 136686699: [*]NC
6. Unknown FCFP_6 feature: 71476542: [*]:[c](:[*])Cl

Feature Contribution

Top features for positive contribution			
Fingerprint	Bit/Smiles	Feature Structure	Score

FCFP_6	71953198	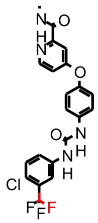 [*]C([*])([*])F	0.392
ECFP_6	-1046436026	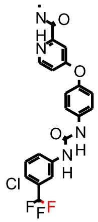 [*]F	0.349
ECFP_6	642810091	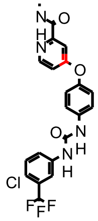 [*][c](:[*]):[*]	0.281

Top Features for negative contribution

Fingerprint	Bit/Smiles	Feature Structure	Score
ECFP_6	226796801	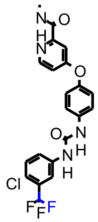 [*]C([*])([*])F	-0.32
ECFP_6	-817402818	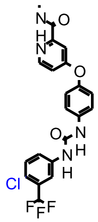 [*]Cl	-0.263
